# Identification of Host Kinase Genes Required for Influenza Virus Replication and the Regulatory Role of MicroRNAs

**DOI:** 10.1371/journal.pone.0066796

**Published:** 2013-06-21

**Authors:** Abhijeet Bakre, Lauren E. Andersen, Victoria Meliopoulos, Keegan Coleman, Xiuzhen Yan, Paula Brooks, Jackelyn Crabtree, S. Mark Tompkins, Ralph A. Tripp

**Affiliations:** Department of Infectious Diseases, University of Georgia, Athens, Georgia, United States of America; University of Hong Kong, Hong Kong

## Abstract

Human protein kinases (HPKs) have profound effects on cellular responses. To better understand the role of HPKs and the signaling networks that influence influenza virus replication, a small interfering RNA (siRNA) screen of 720 HPKs was performed. From the screen, 17 HPKs (NPR2, MAP3K1, DYRK3, EPHA6, TPK1, PDK2, EXOSC10, NEK8, PLK4, SGK3, NEK3, PANK4, ITPKB, CDC2L5 (CDK13), CALM2, PKN3, and HK2) were validated as essential for A/WSN/33 influenza virus replication, and 6 HPKs (CDK13, HK2, NEK8, PANK4, PLK4 and SGK3) were identified as vital for both A/WSN/33 and A/New Caledonia/20/99 influenza virus replication. These HPKs were found to affect multiple host pathways and regulated by miRNAs induced during infection. Using a panel of miRNA agonists and antagonists, miR-149* was found to regulate NEK8 expression, miR-548d-3p was found to regulate MAPK1 transcript expression, and miRs -1228 and -138 to regulate CDK13 expression. Up-regulation of miR-34c induced PLK4 transcript and protein expression and enhanced influenza virus replication, while miR-34c inhibition reduced viral replication. These findings identify HPKs important for influenza viral replication and show the miRNAs that govern their expression.

## Introduction

Influenza A viruses are ubiquitous, causing acute respiratory disease and substantial morbidity and mortality each year [1]–[3]. Although vaccination is central for controlling infection, treatment or prophylaxis with licensed antiviral drugs has been shown have >80% efficacy against the development of illness during inter-pandemic influenza periods [4]. Unfortunately, influenza has rapidly developed resistance to most antiviral drugs [5]–[8]. Given this and the difficulties with seasonal and pandemic influenza vaccine development [9], [10], there is a need for new disease intervention strategies.

Influenza A viruses belong to the family *Orthomyxoviridae*, are enveloped, and have an eight segmented, negative-sense, single-stranded RNA genome that encodes up to 11 proteins [11]. The viral envelope contains the surface glycoproteins and antigenic determinants, hemagglutinin (HA) and neuraminidase (NA), as well as the membrane ion channel protein, M2. Within the virion, the matrix protein (M1) provides structure and secures the viral ribonucleoprotein (vRNP) complexes consisting of viral RNA coupled to nucleoprotein (NP) and the three polymerase proteins (PB1, PB2 and PA). The remaining viral proteins include the nonstructural proteins, NS1 and NS2, and the recently identified PB1-F2 protein found in some virus species. Alone, these 11 proteins are not sufficient to facilitate virus replication, and the virus must infect a host cell to co-opt host proteins and pathways for the successful generation of progeny virus. Therefore, intracellular signaling events reflect the balance of virus replication and antiviral host responses. Understanding the virus-host interactions facilitating this balance could provide novel targets for disease intervention strategies.

RNA interference (RNAi) is an evolutionary conserved endogenous pathway to regulate gene expression in eukaryotes, and has been widely employed to study the impact of post-transcriptional gene silencing on biological processes. Meta-analysis of five recent RNAi-based studies that identified host genes important for influenza virus infection and replication [12]–[16], identified common pathways associated with steps in the virus life cycle [13], [16]–[19]. Despite differences in methodology and reagents used in these studies, a level of accord was evident among the host cell pathways affected. Overlap was identified in pathways used for virus entry [16]–[19], fusion of the endosomal and viral membrane [13], [17]–[19], transport of the viral components to the nucleus [14], [15], as well as late events including export of the vRNP complex and RNA into the cytoplasm [12], [13], [16]–[18].

Viral infection triggers host responses that engage signaling networks which have a fundamental role in the anti-viral response. Previous studies have identified human protein kinases (HPKs) having key functions in influenza biology. By example these include protein kinase C (PKC) which is induced by viral binding to cell surface [20], [21], the extracellular signal-regulated kinase ERK [22], [23] induced by accumulation of viral HA on the cell surface via PKC and regulating RNP export [24], and phosphatidylinositol-3 kinase (PI3K) [24]. The inhibition of this signaling network results in nuclear retention of the vRNP and decrease in influenza virus replication [22], [25]. NF- κβ, a key mediator, is induced by accumulation of viral HA, NP and M1 proteins [25]–[34]. Additionally, the presence of viral dsRNA has been shown to activate signaling cascades involving IKK–NF-κβ, c-Jun N-terminal kinase (JNK), and P38 mitogen-activated protein kinase (MAPK) cascades all which regulate the expression of antiviral cytokines [35]–[37]. Together, these findings show the importance of HPKs in influenza virus replication making them targets for disease intervention strategies.

Post-transcriptional gene silencing by small non-coding microRNAs (miRNAs) has been recognized as an important mechanism of regulating host gene activity and is mediated via interaction between the miRNA seed site (nt 2–8 on the guide miRNA strand) and the 3′UTR of target gene(s) [38]–[40] to cause transcript decay or translational block. Owing to the short sequence complementarity between the miRNA “seed site” and the gene 3′UTR, >30κβhuman transcriptome is believed to be under miRNA regulation. It is thought that miRNAs have evolved to preferentially target and regulate key signaling nodes within a signal transduction network [41], [42]. Though effects of miRNA per gene may be subtle, targeting multiple cellular pathways and targets lead to observable biological phenotypes. miRNAs can be considered molecular rheostats of gene function in contrast to siRNAs which catalyze significant reductions in gene mRNA levels. [39]. Antiviral signaling pathways, and the HPKs involved, are leading candidates for miRNA regulation due to dose-sensitivity and the fine-tuning nature of miRNAs [43]. MiRNA expression is modified in response to a variety of cell stimuli including virus infection, and studies have shown that viruses exploit miRNA deregulation for their own benefit. For example, miR-132 has been shown to be highly induced after herpes simplex virus-1 (HSV-1), and human cytomegalovirus (HCMV) infection, and to down-regulate the expression of interferon-stimulated genes thereby facilitating virus replication [44]. HSV-1 replication is suppressed when miRNA-101 (miR-101) targets a subunit of mitochondrial ATP synthase (ATP5B) [45]. Human immunodeficiency virus type 1 (HIV-1) down-regulates the expression of many cellular miRNAs [46], and for miR-17/92, miRNA suppression is required for efficient virus replication [47]. Influenza virus infection modulates multiple cellular miRNAs, and miR-323, miR-491, and miR-654 have been shown to inhibit viral replication by binding to the viral PB1 gene [48], while miR-507 and miR-136 have potential binding sites within the viral PB2 and HA genes [49]. Additionally, miR-26a and miR-939 regulate the replication of H1N1 influenza virus in MDCK cells [48]. These data show that miRNAs are important for governing aspects of the host response to influenza virus replication. While all these studies have identified patterns of miRNA deregulation following viral infection, pathways and genes regulated by these deregulated miRNAs remain largely unexplored.

The focus of this study was to identify kinases essential for influenza virus replication, and to determine miRNAs that regulate their expression. To this end, genetic screening was performed using siRNAs to identify and validate kinase genes required for infection or replication of A/WSN/33 influenza. Preliminary hits from the primary screen were independently validated using siRNAs targeting a novel seed site on the same gene, and multiple endpoint assays that included PCR to measure viral genome replication, TCID_50_ to measure virus replication in MDCK cells, and high-content analysis to quantitate intracellular levels and location of nucleoprotein, NP, staining. Eighteen HPKs were confirmed of which 3 were anti-viral and 15 pro-viral. Six of 18 HPKs modulated A/WSN/33 influenza virus replication as well as A/New Caledonia/20/99 virus as validated using the same endpoint assays. Computationally predicted miRNAs targeting the six HPKs were compared to miRNAs deregulated during influenza infection [50]–[54] to shortlist miRNAs for validation. Native miRNA activity of these miRNAs was inhibited or up-regulated using miRNA inhibitors or mimics, respectively, and their impact on gene expression and viral replication was analyzed using qRT-PCR and high throughput microscopy based screening. These studies showed that influenza deregulated multiple miRNAs involved in regulating HPKs found to be important for influenza replication. Together, these analyses identify novel miRNA-HPK interactions involved in influenza virus replication, and help to unravel principles of the virus-host interface.

## Materials and Methods

### Cell Culture and Viruses

To minimize biological variation, a single passage of A549 human lung epithelial cells (CCL-185, ATCC) and Madin–Darby canine kidney cells (MDCK, CCL-34, ATCC) were used for all assays from a frozen stock stored in 10% DMSO and 90% fetal bovine serum (FBS), in liquid nitrogen. The cell lines were cultured in Dulbecco’s modified Eagle’s medium (DMEM, HyClone, Thermo Scientific) supplemented with 5% heat-inactivated FBS (HyClone, Thermo Scientific) at 37°C and 5% CO_2_. All cells were confirmed to be free of mycoplasma using a PlasmoTest kit (InvivoGen, San Diego, CA).

Influenza A/WSN/33 (H1N1) virus and A/New Caledonia/20/99 (H1N1) were grown in the allantoic cavities of 9-day-old embryonated chicken eggs as previously described [55]. Virus stocks were titrated in MDCK cells as previously described [56] and a 50% tissue culture infectious dose (TCID_50_) was determined using the method described by Reed and Muench [57].

### siRNAs and Transfection Assay

The siGENOME library is shipped as a series of 96 well plates with 0.5 nmol of lyophilized siRNA per well. The HPK library contains 9 master plates (a total of 720 gene targets). A siRNA arrayed library containing four pooled siRNAs per target gene for 720 different human protein kinase genes (Dharmacon siARRAY siRNA Library (G-003505 Human Protein Kinase Lot 08105), Thermo Scientific) was used for the primary siRNA screen. Controls included a siRNA targeting mitogen-activated protein kinase kinase 1 positive control (siMEK, 5′-GCACAUGGAUGGAGGUUCU-3′, 5′-GCAGAGAGAGCAGAUUUGA-3′, 5′-GAGCAGAUUUGAAGCAACU-3′, 5′-CCAGAAAGCUAAUUCAUCU-3′, siGENOME smartpool, Dharmacon M-003571-01), a negative non-targeting control siRNA (siNEG, 5′-UAGCGACUAAACACAUCAA-3′, siCONTROL Non-Targeting siRNA #1, Dharmacon D-001210-01-05), and a control for cellular cytotoxicity (TOX, Dharmacon D-001500-01-05). Each SMARTpool siRNA reagent used in the primary screen was validated for on-target activity by Dharmacon Thermo Fisher and consists of four rationally designed siRNAs targeting a distinct region of the target mRNA to achieve gene knockdown and reduce the incidence of off target effects (Table S1). To assure maximum silencing of target gene expression in our system, transfection and detection conditions were first optimized using the validated SMARTpool siMEK positive control (Figure S2A). The sequences for all siRNA duplexes provided with Dharmacon’s siGENOME libraries are proprietary and confidential and could not be listed. A list of the 720 HPK targeted by the library are listed in Table S1, as well as the siRNA sequences used for validation screen. All siRNAs were resuspended in Dharmacon siRNA buffer to a concentration of 1 µM and stored at −80°C.

All siRNAs were reverse transfected into A549 cells at a 50 nM final concentration using 0.4% Dharmafect 1 (Dharmacon) and incubated at 37°C and 5% CO_2_ for 48 h. Cell cytotoxicity was evaluated in all siRNA transfected cells compared to the TOX positive control and non-target negative control number. A “percent cytotoxicity” of the controls is determined for each experimental siRNA and cytotoxicity was determined to be >20% based on the bioluminescent measurement of adenylate kinase (ToxiLight BioAssay Kit, Lonza) (Table S1). A SafireX_2_ luminometer (Tecan U.S., Durham, NC) was used for the luminescence readout. All RNA interference (RNAi) experiments were carried out according to the Minimum Information for an RNAi Experiments (MIARE) guidelines [58].

### Infection Assays

The infection assay involved 1 h incubation at 37°C with either A/WSN/33 or A/New Caledonia/20/99 at the indicated multiplicity of infection (MOI). After 1 h incubation at 37°C, cells were rinsed with PBS and replenished with fresh media without virus. Cells used to study A/WSN/33 were cultured and infected in DMEM supplemented with 5% FBS and cells used to study A/New Caledonia/20/99 in DMEM supplemented with 0.2% bovine serum albumin (Sigma Aldrich) and 1 µg/mL of TPCK trypsin (Worthington).

Supernatants were harvested 48 h after infection and the viral titers were determined by TCID_50_ [59] or plaque assay [57] on MDCK cells. For TCID_50_, 2×10^4^ MDCK cells per well in 100 µl were plated in a 96-well flat-bottom cell culture plate. The virus samples were 1/10 serially diluted and each diluted sample was used to infect the replicate wells by incubating 50 µl of the sample on a confluent monolayer of MDCK cells at 37°C and 5% CO_2._ At 72 h, the supernatants from all wells were transferred to a V-bottom plate. Chicken red blood cells (50 µl at 0.5%) were added and incubated for 1 h at 4°C. The agglutination wells were counted and used to determine TCID_50_ values [57], [60]. Plaque assays were performed with 1/10 serial dilutions of the virus samples on a confluent monolayer of MDCK cells, overlaid with Avicel containing either 5% FBS or 1 µg/ml TPCK-trypsin [56]. Cells were incubated for 72 h and then plaques were visualized by staining with 0.1% crystal violet.

### Normalization and Z-score Analysis

The primary screen data was normalized by correcting the raw data for across plate variation. Specifically, the percent inhibition of infectious virus was calculated for each experimental siRNA such that the difference of the experimental HPK siRNA treated well (X_siHPK_) was subtracted from the mean of the negative control well values (_siNEG_) and then divided by the difference of the means of the negative control and the TOX control (_siTOX_) for each plate:
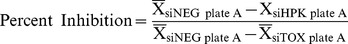



The primary screen was performed in at least two independent experiments, and each experiment was performed in duplicate, yielding a dataset of ≥4 replicates for each X_siHPK_. The mean of the replicates for each of the 720 X_siHPK_ was calculated and the resulting dataset was standardized using Z-score analysis (Table S1), whereby the mean (µ) of the data becomes zero and the standard deviation (SD) becomes 1. A “positive hit” in the primary screen was considered to have a 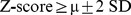 using the formula below.
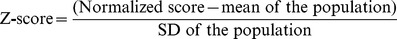



### Quantitative Real-time PCR

The RNA from A549 cells was isolated using an RNeasy Mini Kit (Qiagen) according to the manufacturer’s instructions. The quantity of total RNA was determined using a NanoDrop ND-1000 Fluorospectrometer (NanoDrop Technologies, Wilmington, DE). Equal amounts of RNA were then reverse transcribed to cDNA using random hexamers and MuLV reverse transcriptase (High-Capacity cDNA Reverse Transcription Kit, Applied Biosystems) in accordance to the manufacturer’s protocol.

For quantification of influenza M gene expression, qPCR was performed using 200 nM internal probe (M +64, 5′-FAM-TCA GGC CCC CTC AAA GCC GA-BHQ-1-3′), 400 nM forward primer (M +25, 5′-AGATGAGTCTTCTAACCGAGGTCG-3′), and 400 nM reverse primer (M-124, 5′-TGCAAAAACATCTTCAAGTCTCTG-3′) following a previously described TaqMan assay [61]. The cycling conditions for qPCR were 95°C for 10 min followed by 40 cycles of 95°C for 15 sec, 60°C for 30 sec, and 72°C for 15 sec. The qPCR was carried out and analyzed with a Stratagene Mx3005P instrument and software (La Jolla, CA). Copy numbers were determined by generation of a standard curve using plasmid DNA encoding influenza M gene [61]. Results reported for these studies were the averages of at least three replicates.

To determine the gene silencing efficiency associated with siRNA treatment, qPCR was performed using QuantiTect SYBR Green PCR Master Mix (Qiagen) according to the manufacturer’s instructions with the primer sequences described in Table S4. Relative expression level was calculated using the endogenous control glyceraldehyde 3-phosphate dehydrogenase (GAPDH). Fold changes were calculated against the mean of negative control siRNA treated cells. Methodology and data analysis for all qPCR experiments were carried out according to the Minimum Information for Publication of Quantitative Real-Time PCR Experiments (MIQE) guidelines [62].

### Indirect Immunofluorescence to Detect Influenza NP

A549 cells were fixed with 3.7% formaldehyde and permeabilized with 0.5% Triton X-100 (Sigma), 10% FBS (Hyclone) in PBS. A549 cells were incubated with a primary antibody against the viral NP (25 µg/mL, H16-L10, HB65; ATCC) diluted in PBS with 10% FBS for 1 h at room temperature. Following this, the cells were incubated with Alexa-488 goat anti-mouse secondary antibody (A11001, Invitrogen) diluted 1∶500 in PBS with 10% FBS for 1 h in the dark, and the cells were subsequently stained for 20 min with 1 µg/mL of 4′-6-Diamidino-2-phenylindole stain (DAPI, Invitrogen). The cells were imaged using the EVOS digital inverted fluorescent microscope (Advanced Microscopy Group, Bothell, WA) at two wavelengths, 488 nm to detect Influenza infected cells expressing NP, and 350 nm for nuclear DNA bound by DAPI. For quantification of NP immunofluorescence, cells were fixed, permeabilized and stained as above and 20× images were acquired and analyzed using Cellomics ArrayScan VTI High Content Imager and Cellomics ArrayScan software (Thermo Fisher Scientific).

### Indirect Immunofluorescence to Detect HPK Expression

A549 cells in 96 well plates were transfected with HPK specific miRNA inhibitors or mimics and incubated for 48 h. Cells were fixed with 4% formaldehyde in PBS and permeabilized with 0.5% TritonX-100 in PBS/20 min/RT. Cells were incubated with biotinylated antibodies against HPKs (10 ug/ml) diluted in PBS+5% BSA for 2 h at room temperature or 4°C overnight, washed with PBS (10 min×3) and incubated with Streptavidin-Alexa 488 conjugated secondary antibodies for 3 hrs. Excess secondary antibody was removed by washing with PBS thrice (10 min each wash) and nuclei were finally stained with 4′-6-Diamidino-2-phenylindole stain (DAPI, Invitrogen) for 10 min at RT. Plates were scanned with Cellomics ArrayScan VTI scanner using Target Acquisition protocol at 20× magnification. DAPI fluorescence was measured in channel 1 and Alexa 488 fluorescence was measured in channel 2. A minimum of 5000 valid channel 2 objects were counted per well in triplicate for each mimic/inhibitor treatment. Data was exported into csv files and statistically analyzed using GraphPad Prism version 5.0.

### miRNA Target Prediction and Literature Analysis

miRNAs targeting the selected HPKs were mined using miRWalk [63]. Briefly, miRWalk tabulates the miRNAs targeting a gene of interest based on predictions from multiple algorithms and can be used to either find miRNAs targeting a gene(s) or vice versa. Lists were exported into spreadsheets containing data on miRNA deregulation during influenza infection and compared. Most predictions with significant scores have (1) a significant seed region match and (2) are evolutionarily conserved as per TargetScan [64].

### miRNA Validation

A549 cells (2×10^4^) were transfected with either a miRIDIAN miRNA antagonist (25 nM), a miRIDIAN miRNA agonist (12.5 nM) (Dharmacon), or a siRNA targeting the HPK under study using Lipofectamine 2000 (Invitrogen) per the manufacturer’s protocol. miRNA let-7f (expressed at ∼750 copies in A549 cell [65]) was used to optimize inhibitor or mimic concentrations, and at 25 nM miRNA antagonist concentration, generally reduced miRNA expression by >85%, while transfection with 25 nM miRNA agonists led to an increase in miRNA levels (Figure S4). The miRIDIAN miRNA antagonists are chemically modified dsRNAs consisting of a central region complementary to the mature miRNA flanked by 0-16 nt long sequences with reverse complement to pri-miRNA to enhance specificity and reduce RISC incorporation [66]–[68]. Conversely, agonists are chemically modified dsRNAs that increased the guide strand concentration in RISC complex, thus increasing native miRNA mediated repression [68], [69]. Cells treated with an equal concentration of a non-targeting control sequence (mock) were used to control for non-sequence-specific effects in miRNA experiments. After 48 h post-transfection, the cells were infected (MOI = 0.01) with A/WSN/33 for 48 h. Virus replication and HPK knockdown was assayed by high content microscopy and HPK expression was analyzed using qPCR (for transcript) and high content screening (for protein). For qPCR, 18S rRNA was used as housekeeping control. Fold changes were calculated against the mean of mock treated cells.

### Statistical Analysis

Statistical analysis for validation screen, pathway analysis, and miRNA studies were performed using Student’s t test with the GraphPad Prism 5 software. Statistical significance (p<0.05) is indicated by a single asterisks or double asterisks if highly significant (p<0.001).

## Results

### RNAi Screen Identifies Host Kinase Genes Important for Influenza Virus Replication

**Figure 1:**
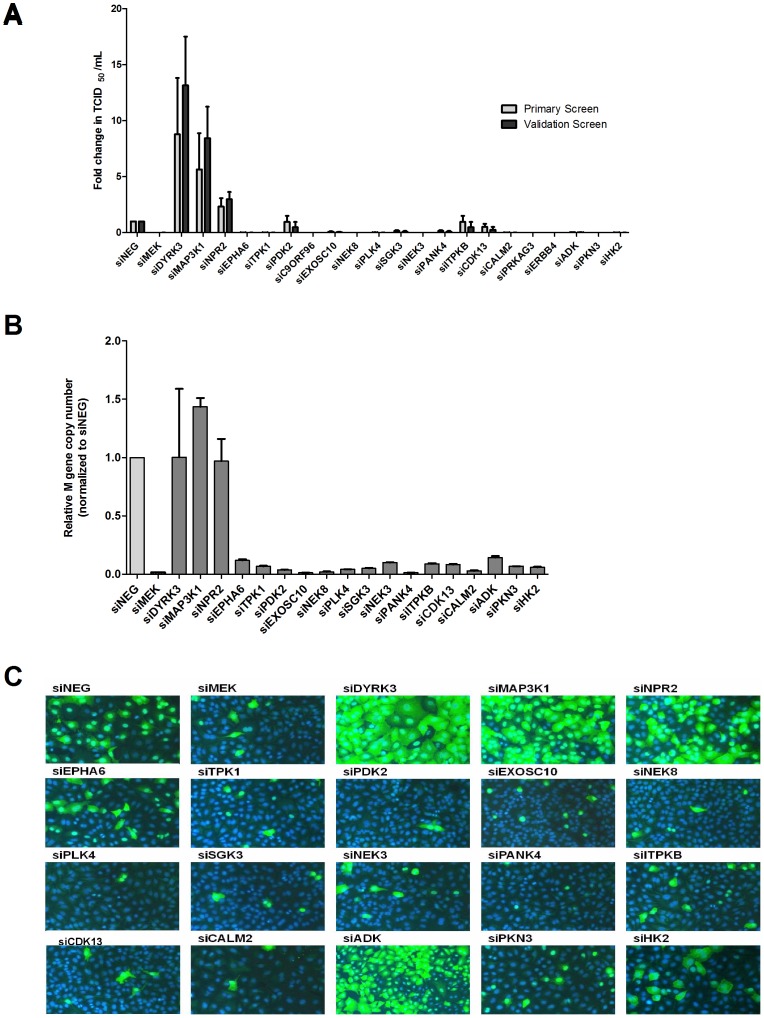
Validation of human protein kinase genes affecting influenza virus replication. A549 cells were reverse transfected with 50 nM of a non-target negative control siRNA (siNEG) or with siHPK and infected at a MOI = 0.01 with A/WSN/33. (A) 72 hours after infection, supernatants were harvested and the viral titers were determined by TCID_50_ assay on MDCK cells (B) After a 48 hour infection, the effect of siRNA silencing on influenza virus replication was measured by quantifying the levels of influenza M gene expression. The RNA from siRNA-transfected and WSN-infected A549s was isolated and used for quantification with an influenza M-specific primer/probe set. Light gray bars indicate controls. Data show mean±SEM from 3 independent experiments. (C) After a 48 hour infection, cells were fixed, permeabilized and incubated with an anti-influenza virus NP monoclonal antibody and subsequently with an Alexa-488 goat anti-mouse secondary antibody and DAPI. The intracellular distribution of the viral RNPs (NP, green) and cellular nuclei (DAPI, blue) are shown.

HPKs are key mediators of signaling events in a cell and inhibition of HPKs has been shown to reduce progeny virus production both *in vitro* and *in vivo* [70]. To better understand the contribution of HPKs during influenza virus replication, an RNAi-based genetic screen was performed and the hits validated (Figure S1). siRNA SMARTpools individually targeting 720 different HPKs were transfected into A549 cells followed by influenza A/WSN/33 infection. Levels of infectious virus were determined by TCID_50_ and the effects of siRNA treatment quantified using a z-score method where a positive z-score (>2.0) indicates HPK knockdown resulting in an increase in viral replication and a negative z score (<−2.0) value indicates a resulting decrease in virus replication. Using the 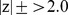 identified 22 of the 720 HPK genes as modulators of influenza A/WSN/33 replication (Table 1; Figure S2A+B) of which 3 (NPR, MAP3K1, DYRK3) increased influenza virus replication and were designated as anti-viral HPKs, while silencing 19/22 genes (EPHA6, TPK1, PDK2, C9ORF96, EXOSC10, NEK8, PLK4, SGK3, NEK3, PANK4, ITPKB, CDC2L5/CDK13, CDK3, CALM2, PRKAG3, ERBB4, ADK, PKN3, HK2) decreased influenza virus replication compared to siNEG transfected cells and were designated pro-viral HPKs (Table 1, Figure 1A, Figure S2B). Cells transfected with the positive control, i.e. siMEK, had consistently lower influenza virus titers compared to siNEG transfected cells (Table S1).

**Table 1:** Human kinase genes identified important for influenza virus replication in primary screen.

Symbol	Name	Z-score	Log_10_ TCID_50_/mL
NPR2	natriuretic peptide receptor B/guanylate cyclase B	2.64	6.5
MAP3K1	mitogen-activated protein kinase kinase kinase 1	2.55	5.7
DYRK3	dual-specificity tyrosine-(Y)-phosphorylation regulated kinase 3	2.23	5.9
EPHA6	EPH receptor A6	−2.01	3.0
TPK1	thiamin pyrophosphokinase 1	−2.01	3.0
PDK2	pyruvate dehydrogenase kinase, isozyme 2	−2.03	2.7
C9ORF96	chromosome 9 open reading frame 96	−2.16	2.7
EXOSC10	exosome component 10	−2.16	2.7
NEK8*	never in mitosis gene a- related kinase 8	−2.18	2.7
PLK4*	polo-like kinase 4	−2.18	1.7
SGK3	serum/glucocorticoid regulated kinase family, member 3	−2.19	2.7
NEK3	never in mitosis gene a-related kinase 3	−2.22	2.7
PANK4*	pantothenate kinase 4	−2.35	1.7
ITPKB*	inositol 1,4,5-trisphosphate 3-kinase B	−2.35	1.7
CDC2L5	cell division cycle 2-like 5	−2.38	1.7
CDK3	cyclin-dependent kinase 3	−2.40	1.7
CALM2*	calmodulin 2	−2.59	n.v.
PRKAG3	protein kinase, AMP-activated, gamma 3 non-catalytic subunit	−2.59	n.v.
ERBB4	v-erb-a erythroblastic leukemia viral oncogene homolog 4	−2.59	n.v.
ADK	adenosine kinase	−2.59	n.v.
PKN3	protein kinase N3	−2.59	n.v.
HK2*	hexokinase 2	−2.59	n.v.

* Genes identified important for influenza in a previous screen; n.v., no detectable virus.

List of HPKs with Z scores ±>2.0 identified in primary screen. Positive z-scores indicated anti-viral HPKs while negative z-scores indicate pro-viral HPKs.

To rule out false positive/negative hits due to potential siRNA off-target effects [71], primary hits were retested using a novel synthetic siRNA targeting the same gene but at a different seed site (Table S2), and HPKs that exhibited identical phenotypes as the primary screen were thus validated. Transfection of siRNAs targeting NPR2, **MAP3K1, DYRK3,** EPHA6, TPK1, PDK2, EXOSC10, **NEK8, PLK4**, **SGK3,** NEK3, PANK4, ITPKB, **CDK13,** CALM2, ADK, PKN3, and HK2 followed by gene specific qPCR demonstrated >80% silencing of the target mRNA (Figure S3). The novel siRNAs targeting genes CDK3, PRKAG3, ERBB4, and C9ORF96 were unable to knockdown mRNA expression and were excluded from the validation screen. Transfection of A549 cells with the novel siRNAs for the 18 HPKs followed by A/WSN/33 influenza infection modulated influenza replication significantly (p<0.01) similar to those obtained in the primary screen confirming the relevance of the HPK genes (Figure 1A). These findings were substantiated with additional endpoint assays that included measurement of viral genome replication by qPCR (Figure 1B), and influenza nucleoprotein determined by high content analysis (Figure 1C). Thus, 17 of 18 HPK hits repeated the original screen phenotype, i.e. increased or decreased virus replication. Identifying 22 hits out of 720 in the primary screen yielded a positive hit rate of 3.1% which is consistent with other related screens [12]–[16]. The majority of the hits were novel; however, PANK4, NEK8, ITPKB, CALM2 and HK2 have been identified in other influenza screens (Table 1; Table S3) [12]–[16]. A meta-analysis of the validated HPK hits combined with those previously identified revealed that although specific genes are not consistently identified, many genes are in shared cell pathways important for influenza including PI3K/AKT signaling, NFKB, PKC/CA++ signaling, and p53/DNA damage pathways [72].

For the hit selection, it is important to identify false-negatives in which siRNAs with larger effects are not selected, and false-positives in which siRNAs with negligible effects are selected as hits. Assessment of the false-negative rate in these studies using a z- score ≥ µ ±2 SD revealed a rate of 87.5%. If the potential hits are expanded to those fitting a z-score ≥ µ ±1SD, the false-negative rate decreases to 23% because 37 HPK genes can be identified that overlap with HPK genes discovered in related published screens [12]–[16]. Since only the ADK gene was not validated, the false-positive rate in the primary screen was 4.5% (1 out of 22). This good false-positive rate is linked to the assay conditions, i.e. using pooled siRNA duplexes reported to reduce the rate of false-positives [73].

### Validated HPK Genes Affect Replication of Different Influenza Strains

**Figure 2:**
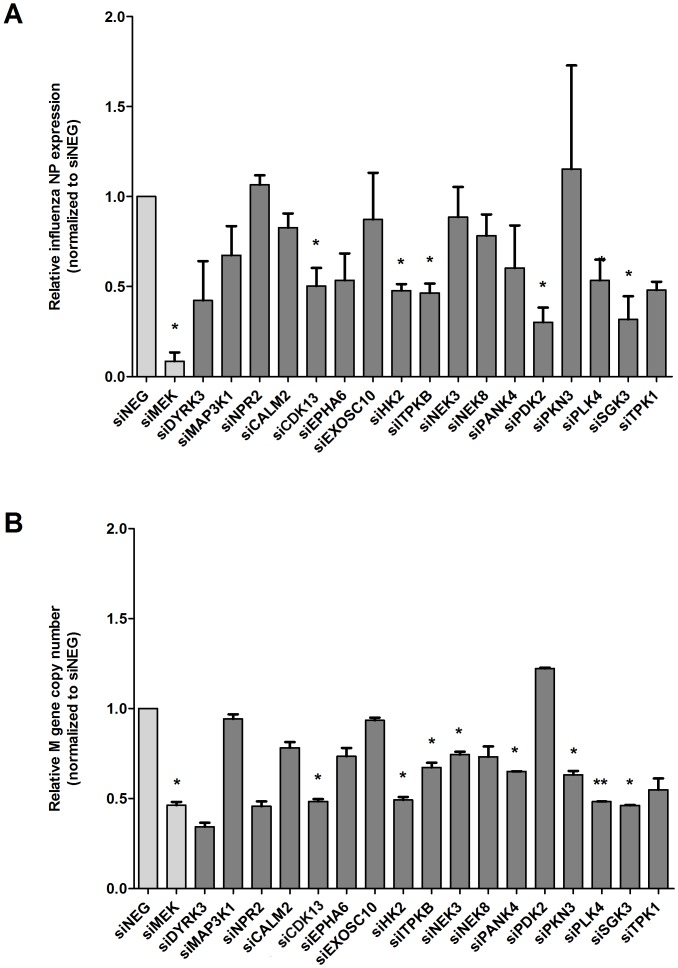
Human protein kinase genes affect H1N1 A/New Caledonia/20/99 virus replication. A549 cells were reverse transfected with 50 nM of siRNA specific for validated HPK genes and after 48 h the cells were infected with A/New Caledonia/20/99 at an MOI of 0.01 in the presence of 1 µg/ml TPCK-trypsin. After 48 h of infection, RNA was extracted and the effect of siRNA silencing of HPK genes on viral genome replication was measured by quantifying (A) influenza NP expression and (B) the level of influenza M gene. Data show mean±SEM of 3 independent experiments. *p<0.05 and **p<0.001 compared to control.

Influenza virus A/WSN/33 was chosen for the primary and validation screens based on its ability to replicate without exogenous trypsin. However, because it is a lab-adapted strain, another influenza virus strain was tested to validate the HPK hits important for influenza virus replication. Therefore, A549 cells were transfected with siRNAs targeting the hit HPKs and subsequently infected with influenza A/New Caledonia/20/99 (Figure 2). Of the 17 HPK genes identified important for A/WSN/33 replication, six (CDK13, HK2, NEK8, PANK4, PLK4, SGK3) emulated the phenotype following A/New Caledonia/20/99 infection, i.e. increased or decreased virus replication as measured by influenza NP localization (Figure 2A) and influenza M gene levels (Figure 2B). In addition, four of the six (HK2, NEK8, PANK4, PLK4) have also been identified important for influenza virus replication in other influenza-genome screens (Table 1; Table S3) [13], [15], [16].

### Systematic Analysis of miRNAs Predicted to Govern HPK Genes

Deregulation of cell cycle pathways is a hallmark of influenza virus infection and replication [74]–[76]. Meta-analysis of microarray data from individuals with mild or severe influenza infections identified cell cycle control and apoptosis as the major pathways kinked to severity and outcome of influenza infection [75]. Four of the 6 validated HPKs, i.e. CDK13, NEK8, PLK4 and SGK3, have roles in cell cycle regulation. Similarly, two of 3 anti-viral HPKs (MAP3K1, DYRK3) are also implicated in regulation of cell cycle. Specifically, CDK13 has been shown to interact with L-type cyclins and regulate alternative splicing [77], as well as increase HIV mRNA splicing, and upon silencing leads to increased HIV replication via phosphorylation of serine/arginine-rich splicing factor 1 (SRSF1/ASF/SF2) [78]. NEK8 phosphorylates bicaudal D (Bicd2) [79], binds to centrosomes and regulates ciliogenesis [80]. PLK4 also regulates centriole duplication during cell cycle [81]–[83]. Repressing the autoregulation of PLK4 leads to centrosome amplification and increased p53 activity [84]. PLK4 activity is crucial for organization of the centrosome, the main microtubule organizing center (MTOC) in the cell that regulates not only cell division, but also movement of intracellular organelles such as late endosomes and lysosomes. Organization of the MTOC is important for influenza virus infection and replication since RNAi depletion of genes such as Histone deacetylase 8 (HDAC8) that are involved in MTOC organization led to reduced motility of early endosomes and lysosomes and misdistribution of intracellular vesicles and organelles [85]. SGK3 belongs to the three member family of serum glucocorticoid kinases (SGK1, 2 and 3), and has been shown to regulate influenza vRNP nuclear export into the cytoplasm [86]. SGK3 has been implicated in regulating cell survival [87]. MAP3K1 is a multifunctional protein and important for induction of IFN-β induction in response to poly I: C challenge via IRF-3 activation [88]. MAP3K1 also inhibits expansion of virus specific CD8+ T cells [89]. DYRK3 belongs to a family of dual specificity tyrosine kinases that activate by auto phosphorylation and catalyze phosphorylation of histone H3 and H2B. DYRK3 phosphorylates and activates sirtuin 1 (SIRT1) turnover, causes deacetylation of p53 and increased apoptosis [90]. Influenza virus infection upregulates mTORC1 signaling pathway [91] and inhibition of mTORC1 can significantly delay mortality due lethal challenge of influenza virus in mice [92]. DYRK3 has been shown to stabilize P-granule like structures and the mTORC1 pathway during cellular stress. Inactivation of DYRK3 traps mTORC1 inside cytosolic stress granules while activation of DYRK3 promotes dissolution of stress granules and release of mTORC1 [93]. These observations show that the validated HPKs affect critical pathways during influenza infection and replication, thus miRNA regulation of these genes were examined during influenza infection.

**Figure 3:**
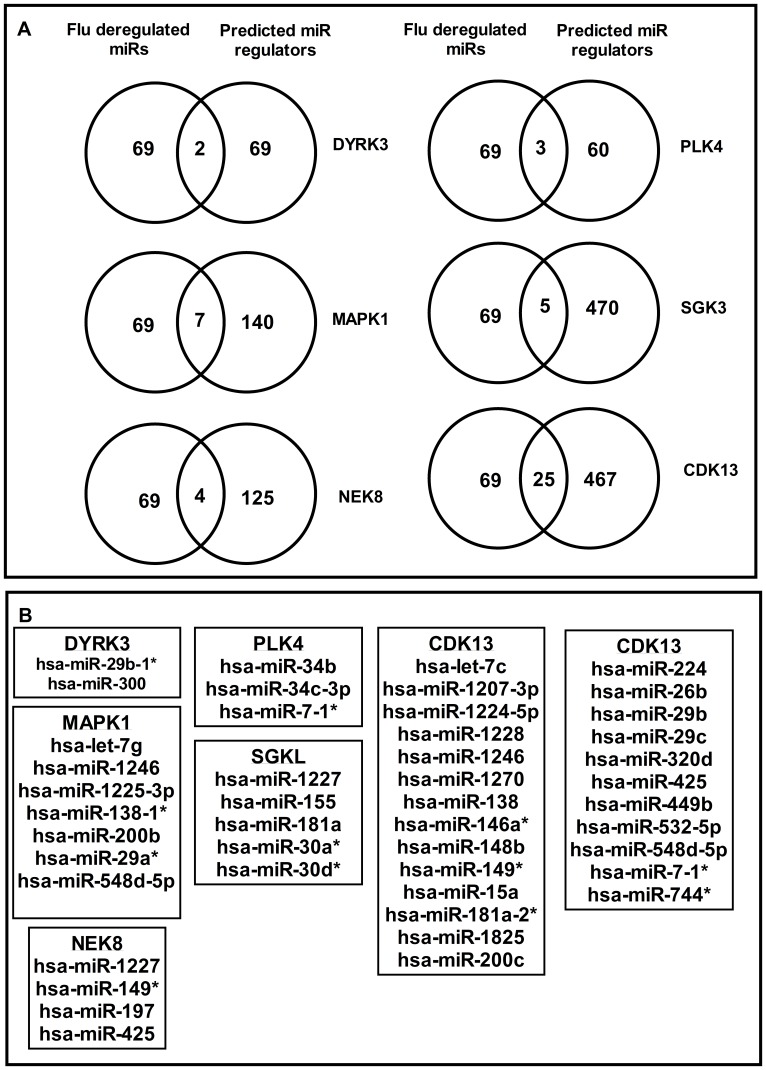
Identifying miRNA regulators of HPKs important for influenza replication. (A) Venn diagrams showing miRNAs common to computationally predicted miRNA regulators and influenza deregulated miRNAs. (B) miRNAs that are shared between computationally predicted HPK regulators and miRNAs deregulated during influenza infection.

**Figure 4:**
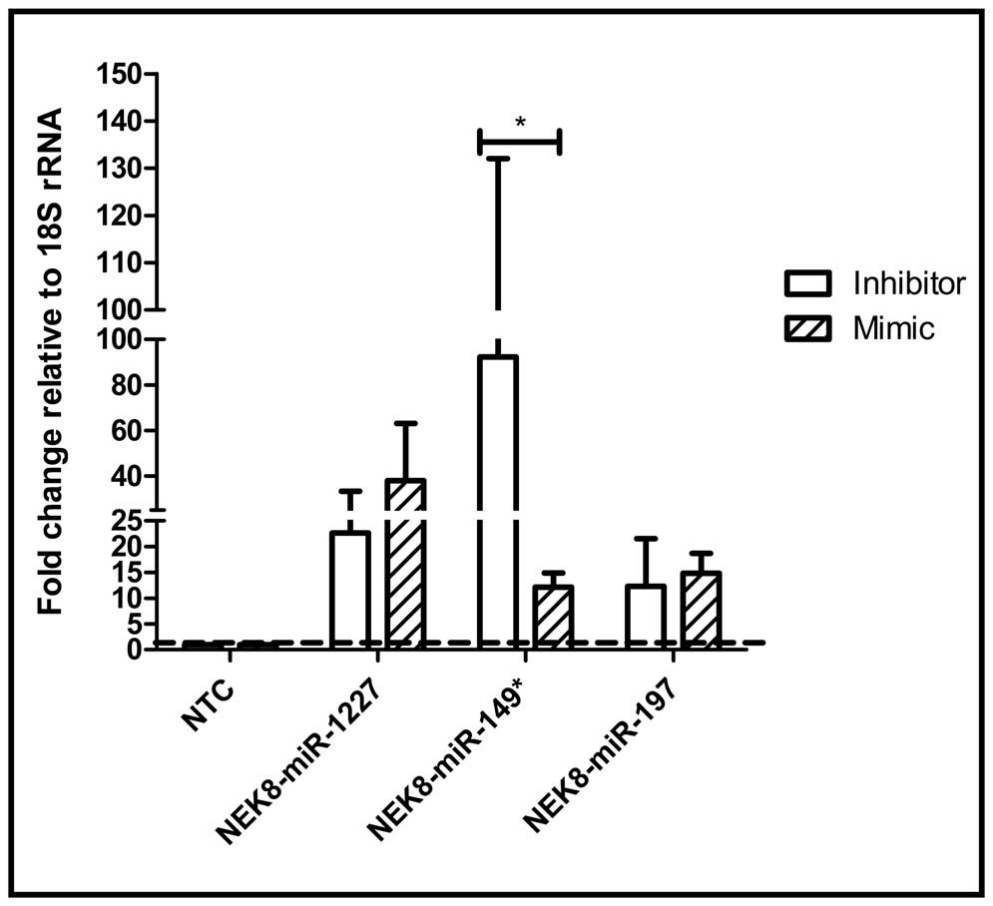
miRNA regulators of NEK8. A549 cells were transfected with 25 nM of miR-1227, -149* and -197 inhibitor/mimic for 48 hrs followed by RNA extraction and RT-qPCR with NEK8 specific primers. Expression data was normalized to 18S rRNA expression and shown as mean±SEM of independent experiments. *p<0.05 compared to control.

**Figure 5:**
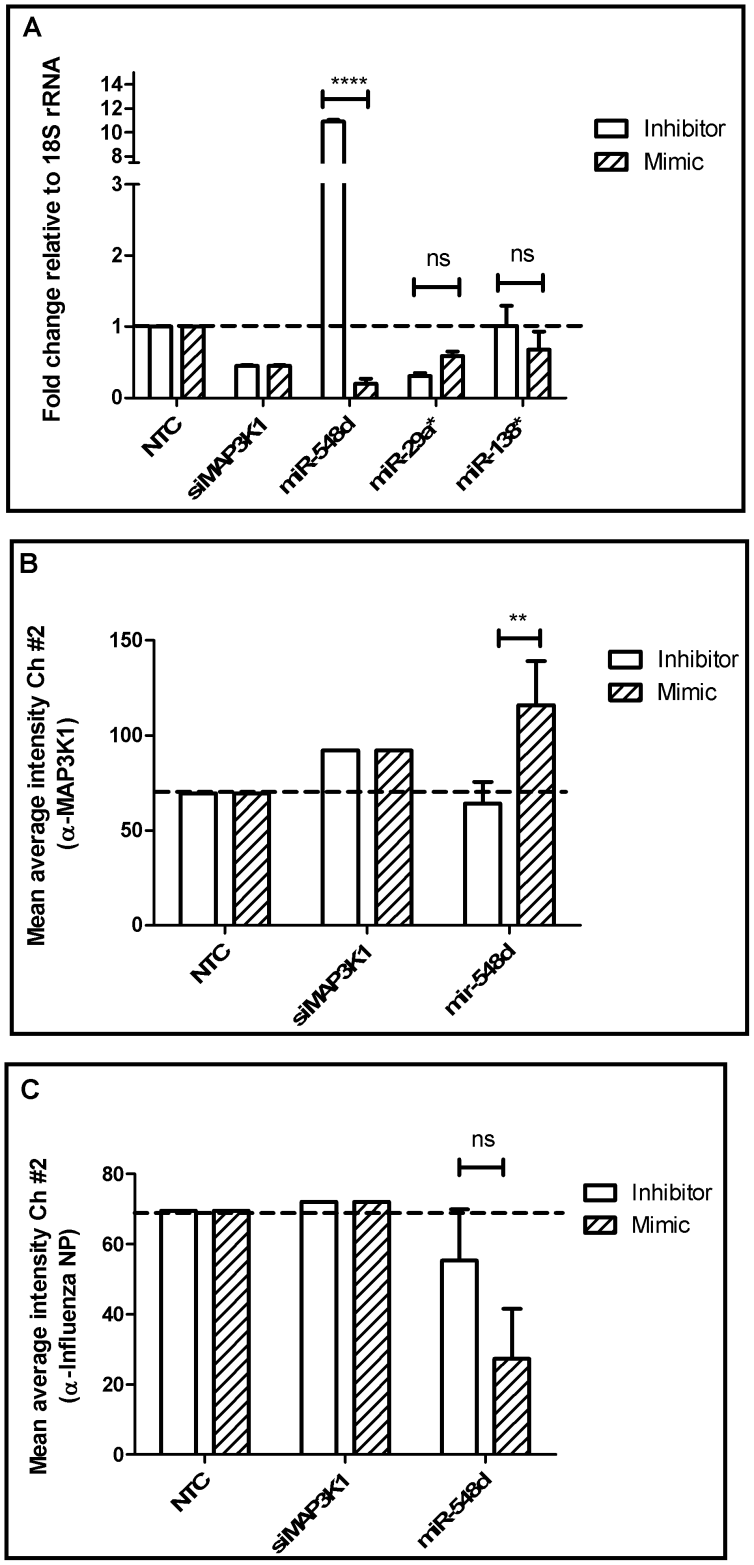
miRNA regulators of MAP3K1. (A) A549 cells were transfected with 25 nM of miR-548d, -29a and -138 inhibitor/mimic for 48 hrs followed by RNA extraction and RT-qPCR with MAP3K1 specific primers. Expression data was normalized to 18S rRNA expression and shown as mean±SEM of independent experiments. *p<0.05 compared to control. (B) A549 cells mock/transfected with miR-548d inhibitor/mimic for 48 hrs were fixed with 4% formaldehyde in PBS and stained for MAPK1 protein using biotinylated rabbit anti-MAP3K1 antibody (Abcam ab69533) and detected with Streptavidin-Alexa488. Cells were analyzed by Arrayscan Cellomics VTI scanner and data analyzed by GraphPad Prism. (C) A549 cells mock/transfected with miR-548d inhibitor/mimic for 48 hrs were infected with A/WSN/33 (MOI = 0.001) for 48 hrs and stained for influenza NP protein using mouse-anti-NP coupled to Alexa-488 and analyzed as above in (B). Data show mean±SEM of two independent experiments. *p<0.05.

miRNAs regulate multiple aspects of the host response to infection. While *in vivo* deregulation of host miRNA expression associated with influenza infection has been established, the pathways by which cellular miRNAs modulate host gene expression during influenza virus infection remain largely unexplored [94], [95]. Analysis of miRNA regulation of the HPKs was performed using existing data on miRNA and mRNA expression during influenza virus infection. qPCR assays for expression profiling of the HPKs (Figure S5) and analysis of gene expression omnibus (GEO) datasets GDS3919 [75], GDS3919 [75], GDS3595 [96] and GDS2762 [97] indicated that HPKs could be shortlisted to a variable extent based on those that are expressed in vitro and in vivo in mice during influenza infection and replication. To identify miRNAs that regulate these HPKs, a list of miRNAs deregulated during influenza infection (Table S5) was compared to computational predictions for NEK8, PLK4, SGK3 and CDK13, MAP3K1 and DYRK3 genes [98] (Figure 3A) providing a shortlist of miRNAs for experimental validation (Figure 3B). Details of miRNA seed match with target gene 3′UTR are given in Table S6. A panel of miRNA inhibitors and mimics that have been shown to consistently prevent or increase the incorporation of miRNA guide strand into the RISC complex [68] were used to modulate native miRNA activity. Previous studies have established that 25 nM of miRNA inhibitor reduces native miRNAs ≥85% in 24 h and is not cytotoxic (Figure S4) [99]. Thus, a miRNA concentration of 25 nM was used in all transfection assays. An important caveat of this assay is that while miRNA inhibitors are miRNA-specific and able to distinguish between different members of the same miRNA family, miRNA mimics can affect native levels of all members of a miRNA family especially when the seed sites are conserved. Based on the dogma of miRNA action and our own prior studies [99], [100], we expected a small but significant increase in target gene transcript/protein expression upon miRNA inhibition, and an opposite phenotype upon mimic supplementation. A549 cells were transfected with miRNA inhibitors or mimics, the cells assayed for cytotoxicity, and subsequently processed for HPK-specific qPCR to evaluate HPK gene expression, as well as gene-specific protein levels by anti-HPK antibodies. In parallel, similarly transfected A549 cells were infected with A/WSN/33 (MOI = 0.001) for 48 hrs, fixed and stained for influenza NP protein using an Alexa-488 coupled anti-NP antibody and analyzed using a high throughput Cellomics ArrayScan VTI microscope (Thermo Fisher). Data represent means of ≥ 5000 cells counted from at least 20 fields of triplicate wells for each treatment. Though all predicted miRNA HPK pairs were analyzed (Figure 3A and B), only data on miRNAs which impact HPK and/or virus replication is discussed hereafter. miRNAs targeting DYRK3, CDK13 and SGK3 did not alter HPK expression or viral replication and are not discussed further. Inhibition of miR-149* led to ∼10 fold NEK8 induction of transcript, but not protein, while miR-149* mimic transfection reduced NEK8 transcript expression below the level of the control (Figure 4). Though NEK8 transcript is significantly induced by 24 hrs post A/WSN/33 infection (Figure S5). NEK8 protein expression was not readily detected as the native level of NEK8 has low level expression even in mock and NTC transfected cells. This is due to rapid proteasome mediated degradation of NEK8 protein [80]. No effect of mR-149* modulation on influenza NP staining was evident suggesting that under the conditions of assay, NEK 8 transcript modulation by miR-149* did not have any effect on viral replication. Indeed, miR-149* induction during influenza infection has been reported to occur only post 72 hrs (Table A1 from [50]) and this could explain the lack of effect of miR-149* inhibition on NEK8 protein. MAP3K1 transcript expression was significantly up-regulated by miR-548d inhibitor treatment while the mimic down-regulated MAP3K1 transcript expression (Figure 5A). miR-29a or miR-138* treatments did not have any appreciable effect on MAP3K1 expression (data not shown). miR-548d inhibitor/mimic treatments did not alter MAP3K1 protein expression (Figure 5B). This could be attributable to off-targeting given the modest expression of miR-548d during H1N1 infection (Table SA5 from [50]), and the significant “seed identity” between the 68 members of the miR-548 family (Text S1). Alternatively, seed shifting [101] and/or the indirect effects of miR-548d on MAP3K1 via miR-548d regulation of IFN-λ1 may have contributed to the outcome [102]. Similar to the NEK8 findings, MAP3K1 transcript modulation by miR-548d did not alter viral replication.

**Figure 6:**
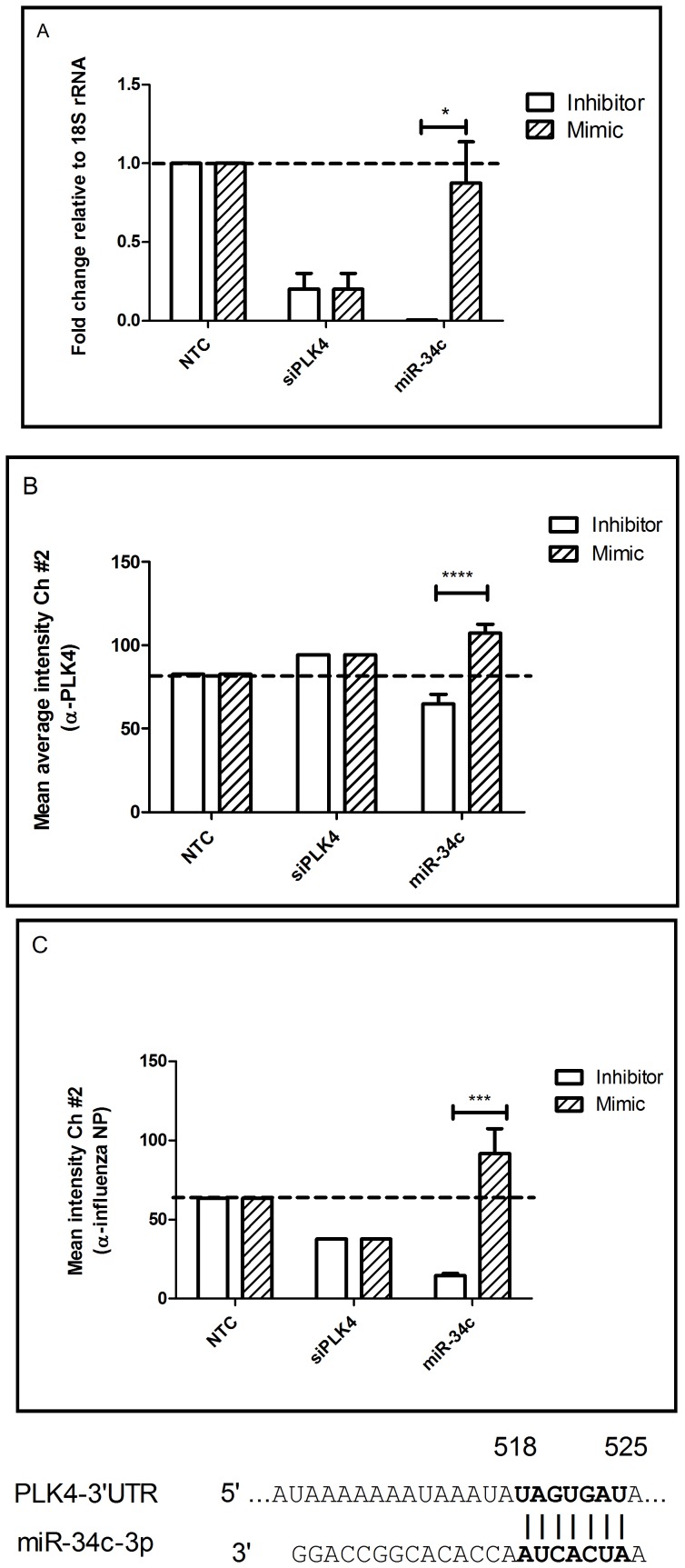
miRNA regulators of PLK4 (A) A549 cells were transfected with 25 nM of miR-34c inhibitor/mimic for 48 hrs followed by RNA extraction and RT-qPCR with PLK4 specific primers. Expression data was normalized to 18S rRNA expression and shown as mean±SEM of independent experiments. *p<0.05 compared to control. (B) A549 cells mock/transfected with miR-34c inhibitor/mimic for 48 hrs were fixed with 4% formaldehyde in PBS and stained for PLK4 protein using biotinylated rabbit anti-PLK4 antibody (Abcam ab71394) and detected with Streptavidin-Alexa488. Cells were analyzed by Arrayscan Cellomics VTI scanner and data analyzed by GraphPad Prism. (C) A549 cells mock/transfected with miR-34c inhibitor/mimic for 48 hrs were infected with A/WSN/33 (MOI = 0.001) for 48 hrs and stained for influenza NP protein using mouse-anti-NP coupled to Alexa-488 and analyzed as above in (B). Data show mean±SEM of two independent experiments. *p<0.05.

The most significant effects were observed for miR-34c and the PLK4 gene. While miR-34b and let-7i inhibitor/mimic treatments had no substantial effects on PLK4 transcript and protein expression (data not shown), miR-34c mimic considerably up-regulated PLK4 transcript (Figure 6A) and protein expression (Figure 6B), as well as influenza NP levels (Figure 6C). These observations are contrary to the dogma that miRNA inhibitor treatment should induce target gene expression, while mimic treatment should repress target gene expression. We believe these observations tie in well with known deregulation of cell cycle during influenza virus replication. Influenza infection has been shown to substantially alter the activity of p53, a master regulator of cell cycle via the NS1 protein [103]–[105]. Influenza NS1 associates with p53 [105] and RhoA protein [74] to regulate the host anti-viral response [103] causing cell cycle arrest in G0/G1 phase which is conducive to influenza replication. MiR-34c (and also miR-149*) expression is driven by p53 activation during influenza infection [104] to negatively regulate the activity of the transcription factor, Myc, which regulates S phase progression and DNA replication [106], [107]. Inhibition of miR-34c accelerates S phase, promotes excessive DNA synthesis, and affects the Myc-regulated gene, Bcl2, which is important for cell cycle control. In contrast, the MiR-34c mimic affects Myc activity and arrests the cell cycle in S phase [106]. During cell cycle, the PLK4 transcript and active phosphorylated PLK4 kinase are detected at S phase, peak during M phase, and are subsequently degraded by proteasome-mediated protein decay [82], [83]. miR-34c arrest of S phase cell cycle would prevent degradation of PLK4 transcript and protein, and thus help to explain the observation of increased PLK4 transcript and protein following miR-34c mimic treatment. Additionally, members of the miR-34 family, e.g. miR-34a/b-5, have been shown to enhance translation of neuronal tissue-specific polyadenylated transcript of β-actin [107]. Thus, a miR-34c mimic transfection can cause S phase cell cycle arrest and promote translation of target transcripts. Cells arrested in S phase would not be able to complete centriole duplication and accumulate increased PLK4 transcript and protein as was observed in this study. Previous studies demonstrated that cells arrested in G0/G1 or S phase are more conducive to influenza virus replication relative to mock treated cells, or cells in G2/M phase and our observations thus provide mechanistic insight into these findings [76]. Thus, we hypothesize that during influenza virus infection, NS1 mediated p53 up-regulation triggers miR-34c activity to regulate cell cycle through Myc, PLK4 and NEK8 (Figure S6). While miR-34c is believed to primarily target c-Myc [108], and the findings from this study show that miR-34c positively regulates PLK4, other miR-34c targets cannot be discounted and warrant investigation. Interestingly, miR-34c-3p has been shown to be a major miRNA induced during infection by H1N1 and H5N1 influenza viruses [50], although its role in influenza biology is presently incompletely understood. Thus, these findings have helped to identify important miRNA regulated kinase pathways required for influenza infection and replication.

## Discussion

The study of influenza virus biology has revealed complex mechanisms by which the influenza virus co-opts host cellular pathways to facilitate virus replication and evade the antiviral response. With the application of genome-wide screens, interactions between the virus and specific host cell components are now being identified and have led to new insights into viral and host interactions at different stages of the life cycle. A direct outcome of these studies can be the repurposing of old drugs for new conditions as was recently demonstrated for influenza [109].

A primary objective of this study was to identify human protein kinases that regulate influenza virus replication, and determine how miRNAs may govern their expression during influenza infection. A genome-wide siRNA screen of 720 HPK genes was evaluated. This screen identified 22 HPK that upon silencing led to increased or decreased influenza virus replication. Three HPKs (NPR, MAP3K1 and DYRK3) when silenced led to increased viral replication, suggesting that they have anti-viral activity. The remaining 19 HPKs when silenced decreased virus replication, suggesting that they are pro-viral and indispensable for viral replication. The preliminary hits identified from the primary screen were silenced using siRNAs targeting a novel seed site in the same gene and tested for impact on viral replication using multiple endpoint assays that evaluated viral genome, virus replication, and NP staining. A total of 18 HPKs (NPR2, MAP3K1, DYRK3, EPHA6, TPK1, PDK2, EXOSC10, NEK8, PLK4, SGK3, NEK3, PANK4, ITPKB, CDK13, CALM2, ADK, PKN3, and HK2) passed this validation and were tested with a seasonal strain of influenza virus where six HPKs that were identified as necessary for replication. It should be noted that other genes identified in the primary screen, i.e. CDK3, PRKAG3, ERBB4, and C9ORF96, were excluded from validation studies only because their expression was not silenced with the novel siRNA, and these HPKs may also have a role in virus replication. One primary screen hit, ADK, showed reduced M1 levels measured by qPCR, but did not validate in our NP localization analysis. Genomic changes would be expected to be subtle compared to changes in NP localization and could account for these findings. To date, five independent genome-wide screens in mammalian cells have characterized host factors important for influenza virus replication [13], [16]–[19]. A minimal overlap between screens was observed, but core pathways were found to be conserved. The minimal overlap between different virus strains is expected since the tempo of signal transduction and host gene expression is differentially induced by different virus strains which is linked to differences in replication dynamics and virus yield [110]. It is also likely that different influenza viruses may use alternate pathways for virus replication as host pathways were generally found to be conserved between screens [72]. This may be particularly relevant as A/WSN/33 which is a mouse-adapted virus and A/New Caledonia is a human virus.

The experimental approach used in this study to analyze influenza virus replication at 48 h post-siRNA transfection limits the findings to the later phases of the viral life cycle and may preclude HPKs that are important in the earliest phases of the viral life cycle. To avoid an extensive cross-talk due to cellular signaling post-infection, a very low MOI of infection was used to allow sufficient viral replication and avoid excessive cytopathic effect. Importantly, the screen in this study was validated using three endpoints to confirm the effect of host gene silencing on influenza virus replication. These endpoints included determining infectious virus titers as measured in MDCK cells, viral genome replication as determined by qPCR measurement of influenza M gene expression, and influenza NP as determined by high content analysis.

From the HPKs identified in the secondary validation screen, four pro-viral (NEK8, PLK4, SGK3 and CDK13) and two anti-viral (MAP3K1 and DYRK3) HPKs were assessed for miRNA regulation, and identified three HPKs (two pro-viral and one antiviral) (CDK13, MAP3K1 and PLK4) were found to have differential expression upon corresponding miRNA inhibitor/mimic treatment suggesting that these miRNAs likely regulate these HPKs. Inhibitor/mimic treatments for two genes DYRK3 and SGK3 did not cause significant changes in transcript expression and hence were not pursued further. miRNAs-1227 and -149* inhibitor/mimic modulated NEK8 transcript expression though differences between inhibitor/mimic were significant only for miR-149*. miR-149* treatment did not affect NEK8 protein expression however and hence was not studied further. miR-149* is known to induce apoptosis by repressing Akt1 and E2F1 [111] and hence inhibition of miR-149* may explain increased NEK8 transcript levels. Contrary to current dogma that miRNA inhibition relieves native miRNA inhibition and causes increased expression of target genes, and vice versa, it was observed that miRNA inhibition reduced transcript levels for CDK13, MAP3K1 and PLK4 while mimic treatment induced protein expression. This may be either by off-targeting by miRNA inhibitor/mimics studies or by stabilizing target gene expression by poorly understood alternate mechanisms [98], [112]–[114]. miR-548d inhibitor mediated MAPK1 transcript induction was not observed at the protein levels (Figure 5A–B). Expression of PLK4 transcript and protein were significantly modulated by miR-34c inhibitor/mimic and these also had a significant impact on viral replication (Figure 6C) suggesting that PLK4 is an important miR-34c target during influenza replication. Analysis of existing literature suggests that miR-34c alters PLK4 activity by modulating the activity of either p53 or by stabilizing PLK4 translation. Influenza infection has been shown to up regulate expression of a large number of proteins [115]–[120], and the genes expressing these proteins are likely regulated at some level by miRNAs.

While the findings in this study show that HPKs are important for influenza replication, and key HPKs are regulated by miRNAs that are also deregulated during influenza infection, the number of miRNAs that were validated to be affected was limited. This could be either due to differential temporal expression profiles of the target genes and the miRNAs investigated, and/or affected by cell type-specific features, or the regulation small enough as not to lead to a detectable phenotype. While preliminary pathway analysis identified several key pathways regulated by the HPKs identified in this study, detailed analysis is needed but outside the scope of this study. Given the large number of potential miRNA targets, it is unlikely that one miRNA governing one HPK gene could sufficiently explain a phenotype, i.e. increased or decreased influenza virus replication. The genetic screen in this study yielded 17 candidate HPK genes based on a 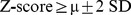; however, lowering the threshold to a 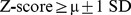 would have added an additional 37 HPK genes that overlapped with HPK genes discovered in related published screens [12]–[16]. Thus, it is likely that other HPK genes not identified in this study and the miRNAs governing their expression may be required or contribute to influenza virus replication.

There remains a gap in our understanding of the role of miRNA regulation of host genes, and how this interaction affects intracellular signaling pathways used during virus infection and replication. However, this study provides a framework for future studies, and contributes toward a better understanding of host-pathogen interactions which may help in accelerating the rational design of therapeutics aimed to control influenza infection and disease pathogenesis.

## Supporting Information

Figure S1 **RNA interference screen strategy for identification of host factors affecting influenza infection.** A) A549 cells were plated onto lyophilized siRNAs in 96-well flat-bottom plates and transiently transfected for 48 h with 50 nM siRNA. B) At 48 hours post-transfection, cells were infected with influenza virus A/WSN/33 (MOI 0.001). C) 48 hours post-infection, viral replication was assayed by titration of A549 cell supernatant on MDCK cells. Each siRNA given a score based on the number of wells with detectable virus, and primary hits determined using Z score analysis. D) Those hits were then validated using a novel siRNA to repeat the screen and E) phenotype was confirmed by influenza NP localization as well as assaying influenza viral genome replication via quantitative real time PCR detecting influenza M gene. F) Last, validated gene hits were associated with the cellular pathways they affect or intersect. MDCK, siNEG, non-target negative control siRNA; siMEK, mitogen-activated protein kinase kinase 2 siRNA positive control.
(TIF)

Figure S2 **(A) Optimization of siRNA knockdown using siMEK as a positive control. 48 hours after transfection with Dharmafect only (Mock) or Dharmafect+siMEK at 25 nM, 50 nM, and 100 nM concentrations in A549 cells, total RNA was extracted and used to quantify MEK-specific mRNA.** The transcript copies divided by GAPDH of gene silenced cells normalized to the same values of non-target control siRNA transfected samples. (B) z-score plot for HPK hits from primary screen. Calculated Z scores of the human kinase library identified primary hits (z scores ^3^2 and £ −2) whose silencing increased virus replication (positive Z score) and strongest hits that decreased virus replication (negative Z score). The position of each cellular kinase gene identified important for virus replication in the primary screen are indicated.
(TIF)

Figure S3 **Novel siRNAs targeting primary screen hits validate HPKs. siRNAs targeting a novel site in the HPK hits from the primary screen (Table S2) were transfected into A549 cells for 48** **hrs followed by RNA isolation and RT-qPCR using gene specific primers.** Fold changes were calculated relative to GAPDH as described in Materials and Methods previously. Values of control transfected cells are set as 0% silencing. The results are expressed as mean ± SD from a representative experiment performed in triplicate.
(TIF)

Figure S4 **Knockdown of miRNA by inhibitor treatment.** We used the miRNA let-7f (expressed at 750 copies in A549 cell (PMID 17699775) as a candidate for optimizing inhibitor/mimic transfection. A549 cells (2×10^4^ cells/well) were mock/transfected with 25 nM of let-7f inhibitor using Lipofectamine 2000 as per manufacturer’s protocol for 24 hrs. RNA was extracted using Trizol and then used for qRT-PCR using let-7f specific forward oligo (5′-TGAGGTAGTAGATTGTATAGTTAAAAA-3′) and Universal reverse oligo as per Ncode miRNA qRT-PCR kit. Five tenfold serial dilutions of let-7f specific cDNA in triplicate from uninfected A549 cells were run in parallel to calculate copy numbers. As per MIQE guidelines only standards with RSq >0.99, PCR efficiency 90–110% and slope between -3.1 to -3.4 were used to calculate copy numbers. Data is represented as fold change relative to mock from two independent experiments. Error bars represent SEM.
(TIF)

Figure S5 **HPK expression in response to Influenza virus infection.** A549 cells were infected with A/WSN/33 (MOI = 0.05) and RNA was isolated at 4 h, 12 h and 24 h post infection using Trizol as per manufacturer’s instructions. HPK expression was analyzed using gene specific qPCR primers and plotted relative to 18S rRNA expression. Data represent means from two experiments.
(TIF)

Figure S6 **Predicted pathways of miRNA regulation of HPK expression during influenza infection.** Schematic outline of cell cycle in mock infected cells. G1 to S phase transition is controlled by multiple factors of which Myc is a major regulator. Levels of PLK4 transcript and auto phosphorylated active PLK4 protein increase starting at S phase and peak at M phase. Active PLK4 targets its own degradation via proteasome mediated decay and is degraded.to complete cytokinesis and entry into G0/G1 phase. (B) Influenza (via NS1 protein) infection induces expression of p53 protein that induces miR-34c. miR-34c suppresses Myc mediated entry into S phase. Inhibition of miR-34c accelerates cell cycling and increased degradation of PLK4 protein and decreased influenza replication. miR-34c mimic arrests cells in G2/S phase by suppressing Myc leading to increased influenza replication.
(TIF)

Table S1 **Normalized scores, Z score analysis and cytotoxicity data for hits from host protein kinase screen.** Table listing the location, siRNA target gene symbol and raw and normalized scores for all the kinases tested in this manuscript. Raw scores were normalized across the library to calculate mean score, standard deviation and Z-scores. Cytotoxicity data as measured by Toxilight assay is also shown.
(DOCX)

Table S2 **Sequences of siRNAs used in the study.** Table shows accession numbers, gene symbol, gene id and siRNA sequences used for validation of hits from primary screen.
(DOCX)

Table S3 **Summary of hits overlapping with other influenza whole genome siRNA screens.** Data from other screens to identify host genes crucial for influenza virus replication were compared with the hits in our screen and are highlighted in red.
(DOCX)

Table S4 **Oligonucleotide sequences of all primers used in the manuscript.** Table lists the genes and the primers used for qPCR in this manuscript. Forward primers are appended with F and reverse primers are appended with R.
(DOCX)

Table S5 **List of miRNAs differentially expressed during influenza infection.** Table lists miRNAs shown to be differentially expressed during various influenza infections. Articles are referenced by PMIDs and miRNAs and targets if any are listed in adjoining columns.
(XLSX)

Table S6 **Details of seed matches between miRNAs and 3′-UTRs. miRNA and 3′UTR alignment details were mined from Targetscan (** www.targetscan.org **).** Details of seed match alignment, seed sequence etc are shown.
(XLSX)

Text S1 **Alignment of miR-548 family miRNA sequences.** Sequences of miR-548 family members were aligned using Clustal W application locally in BioEdit ver. 7.0 (Tom Hall). Alignment shows high degree of seed identity between multiple miR-548 members.
(RTF)

## References

[1] Thompson WW, Shay DK, Weintraub E, Brammer L, Bridges CB, et al (2004) Influenza-associated hospitalizations in the United States. JAMA 292: 1333–1340. 15367555 10.1001/jama.292.11.1333

[2] Thompson WW, Shay DK, Weintraub E, Brammer L, Cox N, et al (2003) Mortality associated with influenza and respiratory syncytial virus in the United States. JAMA 289: 179–186. 12517228 10.1001/jama.289.2.179

[3] MMWR (2010) Estimates of Deaths Associated with Seasonal Influenza - United States, 1976–2007. Atlanta, GA: Centers for Disease Control and Prevention. 1057–1062 p. 20798667

[4] Regoes RR, Bonhoeffer S (2006) Emergence of drug-resistant influenza virus: population dynamical considerations. Science 312: 389–391. 16627735 10.1126/science.1122947

[5] Beigel J, Bray M (2008) Current and future antiviral therapy of severe seasonal and avian influenza. Antiviral Res 78: 91–102. 18328578 10.1016/j.antiviral.2008.01.003 PMC2346583

[6] Conly J, Johnston B (2006) Ode to oseltamivir and amantadine? Can J Infect Dis Med Microbiol 17: 11–14. 18418477 10.1155/2006/106989 PMC2095051

[7] Hsieh HP, Hsu JT (2007) Strategies of development of antiviral agents directed against influenza virus replication. Curr Pharm Des 13: 3531–3542. 18220789 10.2174/138161207782794248

[8] Lackenby A, Thompson CI, Democratis J (2008) The potential impact of neuraminidase inhibitor resistant influenza. Curr Opin Infect Dis 21: 626–638. 18978531 10.1097/QCO.0b013e3283199797

[9] Osterhaus A, Fouchier R, Rimmelzwaan G (2011) Towards universal influenza vaccines? Philos Trans R Soc Lond B Biol Sci 366: 2766–2773. 21893539 10.1098/rstb.2011.0102 PMC3146782

[10] Schultz-Cherry S, Jones JC (2010) Influenza vaccines: the good, the bad, and the eggs. Adv Virus Res 77: 63–84. 20951870 10.1016/B978-0-12-385034-8.00003-X

[11] Palese P SM (2007) Fields Virology; Knipe DM HP, editor. Philadelphia: Raven.

[12] Brass AL, Huang IC, Benita Y, John SP, Krishnan MN, et al (2009) The IFITM proteins mediate cellular resistance to influenza A H1N1 virus, West Nile virus, and dengue virus. Cell 139: 1243–1254. 20064371 10.1016/j.cell.2009.12.017 PMC2824905

[13] Hao L, Sakurai A, Watanabe T, Sorensen E, Nidom CA, et al (2008) Drosophila RNAi screen identifies host genes important for influenza virus replication. Nature 454: 890–893. 18615016 10.1038/nature07151 PMC2574945

[14] Karlas A, Machuy N, Shin Y, Pleissner KP, Artarini A, et al.. (2010) Genome-wide RNAi screen identifies human host factors crucial for influenza virus replication. Nature. 10.1038/nature08760 20081832

[15] Konig R, Stertz S, Zhou Y, Inoue A, Hoffmann HH, et al (2009) Human host factors required for influenza virus replication. Nature 463: 813–817. 10.1038/nature08699 PMC2862546 20027183

[16] Shapira SD, Gat-Viks I, Shum BO, Dricot A, de Grace MM, et al (2009) A physical and regulatory map of host-influenza interactions reveals pathways in H1N1 infection. Cell 139: 1255–1267. 20064372 10.1016/j.cell.2009.12.018 PMC2892837

[17] Karlas A, Machuy N, Shin Y, Pleissner KP, Artarini A, et al. Genome-wide RNAi screen identifies human host factors crucial for influenza virus replication. Nature. 10.1038/nature08760 20081832

[18] Konig R, Stertz S, Zhou Y, Inoue A, Hoffmann HH, et al. Human host factors required for influenza virus replication. Nature 463: 813–817. 20027183 10.1038/nature08699 PMC2862546

[19] Brass AL, Dykxhoorn DM, Benita Y, Yan N, Engelman A, et al (2008) Identification of host proteins required for HIV infection through a functional genomic screen. Science 319: 921–926. 18187620 10.1126/science.1152725

[20] Arora DJ, Gasse N (1998) Influenza virus hemagglutinin stimulates the protein kinase C activity of human polymorphonuclear leucocytes. Arch Virol 143: 2029–2037. 9856090 10.1007/s007050050439

[21] Kunzelmann K, Beesley AH, King NJ, Karupiah G, Young JA, et al (2000) Influenza virus inhibits amiloride-sensitive Na+ channels in respiratory epithelia. Proc Natl Acad Sci U S A 97: 10282–10287. 10920189 10.1073/pnas.160041997 PMC27875

[22] Pleschka S, Wolff T, Ehrhardt C, Hobom G, Planz O, et al (2001) Influenza virus propagation is impaired by inhibition of the Raf/MEK/ERK signalling cascade. Nature cell biology 3: 301–305. 11231581 10.1038/35060098

[23] Pleschka S (2008) RNA viruses and the mitogenic Raf/MEK/ERK signal transduction cascade. Biol Chem 389: 1273–1282. 18713014 10.1515/BC.2008.145

[24] Marjuki H, Alam MI, Ehrhardt C, Wagner R, Planz O, et al (2006) Membrane accumulation of influenza A virus hemagglutinin triggers nuclear export of the viral genome via protein kinase Calpha-mediated activation of ERK signaling. J Biol Chem 281: 16707–16715. 16608852 10.1074/jbc.M510233200

[25] Ludwig S, Wolff T, Ehrhardt C, Wurzer WJ, Reinhardt J, et al (2004) MEK inhibition impairs influenza B virus propagation without emergence of resistant variants. Febs Letters 561: 37–43. 15013748 10.1016/S0014-5793(04)00108-5

[26] Wei L, Sandbulte MR, Thomas PG, Webby RJ, Homayouni R, et al (2006) NFkappaB negatively regulates interferon-induced gene expression and anti-influenza activity. J Biol Chem 281: 11678–11684. 16517601 10.1074/jbc.M513286200 PMC1457055

[27] Wang X, Li M, Zheng H, Muster T, Palese P, et al (2000) Influenza A virus NS1 protein prevents activation of NF-kappaB and induction of alpha/beta interferon. J Virol 74: 11566–11573. 11090154 10.1128/jvi.74.24.11566-11573.2000 PMC112437

[28] Pinto R, Herold S, Cakarova L, Hoegner K, Lohmeyer J, et al (2011) Inhibition of influenza virus-induced NF-kappaB and Raf/MEK/ERK activation can reduce both virus titers and cytokine expression simultaneously in vitro and in vivo. Antiviral Res 92: 45–56. 21641936 10.1016/j.antiviral.2011.05.009

[29] Pauli EK, Schmolke M, Wolff T, Viemann D, Roth J, et al (2008) Influenza A virus inhibits type I IFN signaling via NF-kappaB-dependent induction of SOCS-3 expression. PLoS Pathog 4: e1000196. 18989459 10.1371/journal.ppat.1000196 PMC2572141

[30] Pahl HL, Baeuerle PA (1995) Expression of influenza virus hemagglutinin activates transcription factor NF-kappa B. J Virol. 69: 1480–1484. 10.1128/jvi.69.3.1480-1484.1995 PMC188737 7853480

[31] Nimmerjahn F, Dudziak D, Dirmeier U, Hobom G, Riedel A, et al (2004) Active NF-kappaB signalling is a prerequisite for influenza virus infection. J Gen Virol 85: 2347–2356. 15269376 10.1099/vir.0.79958-0

[32] Ludwig S, Planz O (2008) Influenza viruses and the NF-kappaB signaling pathway - towards a novel concept of antiviral therapy. Biol Chem 389: 1307–1312. 18713017 10.1515/BC.2008.148

[33] Kumar N, Xin ZT, Liang Y, Ly H (2008) NF-kappaB signaling differentially regulates influenza virus RNA synthesis. J Virol 82: 9880–9889. 18701591 10.1128/JVI.00909-08 PMC2566266

[34] Flory E, Kunz M, Scheller C, Jassoy C, Stauber R, et al (2000) Influenza virus-induced NF-kappaB-dependent gene expression is mediated by overexpression of viral proteins and involves oxidative radicals and activation of IkappaB kinase. J Biol Chem 275: 8307–8314. 10722660 10.1074/jbc.275.12.8307

[35] Majde JA (2000) Viral double-stranded RNA, cytokines, and the flu. J Interferon Cytokine Res 20: 259–272. 10762073 10.1089/107999000312397

[36] Chu WM, Ostertag D, Li ZW, Chang L, Chen Y, et al (1999) JNK2 and IKKbeta are required for activating the innate response to viral infection. Immunity 11: 721–731. 10626894 10.1016/s1074-7613(00)80146-6

[37] Ludwig S, Pleschka S, Planz O, Wolff T (2006) Ringing the alarm bells: signalling and apoptosis in influenza virus infected cells. Cell Microbiol 8: 375–386. 16469051 10.1111/j.1462-5822.2005.00678.x

[38] Filipowicz W, Bhattacharyya SN, Sonenberg N (2008) Mechanisms of post-transcriptional regulation by microRNAs: are the answers in sight? Nat Rev Genet 9: 102–114. 18197166 10.1038/nrg2290

[39] Friedman RC, Farh KK, Burge CB, Bartel DP (2009) Most mammalian mRNAs are conserved targets of microRNAs. Genome Res 19: 92–105. 18955434 10.1101/gr.082701.108 PMC2612969

[40] Ambros V (2004) The functions of animal microRNAs. Nature 431: 350–355. 15372042 10.1038/nature02871

[41] Cui Q, Yu Z, Purisima EO, Wang E (2006) Principles of microRNA regulation of a human cellular signaling network. Mol Syst Biol 2: 46–46. 16969338 10.1038/msb4100089 PMC1681519

[42] Hsu C-W, Juan H-F, Huang H-C (2008) Characterization of microRNA-regulated protein-protein interaction network. Proteomics 8: 1975–1979. 18491312 10.1002/pmic.200701004

[43] Inui M, Martello G, Piccolo S (2010) MicroRNA control of signal transduction. Nat Rev Mol Cell Biol 11: 252–263. 20216554 10.1038/nrm2868

[44] Lagos D, Pollara G, Henderson S, Gratrix F, Fabani M, et al. miR-132 regulates antiviral innate immunity through suppression of the p300 transcriptional co-activator. Nature cell biology 12: 513–519. 10.1038/ncb2054 20418869

[45] Zheng SQ, Li YX, Zhang Y, Li X, Tang H MiR-101 regulates HSV-1 replication by targeting ATP5B. Antiviral Res 89: 219–226. 21291913 10.1016/j.antiviral.2011.01.008

[46] Yeung ML, Bennasser Y, Myers TG, Jiang G, Benkirane M, et al (2005) Changes in microRNA expression profiles in HIV-1-transfected human cells. Retrovirology 2: 81. 16381609 10.1186/1742-4690-2-81 PMC1352379

[47] Triboulet R, Mari B, Lin YL, Chable-Bessia C, Bennasser Y, et al (2007) Suppression of microRNA-silencing pathway by HIV-1 during virus replication. Science 315: 1579–1582. 17322031 10.1126/science.1136319

[48] Liu H, Song L, Huang W (2010) MiR26a and miR939 regulate the replication of H1N1 influenza virus in MDCK cell. Wei Sheng Wu Xue Bao 50: 1399–1405. 21141477

[49] Scaria V, Hariharan M, Maiti S, Pillai B, Brahmachari SK (2006) Host-virus interaction: a new role for microRNAs. Retrovirology 3: 68. 17032463 10.1186/1742-4690-3-68 PMC1626483

[50] Loveday EK, Svinti V, Diederich S, Pasick J, Jean F (2012) Temporal- and strain-specific host microRNA molecular signatures associated with swine-origin H1N1 and avian-origin H7N7 influenza A virus infection. J Virol 86: 6109–6122. 22438559 10.1128/JVI.06892-11 PMC3372180

[51] Fang J, Hao Q, Liu L, Li Y, Wu J, et al (2012) Epigenetic changes mediated by microRNA miR29 activate cyclooxygenase 2 and lambda-1 interferon production during viral infection. J Virol 86: 1010–1020. 22072783 10.1128/JVI.06169-11 PMC3255816

[52] Munding JB, Adai AT, Maghnouj A, Urbanik A, Zollner H, et al (2012) Global microRNA expression profiling of microdissected tissues identifies miR-135b as a novel biomarker for pancreatic ductal adenocarcinoma. Int J Cancer 131: E86–95. 21953293 10.1002/ijc.26466

[53] Buggele WA, Johnson KE, Horvath CM (2012) Influenza A virus infection of human respiratory cells induces primary microRNA expression. J Biol Chem 287: 31027–31040. 22822053 10.1074/jbc.M112.387670 PMC3438935

[54] Guan Z, Shi N, Song Y, Zhang X, Zhang M, et al (2012) Induction of the cellular microRNA-29c by influenza virus contributes to virus-mediated apoptosis through repression of antiapoptotic factors BCL2L2. Biochem Biophys Res Commun 425: 662–667. 22850539 10.1016/j.bbrc.2012.07.114

[55] Woolcock PR (2008) Avian influenza virus isolation and propagation in chicken eggs. Methods Mol Biol 436: 35–46. 18370039 10.1007/978-1-59745-279-3_6

[56] Matrosovich M, Matrosovich T, Garten W, Klenk HD (2006) New low-viscosity overlay medium for viral plaque assays. Virology Journal 3: 63. 16945126 10.1186/1743-422X-3-63 PMC1564390

[57] Reed LJ MH (1938) A simple method of estimating fifty percent endpoints. The American Journal of Hygiene 27: 493–497.

[58] Haney SA (2007) Increasing the robustness and validity of RNAi screens. Pharmacogenomics 8: 1037–1049. 17716236 10.2217/14622416.8.8.1037

[59] Cottey R, Rowe CA, Bender BS (2001) Influenza virus. Curr Protoc Immunol Chapter 19: Unit 19 11. 10.1002/0471142735.im1911s42 18432752

[60] Hirst GK (1942) Adsorption of Influenza Hemagglutinins and Virus by Red Blood Cells. J Exp Med 76: 195–209. 19871229 10.1084/jem.76.2.195 PMC2135226

[61] Spackman E, Senne DA, Myers TJ, Bulaga LL, Garber LP, et al (2002) Development of a real-time reverse transcriptase PCR assay for type A influenza virus and the avian H5 and H7 hemagglutinin subtypes. J Clin Microbiol 40: 3256–3260. 12202562 10.1128/JCM.40.9.3256-3260.2002 PMC130722

[62] Bustin SA, Benes V, Garson JA, Hellemans J, Huggett J, et al (2009) The MIQE guidelines: minimum information for publication of quantitative real-time PCR experiments. Clin Chem 55: 611–622. 19246619 10.1373/clinchem.2008.112797

[63] Dweep H, Sticht C, Pandey P, Gretz N (2011) miRWalk–database: prediction of possible miRNA binding sites by "walking" the genes of three genomes. J Biomed Inform 44: 839–847. 21605702 10.1016/j.jbi.2011.05.002

[64] Lewis BP, Burge CB, Bartel DP (2005) Conserved seed pairing, often flanked by adenosines, indicates that thousands of human genes are microRNA targets. Cell 120: 15–20. 15652477 10.1016/j.cell.2004.12.035

[65] Johnson CD, Esquela-Kerscher A, Stefani G, Byrom M, Kelnar K, et al (2007) The let-7 microRNA represses cell proliferation pathways in human cells. Cancer Res 67: 7713–7722. 17699775 10.1158/0008-5472.CAN-07-1083

[66] Esau CC (2008) Inhibition of microRNA with antisense oligonucleotides. Methods 44: 55–60. 18158133 10.1016/j.ymeth.2007.11.001

[67] Krutzfeldt J, Rajewsky N, Braich R, Rajeev KG, Tuschl T, et al (2005) Silencing of microRNAs in vivo with ‘antagomirs’. Nature 438: 685–689. 16258535 10.1038/nature04303

[68] Vermeulen A, Robertson B, Dalby AB, Marshall WS, Karpilow J, et al (2007) Double-stranded regions are essential design components of potent inhibitors of RISC function. RNA 13: 723–730. 17400817 10.1261/rna.448107 PMC1852807

[69] Xiao J, Yang B, Lin H, Lu Y, Luo X, et al (2007) Novel approaches for gene-specific interference via manipulating actions of microRNAs: examination on the pacemaker channel genes HCN2 and HCN4. J Cell Physiol 212: 285–292. 17516552 10.1002/jcp.21062

[70] Droebner K, Pleschka S, Ludwig S, Planz O (2011) Antiviral activity of the MEK-inhibitor U0126 against pandemic H1N1v and highly pathogenic avian influenza virus in vitro and in vivo. Antiviral Res 92: 195–203. 21854809 10.1016/j.antiviral.2011.08.002

[71] Sigoillot FD, King RW (2011) Vigilance and validation: Keys to success in RNAi screening. ACS chemical biology 6: 47–60. 21142076 10.1021/cb100358f PMC3306249

[72] Min JY, Subbarao K (2010) Cellular targets for influenza drugs. Nat Biotechnol 28: 239–240. 20212486 10.1038/nbt0310-239 PMC3397661

[73] Straka M, Boese Q (2010) Current Topics in RNAi: Why Rational Pooling of siRNAs is SMART. Thermo Fisher Scientific Inc.

[74] Jiang W, Wang Q, Chen S, Gao S, Song L, et al (2013) Influenza A virus NS1 induces G0/G1 cell cycle arrest by inhibiting the expression and activity of RhoA protein. J Virol 87: 3039–3052. 23283961 10.1128/JVI.03176-12 PMC3592114

[75] Parnell G, McLean A, Booth D, Huang S, Nalos M, et al (2011) Aberrant cell cycle and apoptotic changes characterise severe influenza A infection–a meta-analysis of genomic signatures in circulating leukocytes. PLoS One 6: e17186. 21408152 10.1371/journal.pone.0017186 PMC3050844

[76] He Y, Xu K, Keiner B, Zhou J, Czudai V, et al (2010) Influenza A virus replication induces cell cycle arrest in G0/G1 phase. J Virol 84: 12832–12840. 20861262 10.1128/JVI.01216-10 PMC3004346

[77] Chen HH, Wong YH, Geneviere AM, Fann MJ (2007) CDK13/CDC2L5 interacts with L-type cyclins and regulates alternative splicing. Biochem Biophys Res Commun 354: 735–740. 17261272 10.1016/j.bbrc.2007.01.049

[78] Berro R, Pedati C, Kehn-Hall K, Wu W, Klase Z, et al (2008) CDK13, a new potential human immunodeficiency virus type 1 inhibitory factor regulating viral mRNA splicing. J Virol 82: 7155–7166. 18480452 10.1128/JVI.02543-07 PMC2446983

[79] Holland PM, Milne A, Garka K, Johnson RS, Willis C, et al (2002) Purification, cloning, and characterization of Nek8, a novel NIMA-related kinase, and its candidate substrate Bicd2. J Biol Chem 277: 16229–16240. 11864968 10.1074/jbc.M108662200

[80] Zalli D, Bayliss R, Fry AM (2012) The Nek8 protein kinase, mutated in the human cystic kidney disease nephronophthisis, is both activated and degraded during ciliogenesis. Hum Mol Genet 21: 1155–1171. 22106379 10.1093/hmg/ddr544 PMC3277313

[81] Holland AJ, Fachinetti D, Da Cruz S, Zhu Q, Vitre B, et al (2012) Polo-like kinase 4 controls centriole duplication but does not directly regulate cytokinesis. Mol Biol Cell 23: 1838–1845. 22456511 10.1091/mbc.E11-12-1043 PMC3350549

[82] Sillibourne JE, Bornens M (2010) Polo-like kinase 4: the odd one out of the family. Cell Div 5: 25. 20920249 10.1186/1747-1028-5-25 PMC2955731

[83] Sillibourne JE, Tack F, Vloemans N, Boeckx A, Thambirajah S, et al (2010) Autophosphorylation of polo-like kinase 4 and its role in centriole duplication. Mol Biol Cell 21: 547–561. 20032307 10.1091/mbc.E09-06-0505 PMC2820420

[84] Holland AJ, Fachinetti D, Zhu Q, Bauer M, Verma IM, et al (2012) The autoregulated instability of Polo-like kinase 4 limits centrosome duplication to once per cell cycle. Genes Dev 26: 2684–2689. 23249732 10.1101/gad.207027.112 PMC3533073

[85] Yamauchi Y, Boukari H, Banerjee I, Sbalzarini IF, Horvath P, et al (2011) Histone Deacetylase 8 Is Required for Centrosome Cohesion and Influenza A Virus Entry. PLoS Pathog 7: e1002316. 22046129 10.1371/journal.ppat.1002316 PMC3203190

[86] Alamares-Sapuay JG, Martinez-Gil L, Stertz S, Miller MS, Shaw ML, et al (2013) Serum- and glucocorticoid-regulated kinase 1 is required for nuclear export of the ribonucleoprotein of influenza a virus. J Virol 87: 6020–6026. 23487453 10.1128/JVI.01258-12 PMC3648197

[87] Liu M, Chen L, Chan TH, Wang J, Li Y, et al (2012) Serum and glucocorticoid kinase 3 at 8q13.1 promotes cell proliferation and survival in hepatocellular carcinoma. Hepatology 55: 1754–1765. 22262416 10.1002/hep.25584

[88] Yoshida R, Takaesu G, Yoshida H, Okamoto F, Yoshioka T, et al (2008) TRAF6 and MEKK1 play a pivotal role in the RIG-I-like helicase antiviral pathway. J Biol Chem 283: 36211–36220. 18984593 10.1074/jbc.M806576200 PMC2662295

[89] Labuda T, Christensen JP, Rasmussen S, Bonnesen B, Karin M, et al (2006) MEK kinase 1 is a negative regulator of virus-specific CD8(+) T cells. Eur J Immunol 36: 2076–2084. 16761309 10.1002/eji.200535163

[90] Guo X, Williams JG, Schug TT, Li X (2010) DYRK1A and DYRK3 promote cell survival through phosphorylation and activation of SIRT1. J Biol Chem 285: 13223–13232. 20167603 10.1074/jbc.M110.102574 PMC2857074

[91] Mata MA, Satterly N, Versteeg GA, Frantz D, Wei S, et al (2011) Chemical inhibition of RNA viruses reveals REDD1 as a host defense factor. Nat Chem Biol 7: 712–719. 21909097 10.1038/nchembio.645 PMC3329801

[92] Murray JL, McDonald NJ, Sheng J, Shaw MW, Hodge TW, et al (2012) Inhibition of influenza A virus replication by antagonism of a PI3K-AKT-mTOR pathway member identified by gene-trap insertional mutagenesis. Antivir Chem Chemother 22: 205–215. 22374988 10.3851/IMP2080

[93] Wippich F, Bodenmiller B, Trajkovska MG, Wanka S, Aebersold R, et al (2013) Dual specificity kinase DYRK3 couples stress granule condensation/dissolution to mTORC1 signaling. Cell 152: 791–805. 23415227 10.1016/j.cell.2013.01.033

[94] Xu Y, Li F, Zhang B, Zhang K, Zhang F, et al (2010) MicroRNAs and target site screening reveals a pre-microRNA-30e variant associated with schizophrenia. Schizophr Res 119: 219–227. 20347265 10.1016/j.schres.2010.02.1070

[95] Li Y, Li J, Belisle S, Baskin CR, Tumpey TM, et al.. (2011) Differential microRNA expression and virulence of avian, 1918 reassortant, and reconstructed 1918 influenza A viruses. Virology. 10.1016/j.virol.2011.09.011 PMC3210927 21999992

[96] Lee SM, Gardy JL, Cheung CY, Cheung TK, Hui KP, et al (2009) Systems-level comparison of host-responses elicited by avian H5N1 and seasonal H1N1 influenza viruses in primary human macrophages. PLoS One 4: e8072. 20011590 10.1371/journal.pone.0008072 PMC2788213

[97] Chang WL, Coro ES, Rau FC, Xiao Y, Erle DJ, et al (2007) Influenza virus infection causes global respiratory tract B cell response modulation via innate immune signals. J Immunol 178: 1457–1467. 17237394 10.4049/jimmunol.178.3.1457

[98] Lu H, Buchan RJ, Cook SA (2010) MicroRNA-223 regulates Glut4 expression and cardiomyocyte glucose metabolism. Cardiovasc Res 86: 410–420. 20080987 10.1093/cvr/cvq010

[99] Bakre A, Mitchell P, Coleman JK, Jones LP, Saavedra G, et al (2012) Respiratory syncytial virus modifies microRNAs regulating host genes that affect virus replication. J Gen Virol 93: 2346–2356. 22894925 10.1099/vir.0.044255-0 PMC3542124

[100] Meliopoulos VA, Andersen LE, Brooks P, Yan X, Bakre A, et al (2012) MicroRNA regulation of human protease genes essential for influenza virus replication. PLoS One 7: e37169. 22606348 10.1371/journal.pone.0037169 PMC3351457

[101] Liang T, Guo L, Liu C (2012) Genome-wide analysis of mir-548 gene family reveals evolutionary and functional implications. J Biomed Biotechnol 2012: 679563. 23091353 10.1155/2012/679563 PMC3468316

[102] Li Y, Xie J, Xu X, Wang J, Ao F, et al (2013) MicroRNA-548 down-regulates host antiviral response via direct targeting of IFN-λ1. Protein & Cell 4: 130–141. 23150165 10.1007/s13238-012-2081-y PMC4875363

[103] Munoz-Fontela C, Pazos M, Delgado I, Murk W, Mungamuri SK, et al (2011) p53 serves as a host antiviral factor that enhances innate and adaptive immune responses to influenza A virus. J Immunol 187: 6428–6436. 22105999 10.4049/jimmunol.1101459 PMC3275346

[104] Turpin E, Luke K, Jones J, Tumpey T, Konan K, et al (2005) Influenza virus infection increases p53 activity: role of p53 in cell death and viral replication. J Virol 79: 8802–8811. 15994774 10.1128/JVI.79.14.8802-8811.2005 PMC1168730

[105] Wang X, Shen Y, Qiu Y, Shi Z, Shao D, et al (2010) The non-structural (NS1) protein of influenza A virus associates with p53 and inhibits p53-mediated transcriptional activity and apoptosis. Biochem Biophys Res Commun 395: 141–145. 20361939 10.1016/j.bbrc.2010.03.160

[106] Cannell IG, Kong YW, Johnston SJ, Chen ML, Collins HM, et al (2010) p38 MAPK/MK2-mediated induction of miR-34c following DNA damage prevents Myc-dependent DNA replication. Proc Natl Acad Sci U S A 107: 5375–5380. 20212154 10.1073/pnas.0910015107 PMC2851793

[107] Ghosh T, Soni K, Scaria V, Halimani M, Bhattacharjee C, et al (2008) MicroRNA-mediated up-regulation of an alternatively polyadenylated variant of the mouse cytoplasmic {beta}-actin gene. Nucleic Acids Res 36: 6318–6332. 18835850 10.1093/nar/gkn624 PMC2577349

[108] Cannell IG, Bushell M (2010) Regulation of Myc by miR-34c: A mechanism to prevent genomic instability? Cell Cycle 9: 2726–2730. 20603603

[109] Perwitasari O, Yan X, Johnson S, White C, Brooks P, et al (2013) Targeting organic anion transporter 3 with probenecid as a novel anti-influenza a virus strategy. Antimicrob Agents Chemother 57: 475–483. 23129053 10.1128/AAC.01532-12 PMC3535968

[110] Heynisch B, Frensing T, Heinze K, Seitz C, Genzel Y, et al (2010) Differential activation of host cell signalling pathways through infection with two variants of influenza A/Puerto Rico/8/34 (H1N1) in MDCK cells. Vaccine 28: 8210–8218. 20691654 10.1016/j.vaccine.2010.07.076

[111] Lin RJ, Lin YC, Yu AL (2010) miR-149* induces apoptosis by inhibiting Akt1 and E2F1 in human cancer cells. Mol Carcinog 49: 719–727. 20623644 10.1002/mc.20647

[112] Kang HW, Wang F, Wei Q, Zhao YF, Liu M, et al (2012) miR-20a promotes migration and invasion by regulating TNKS2 in human cervical cancer cells. FEBS Lett 586: 897–904. 22449978 10.1016/j.febslet.2012.02.020

[113] Truesdell SS, Mortensen RD, Seo M, Schroeder JC, Lee JH, et al (2012) MicroRNA-mediated mRNA Translation Activation in Quiescent Cells and Oocytes Involves Recruitment of a Nuclear microRNP. Sci Rep 2: 842. 23150790 10.1038/srep00842 PMC3496365

[114] Vasudevan S (2012) Posttranscriptional upregulation by microRNAs. Wiley Interdiscip Rev RNA 3: 311–330. 22072587 10.1002/wrna.121

[115] Liu L, Zhou J, Wang Y, Mason RJ, Funk CJ, et al (2012) Proteome alterations in primary human alveolar macrophages in response to influenza A virus infection. J Proteome Res 11: 4091–4101. 22709384 10.1021/pr3001332 PMC3412919

[116] Kroeker AL, Ezzati P, Halayko AJ, Coombs KM (2012) Response of primary human airway epithelial cells to influenza infection: a quantitative proteomic study. J Proteome Res 11: 4132–4146. 22694362 10.1021/pr300239r PMC3411195

[117] Dove BK, Surtees R, Bean TJ, Munday D, Wise HM, et al (2012) A quantitative proteomic analysis of lung epithelial (A549) cells infected with 2009 pandemic influenza A virus using stable isotope labelling with amino acids in cell culture. Proteomics 12: 1431–1436. 22585751 10.1002/pmic.201100470

[118] Zhu J, Zou W, Jia G, Zhou H, Hu Y, et al (2012) Analysis of cellular proteome alterations in porcine alveolar macrophage cells infected with 2009 (H1N1) and classical swine H1N1 influenza viruses. J Proteomics 75: 1732–1741. 22202185 10.1016/j.jprot.2011.12.012

[119] Lietzen N, Ohman T, Rintahaka J, Julkunen I, Aittokallio T, et al (2011) Quantitative subcellular proteome and secretome profiling of influenza A virus-infected human primary macrophages. PLoS Pathog 7: e1001340. 21589892 10.1371/journal.ppat.1001340 PMC3093355

[120] Zhang L, Zhang X, Ma Q, Ma F, Zhou H (2010) Transcriptomics and proteomics in the study of H1N1 2009. Genomics Proteomics Bioinformatics 8: 139–144. 20970742 10.1016/S1672-0229(10)60016-2 PMC5054133

