# Supplementary figures and images for: Identification of Host Kinase Genes Required for Influenza Virus Replication and the Regulatory Role of MicroRNAs
Source: PLoS One. 2013 Jun 21;8(6):e66796. doi: 10.1371/journal.pone.0066796 (PMC3689682; doi:10.1371/journal.pone.0066796)

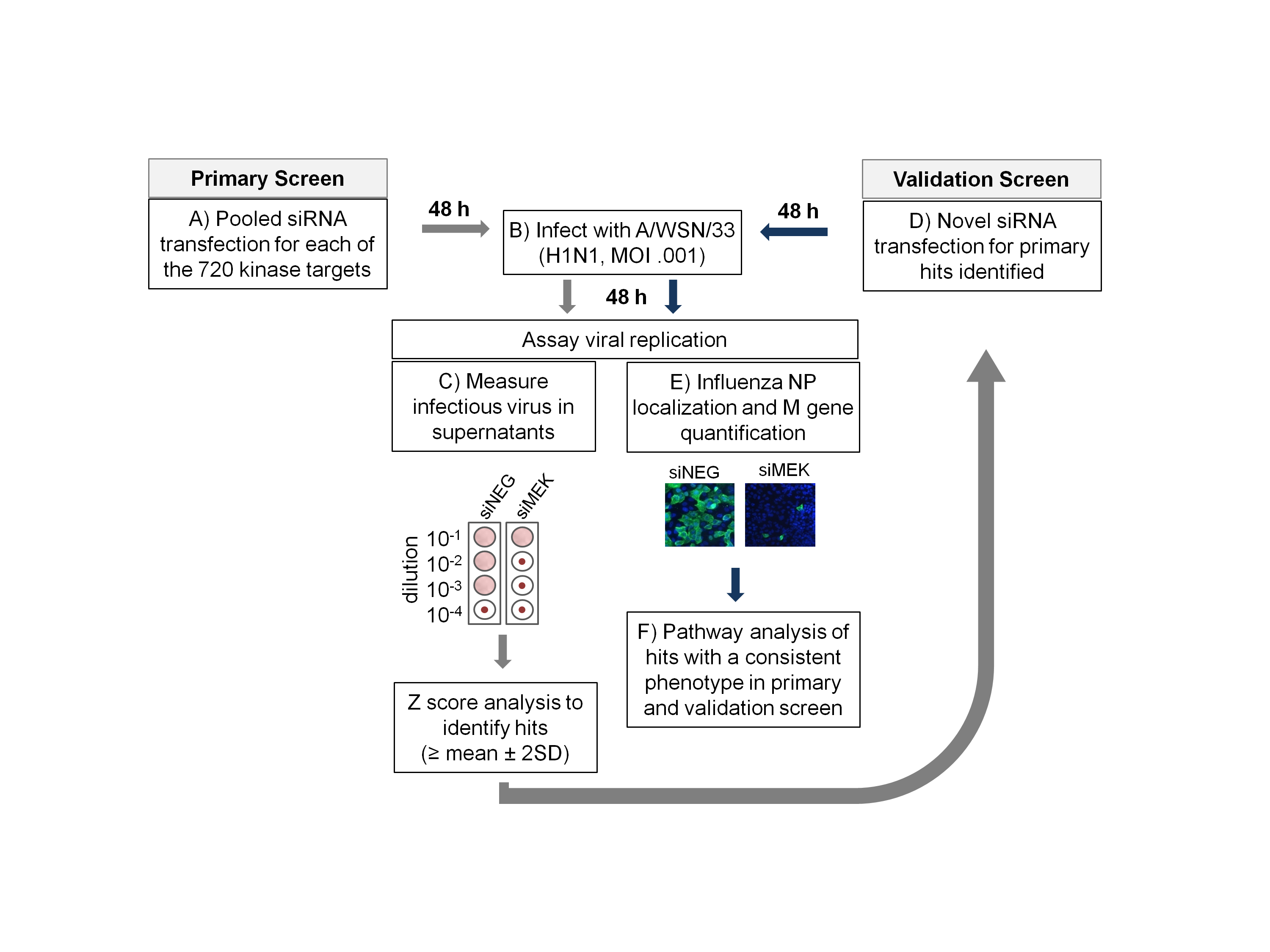

Supplement: Figure S1 — RNA interference screen strategy for identification of host factors affecting influenza infection. A) A549 cells were plated onto lyophilized siRNAs in 96-well flat-bottom plates and transiently transfected for 48 h with 50 nM siRNA. B) At 48 hours post-transfection, cells were infected with influenza virus A/WSN/33 (MOI 0.001). C) 48 hours post-infection, viral replication was assayed by titration of A549 cell supernatant on MDCK cells. Each siRNA given a score based on the number of wells with detectable virus, and primary hits determined using Z score analysis. D) Those hits were then validated using a novel siRNA to repeat the screen and E) phenotype was confirmed by influenza NP localization as well as assaying influenza viral genome replication via quantitative real time PCR detecting influenza M gene. F) Last, validated gene hits were associated with the cellular pathways they affect or intersect. MDCK, siNEG, non-target negative control siRNA; siMEK, mitogen-activated protein kinase kinase 2 siRNA positive control. (TIF) [file pone.0066796.s001.tif]

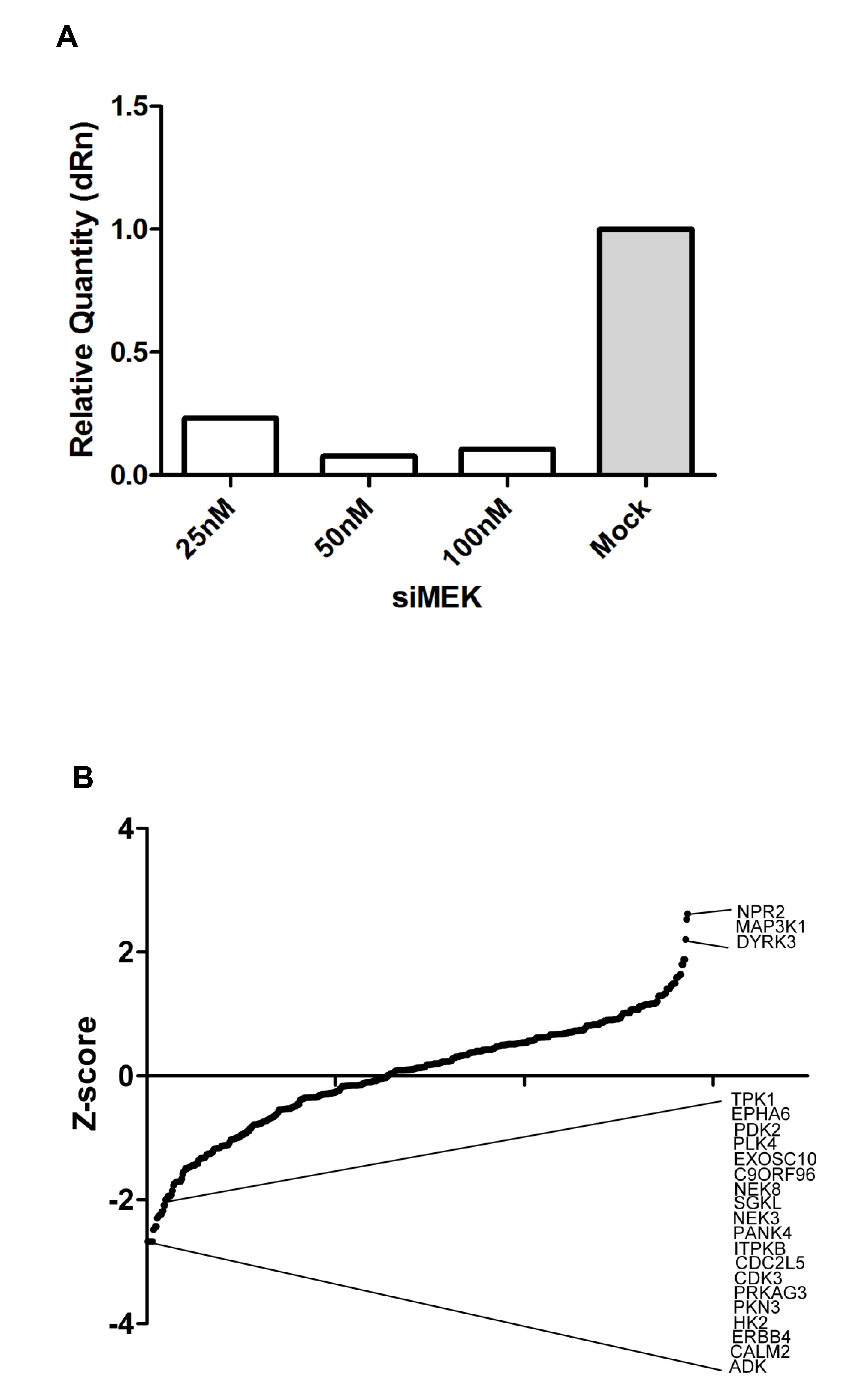

Supplement: Figure S2 — (A) Optimization of siRNA knockdown using siMEK as a positive control. 48 hours after transfection with Dharmafect only (Mock) or Dharmafect+siMEK at 25 nM, 50 nM, and 100 nM concentrations in A549 cells, total RNA was extracted and used to quantify MEK-specific mRNA. The transcript copies divided by GAPDH of gene silenced cells normalized to the same values of non-target control siRNA transfected samples. (B) z-score plot for HPK hits from primary screen. Calculated Z scores of the human kinase library identified primary hits (z scores 32 and £ −2) whose silencing increased virus replication (positive Z score) and strongest hits that decreased virus replication (negative Z score). The position of each cellular kinase gene identified important for virus replication in the primary screen are indicated. (TIF) [file pone.0066796.s002.tif]

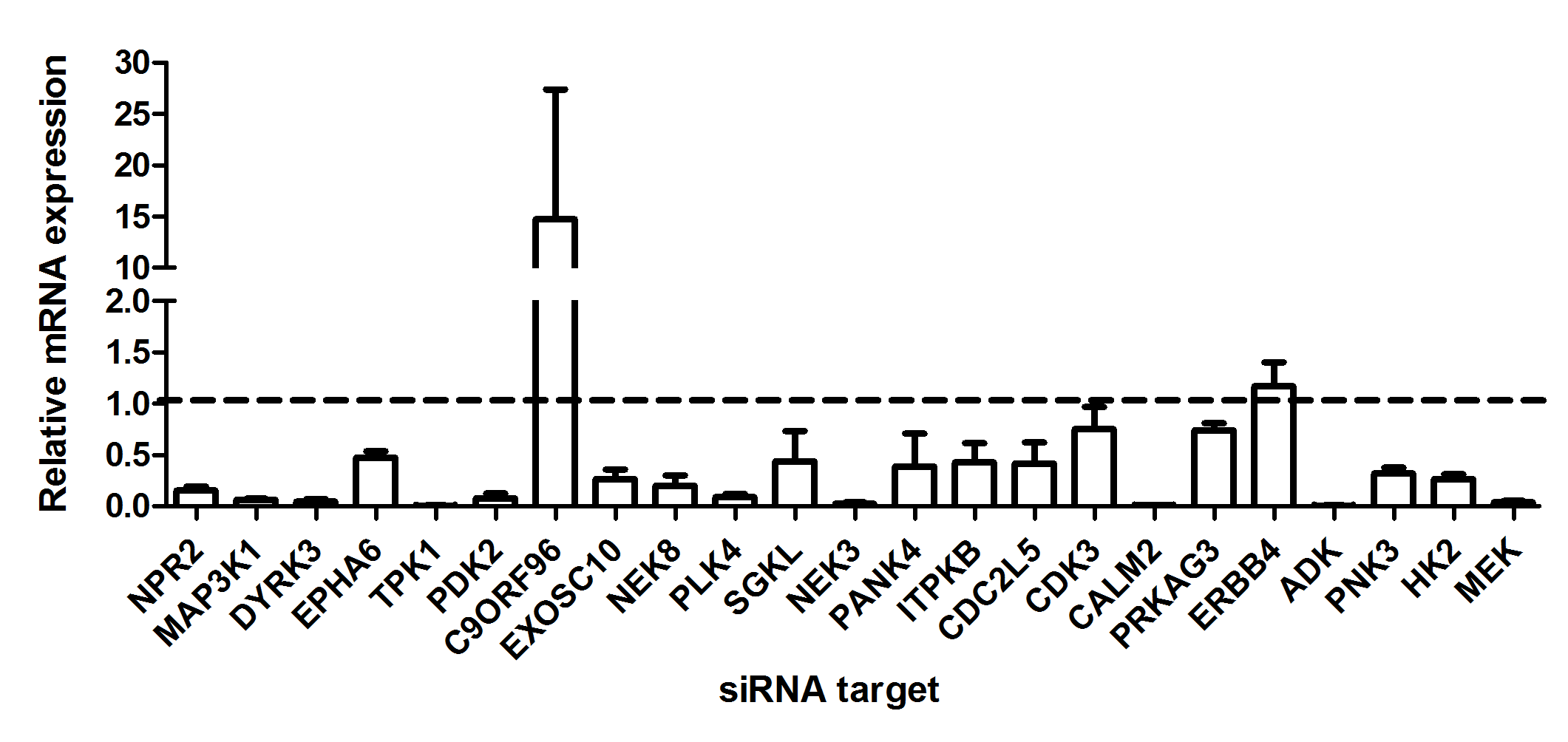

Supplement: Figure S3 — Novel siRNAs targeting primary screen hits validate HPKs. siRNAs targeting a novel site in the HPK hits from the primary screen (Table S2) were transfected into A549 cells for 48 hrs followed by RNA isolation and RT-qPCR using gene specific primers. Fold changes were calculated relative to GAPDH as described in Materials and Methods previously. Values of control transfected cells are set as 0% silencing. The results are expressed as mean ± SD from a representative experiment performed in triplicate. (TIF) [file pone.0066796.s003.tif]

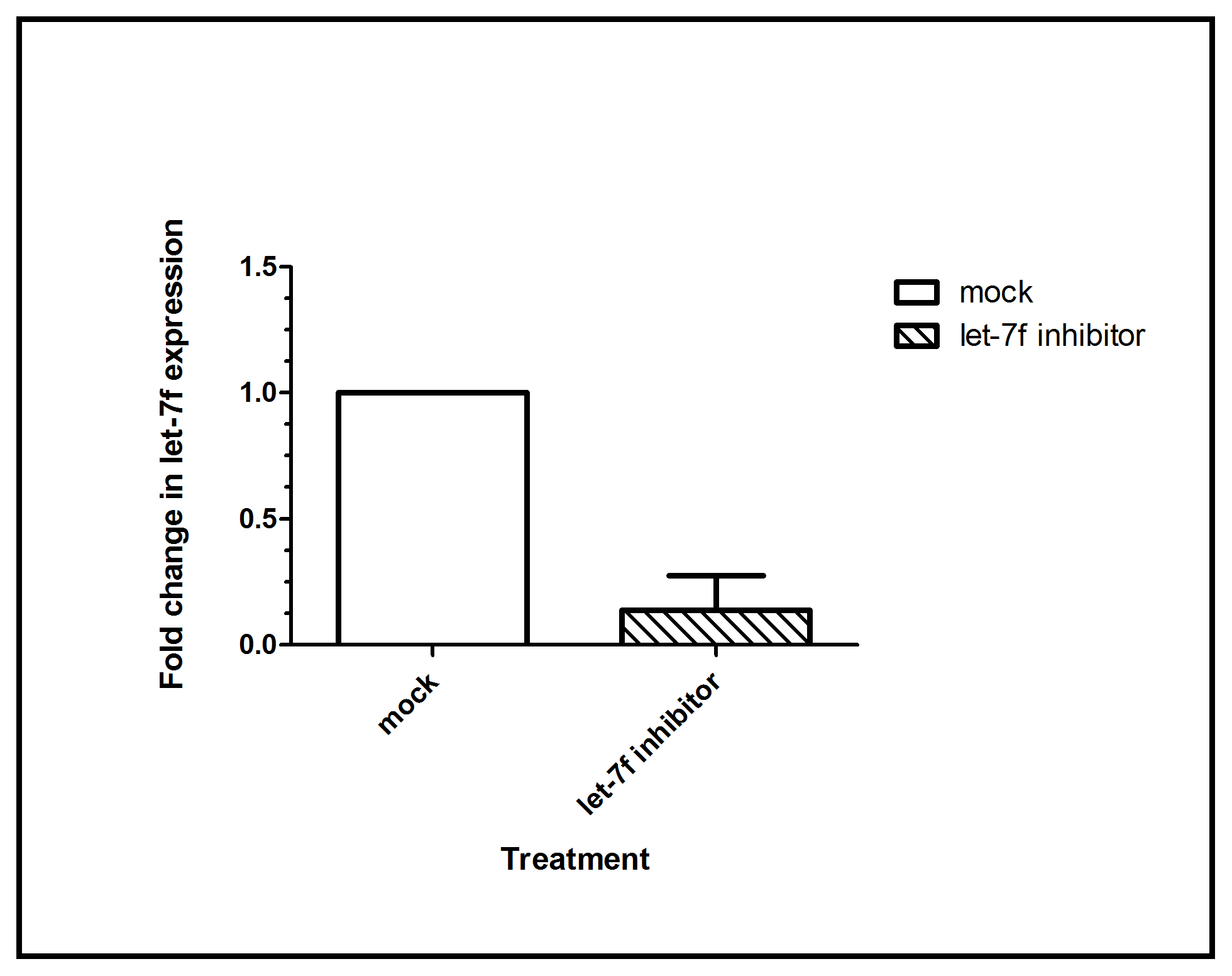

Supplement: Figure S4 — Knockdown of miRNA by inhibitor treatment. We used the miRNA let-7f (expressed at 750 copies in A549 cell (PMID 17699775) as a candidate for optimizing inhibitor/mimic transfection. A549 cells (2×10^4^ cells/well) were mock/transfected with 25 nM of let-7f inhibitor using Lipofectamine 2000 as per manufacturer’s protocol for 24 hrs. RNA was extracted using Trizol and then used for qRT-PCR using let-7f specific forward oligo (5′-TGAGGTAGTAGATTGTATAGTTAAAAA-3′) and Universal reverse oligo as per Ncode miRNA qRT-PCR kit. Five tenfold serial dilutions of let-7f specific cDNA in triplicate from uninfected A549 cells were run in parallel to calculate copy numbers. As per MIQE guidelines only standards with RSq >0.99, PCR efficiency 90–110% and slope between -3.1 to -3.4 were used to calculate copy numbers. Data is represented as fold change relative to mock from two independent experiments. Error bars represent SEM. (TIF) [file pone.0066796.s004.tif]

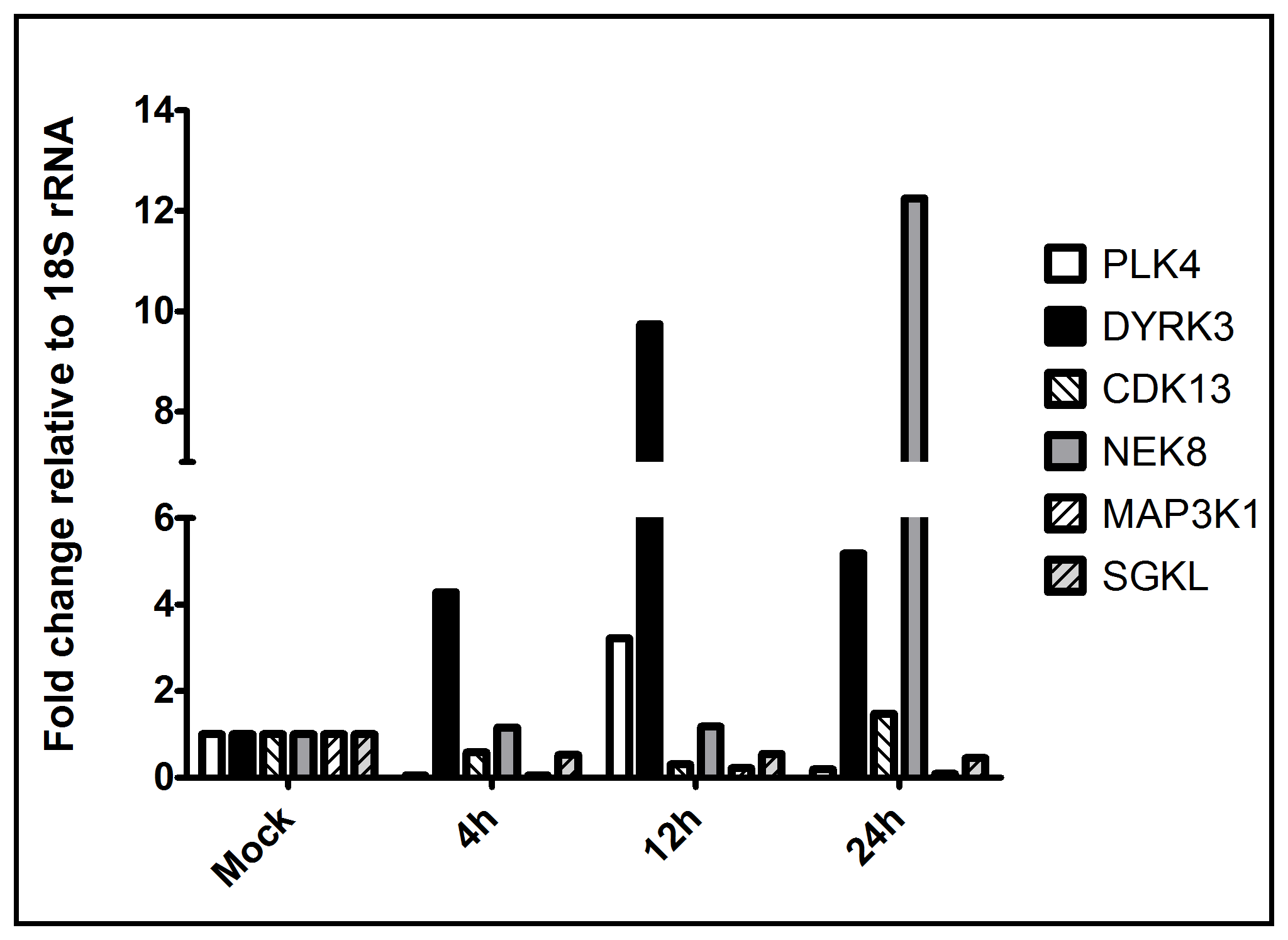

Supplement: Figure S5 — HPK expression in response to Influenza virus infection. A549 cells were infected with A/WSN/33 (MOI = 0.05) and RNA was isolated at 4 h, 12 h and 24 h post infection using Trizol as per manufacturer’s instructions. HPK expression was analyzed using gene specific qPCR primers and plotted relative to 18S rRNA expression. Data represent means from two experiments. (TIF) [file pone.0066796.s005.tif]

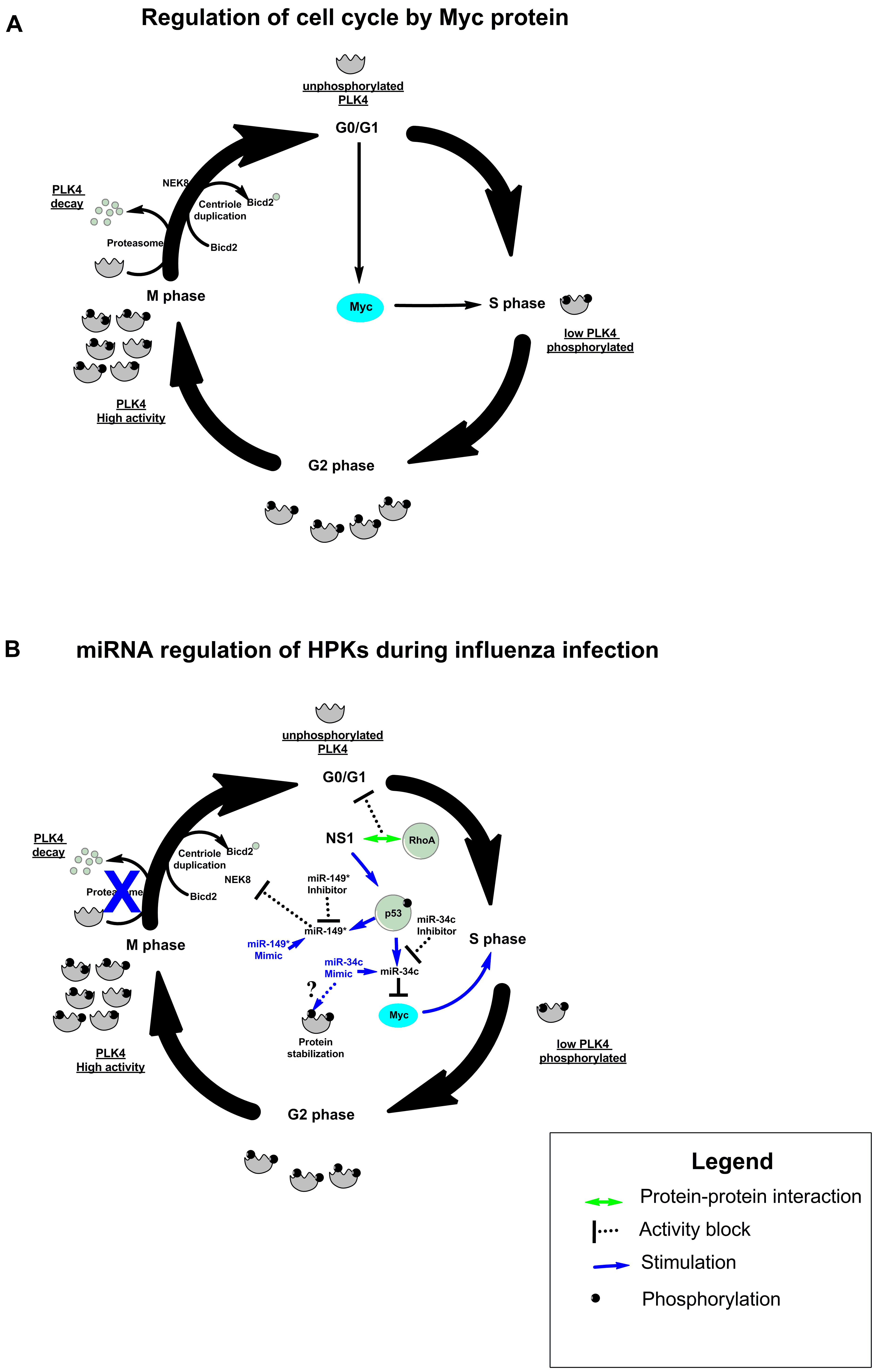

Supplement: Figure S6 — Predicted pathways of miRNA regulation of HPK expression during influenza infection. Schematic outline of cell cycle in mock infected cells. G1 to S phase transition is controlled by multiple factors of which Myc is a major regulator. Levels of PLK4 transcript and auto phosphorylated active PLK4 protein increase starting at S phase and peak at M phase. Active PLK4 targets its own degradation via proteasome mediated decay and is degraded.to complete cytokinesis and entry into G0/G1 phase. (B) Influenza (via NS1 protein) infection induces expression of p53 protein that induces miR-34c. miR-34c suppresses Myc mediated entry into S phase. Inhibition of miR-34c accelerates cell cycling and increased degradation of PLK4 protein and decreased influenza replication. miR-34c mimic arrests cells in G2/S phase by suppressing Myc leading to increased influenza replication. (TIF) [file pone.0066796.s006.tif]
