# Supplementary material for: Identification of Host Kinase Genes Required for Influenza Virus Replication and the Regulatory Role of MicroRNAs
Source: PLoS One. 2013 Jun 21;8(6):e66796. doi: 10.1371/journal.pone.0066796 (PMC3689682; doi:10.1371/journal.pone.0066796)
Supplement: Table S1 — Normalized scores, Z score analysis and cytotoxicity data for hits from host protein kinase screen. Table listing the location, siRNA target gene symbol and raw and normalized scores for all the kinases tested in this manuscript. Raw scores were normalized across the library to calculate mean score, standard deviation and Z-scores. Cytotoxicity data as measured by Toxilight assay is also shown. (DOCX) [file pone.0066796.s007.docx]

| **Supplemental Table S1. Normalized scores, Z-score analysis and cytotoxicity.** | | | | | | | | | | | | | | | | | | | |
| --- | --- | --- | --- | --- | --- | --- | --- | --- | --- | --- | --- | --- | --- | --- | --- | --- | --- | --- | --- |
| LOCATION (Plate_Well) | siRNA TARGET | RAW SCORE | | | | | | | NORMALIZED SCORE | | | | | | | MEAN | SD | Z SCORE | CYTOX |
|  |  | EXP1 | EXP2 | EXP3 | EXP4 | EXP5 | EXP6 | EXP7 | EXP1 | EXP2 | EXP3 | EXP4 | EXP5 | EXP6 | EXP7 |  |  |  |  |
| 1_A1 | NEG | 4 | 4 | 3 | 3 |  |  |  |  |  |  |  |  |  |  |  |  |  |  |
| 1_B1 | NEG | 1 | 2 | 3 | 3 |  |  |  |  |  |  |  |  |  |  |  |  |  |  |
| 1_C1 | NEG | 2 | 4 | 3 | 4 |  |  |  |  |  |  |  |  |  |  |  |  |  |  |
| 1_D1 | NEG | 3 | 2 | 3 | 4 |  |  |  |  |  |  |  |  |  |  |  |  |  |  |
| 1_E1 | NEG | 1 | 1 | 3 | 3 |  |  |  |  |  |  |  |  |  |  |  |  |  |  |
| 1_F1 | NEG | 3 | 3 | 4 | 3 |  |  |  |  |  |  |  |  |  |  |  |  |  |  |
| 1_G1 | NEG | 1 | 1 | 3 | 3 |  |  |  |  |  |  |  |  |  |  |  |  |  |  |
| 1_H1 | NEG | 3 | 3 | 3 | 3 |  |  |  | 2.25 | 2.50 | 3.13 | 3.25 |  |  |  | 2.78 |  |  | 0 |
| 1_A12 | MEK | 0 | 0 | 2 | 2 |  |  |  |  |  |  |  |  |  |  |  |  |  |  |
| 1_B12 | MEK | 0 | 0 | 2 | 1 |  |  |  |  |  |  |  |  |  |  |  |  |  |  |
| 1_C12 | MEK | 1 | 2 | 0 | 1 |  |  |  |  |  |  |  |  |  |  |  |  |  |  |
| 1_D12 | MEK | 2 | 2 | 1 | 1 |  |  |  |  |  |  |  |  |  |  |  |  |  |  |
| 1_E12 | MEK | 1 | 1 | 1 | 1 |  |  |  | 0.36 | 0.40 | 0.38 | 0.37 |  |  |  | 0.38 |  |  | 19 |
| 1_F12 | TOX | 0 | 0 | 0 | 0 |  |  |  |  |  |  |  |  |  |  |  |  |  |  |
| 1_G12 | TOX | 0 | 0 | 0 | 0 |  |  |  |  |  |  |  |  |  |  |  |  |  |  |
| 1_H12 | TOX | 0 | 0 | 0 | 0 |  |  |  | 0.00 | 0.00 | 0.00 | 0.00 |  |  |  | 0.00 |  |  | 100 |
| 1_A2 | TTK | 2 | 2 | 2 | 2 |  |  |  | 0.89 | 0.80 | 0.64 | 0.62 |  |  |  | 0.74 | 0.13 | -0.35 | 32 |
| 1_B2 | ACVRL1 | 2 | 2 | 3 | 3 |  |  |  | 0.89 | 0.80 | 0.96 | 0.92 |  |  |  | 0.89 | 0.07 | 0.12 | 26 |
| 1_C2 | ALS2CR2 | 0 | 0 | 4 | 4 |  |  |  | 0.00 | 0.00 | 1.28 | 1.23 |  |  |  | 0.63 | 0.73 | -0.68 | 23 |
| 1_D2 | CDC2 | 3 | 1 | 3 | 3 |  |  |  | 1.33 | 0.40 | 0.96 | 0.92 |  |  |  | 0.90 | 0.38 | 0.16 | 12 |
| 1_E2 | PRKAG3 | 0 | 0 | 0 | 0 |  |  |  | 0.00 | 0.00 | 0.00 | 0.00 |  |  |  | 0.00 | 0.00 | -2.59 | 4 |
| 1_F2 | TEK | 4 | 3 | 5 | 4 |  |  |  | 1.78 | 1.20 | 1.60 | 1.23 |  |  |  | 1.45 | 0.28 | 1.82 | 27 |
| 1_G2 | ATM | 2 | 1 | 3 | 2 |  |  |  | 0.89 | 0.40 | 0.96 | 0.62 |  |  |  | 0.72 | 0.26 | -0.41 | 40 |
| 1_H2 | RFP | 1 | 1 | 0 | 0 |  |  |  | 0.44 | 0.40 | 0.00 | 0.00 |  |  |  | 0.21 | 0.24 | -1.95 | 35 |
| 1_A3 | SYK | 0 | 0 | 0 | 0 |  |  |  | 0.00 | 0.00 | 0.00 | 0.00 |  |  |  | 0.00 | 0.00 | -2.59 | 38 |
| 1_B3 | LYN | 0 | 2 | 3 | 4 |  |  |  | 0.00 | 0.80 | 0.96 | 1.23 |  |  |  | 0.75 | 0.53 | -0.32 | 31 |
| 1_C3 | CARKL | 0 | 0 | 0 | 0 |  |  |  | 0.00 | 0.00 | 0.00 | 0.00 |  |  |  | 0.00 | 0.00 | -2.59 | 30 |
| 1_D3 | PIM2 | 1 | 2 | 2 | 4 |  |  |  | 0.44 | 0.80 | 0.64 | 1.23 |  |  |  | 0.78 | 0.33 | -0.22 | 26 |
| 1_E3 | GRK5 | 3 | 3 | 0 | 1 |  |  |  | 1.33 | 1.20 | 0.00 | 0.31 |  |  |  | 0.71 | 0.66 | -0.43 | 31 |
| 1_F3 | PANK1 | 4 | 4 | 3 | 2 |  |  |  | 1.78 | 1.60 | 0.96 | 0.62 |  |  |  | 1.24 | 0.54 | 1.17 | 31 |
| 1_G3 | PHKA2 | 2 | 1 | 4 | 4 |  |  |  | 0.89 | 0.40 | 1.28 | 1.23 |  |  |  | 0.95 | 0.41 | 0.30 | 8 |
| 1_H3 | PIP5KL1 | 2 | 2 | 3 | 3 |  |  |  | 0.89 | 0.80 | 0.96 | 0.92 |  |  |  | 0.89 | 0.07 | 0.12 | 34 |
| 1_A4 | EPHA8 | 1 | 1 | 1 | 1 |  |  |  | 0.44 | 0.40 | 0.32 | 0.31 |  |  |  | 0.37 | 0.07 | -1.47 | 16 |
| 1_B4 | PAPSS1 | 2 | 1 | 3 | 3 |  |  |  | 0.89 | 0.40 | 0.96 | 0.92 |  |  |  | 0.79 | 0.26 | -0.18 | 21 |
| 1_C4 | ADK | 0 | 0 | 0 | 0 |  |  |  | 0.00 | 0.00 | 0.00 | 0.00 |  |  |  | 0.00 | 0.00 | -2.59 | 12 |
| 1_D4 | LRRK2 | 0 | 1 | 0 | 0 |  |  |  | 0.00 | 0.40 | 0.00 | 0.00 |  |  |  | 0.10 | 0.20 | -2.29 | 31 |
| 1_E4 | LIMK1 | 2 | 2 | 2 | 3 |  |  |  | 0.89 | 0.80 | 0.64 | 0.92 |  |  |  | 0.81 | 0.13 | -0.12 | 35 |
| 1_F4 | CSNK1G3 | 4 | 4 | 4 | 4 |  |  |  | 1.78 | 1.60 | 1.28 | 1.23 |  |  |  | 1.47 | 0.26 | 1.88 | 34 |
| 1_G4 | MULK | 0 | 0 | 2 | 2 |  |  |  | 0.00 | 0.00 | 0.64 | 0.62 |  |  |  | 0.31 | 0.36 | -1.64 | 13 |
| 1_H4 | NEK5 | 2 | 1 | 5 | 4 |  |  |  | 0.89 | 0.40 | 1.60 | 1.23 |  |  |  | 1.03 | 0.51 | 0.54 | 27 |
| 1_A5 | DMPK | 1 | 1 | 3 | 4 |  |  |  | 0.44 | 0.40 | 0.96 | 1.23 |  |  |  | 0.76 | 0.40 | -0.28 | 48 |
| 1_B5 | MAPK4 | 2 | 1 | 3 | 3 |  |  |  | 0.89 | 0.40 | 0.96 | 0.92 |  |  |  | 0.79 | 0.26 | -0.18 | 20 |
| 1_C5 | ABL2 | 2 | 3 | 2 | 1 |  |  |  | 0.89 | 1.20 | 0.64 | 0.31 |  |  |  | 0.76 | 0.38 | -0.28 | 21 |
| 1_D5 | HK2 | 0 | 0 | 0 | 0 |  |  |  | 0.00 | 0.00 | 0.00 | 0.00 |  |  |  | 0.00 | 0.00 | -2.59 | 20 |
| 1_E5 | UMPK | 2 | 2 | 3 | 3 |  |  |  | 0.89 | 0.80 | 0.96 | 0.92 |  |  |  | 0.89 | 0.07 | 0.12 | 21 |
| 1_F5 | CKS2 | 2 | 3 | 3 | 3 |  |  |  | 0.89 | 1.20 | 0.96 | 0.92 |  |  |  | 0.99 | 0.14 | 0.43 | 23 |
| 1_G5 | GRK6 | 3 | 4 | 4 | 4 |  |  |  | 1.33 | 1.60 | 1.28 | 1.23 |  |  |  | 1.36 | 0.16 | 1.55 | 31 |
| 1_H5 | PLK2 | 1 | 2 | 2 | 1 |  |  |  | 0.44 | 0.80 | 0.64 | 0.31 |  |  |  | 0.55 | 0.22 | -0.93 | 15 |
| 1_A6 | COASY | 2 | 2 | 1 | 1 |  |  |  | 0.89 | 0.80 | 0.32 | 0.31 |  |  |  | 0.58 | 0.31 | -0.83 | 33 |
| 1_B6 | MASTL | 3 | 2 | 2 | 4 |  |  |  | 1.33 | 0.80 | 0.64 | 1.23 |  |  |  | 1.00 | 0.33 | 0.45 | 83 |
| 1_C6 | PLK1 | 0 | 0 | 0 | 0 |  |  |  | 0.00 | 0.00 | 0.00 | 0.00 |  |  |  | 0.00 | 0.00 | -2.59 | 83 |
| 1_D6 | FN3K | 2 | 1 | 3 | 3 |  |  |  | 0.89 | 0.40 | 0.96 | 0.92 |  |  |  | 0.79 | 0.26 | -0.18 | 21 |
| 1_E6 | C7ORF2 | 2 | 2 | 3 | 4 |  |  |  | 0.89 | 0.80 | 0.96 | 1.23 |  |  |  | 0.97 | 0.19 | 0.36 | 18 |
| 1_F6 | PHKG2 | 0 | 0 | 1 | 1 |  |  |  | 0.00 | 0.00 | 0.32 | 0.31 |  |  |  | 0.16 | 0.18 | -2.11 | 36 |
| 1_G6 | BMPR1B | 2 | 3 | 3 | 2 |  |  |  | 0.89 | 1.20 | 0.96 | 0.62 |  |  |  | 0.92 | 0.24 | 0.19 | 40 |
| 1_H6 | CSNK1G2 | 1 | 1 | 3 | 2 |  |  |  | 0.44 | 0.40 | 0.96 | 0.62 |  |  |  | 0.60 | 0.25 | -0.75 | 43 |
| 1_A7 | MYO3B | 3 | 4 | 2 | 1 |  |  |  | 1.33 | 1.60 | 0.64 | 0.31 |  |  |  | 0.97 | 0.60 | 0.36 | 42 |
| 1_B7 | TRIB1 | 2 | 2 | 3 | 3 |  |  |  | 0.89 | 0.80 | 0.96 | 0.92 |  |  |  | 0.89 | 0.07 | 0.12 | 28 |
| 1_C7 | MINK | 0 | 0 | 0 | 0 |  |  |  | 0.00 | 0.00 | 0.00 | 0.00 |  |  |  | 0.00 | 0.00 | -2.59 | 37 |
| 1_D7 | CRIM1 | 2 | 2 | 3 | 3 |  |  |  | 0.89 | 0.80 | 0.96 | 0.92 |  |  |  | 0.89 | 0.07 | 0.12 | 46 |
| 1_E7 | KSR | 0 | 0 | 2 | 2 |  |  |  | 0.00 | 0.00 | 0.64 | 0.62 |  |  |  | 0.31 | 0.36 | -1.64 | 11 |
| 1_F7 | MAPK7 | 4 | 3 | 4 | 3 |  |  |  | 1.78 | 1.20 | 1.28 | 0.92 |  |  |  | 1.30 | 0.36 | 1.35 | 28 |
| 1_G7 | STK33 | 2 | 2 | 4 | 4 |  |  |  | 0.89 | 0.80 | 1.28 | 1.23 |  |  |  | 1.05 | 0.24 | 0.60 | 33 |
| 1_H7 | MARK2 | 2 | 3 | 2 | 3 |  |  |  | 0.89 | 1.20 | 0.64 | 0.92 |  |  |  | 0.91 | 0.23 | 0.18 | 25 |
| 1_A8 | PRKWNK2 | 2 | 4 | 4 | 4 |  |  |  | 0.89 | 1.60 | 1.28 | 1.23 |  |  |  | 1.25 | 0.29 | 1.21 | 32 |
| 1_B8 | BRD3 | 1 | 2 | 3 | 3 |  |  |  | 0.44 | 0.80 | 0.96 | 0.92 |  |  |  | 0.78 | 0.24 | -0.21 | 26 |
| 1_C8 | CDKL3 | 2 | 0 | 3 | 3 |  |  |  | 0.89 | 0.00 | 0.96 | 0.92 |  |  |  | 0.69 | 0.46 | -0.48 | 28 |
| 1_D8 | PIK4CA | 1 | 2 | 0 | 1 |  |  |  | 0.44 | 0.80 | 0.00 | 0.31 |  |  |  | 0.39 | 0.33 | -1.41 | 30 |
| 1_E8 | MAP4K4 | 4 | 0 | 4 | 5 |  |  |  | 1.78 | 0.00 | 1.28 | 1.54 |  |  |  | 1.15 | 0.79 | 0.90 | 31 |
| 1_F8 | KCNH8 | 1 | 3 | 3 | 3 |  |  |  | 0.44 | 1.20 | 0.96 | 0.92 |  |  |  | 0.88 | 0.32 | 0.09 | 42 |
| 1_G8 | MIDORI | 2 | 2 | 1 | 1 |  |  |  | 0.89 | 0.80 | 0.32 | 0.31 |  |  |  | 0.58 | 0.31 | -0.83 | 32 |
| 1_H8 | TNIK | 1 | 3 | 0 | 0 |  |  |  | 0.44 | 1.20 | 0.00 | 0.00 |  |  |  | 0.41 | 0.57 | -1.34 | 42 |
| 1_A9 | PTK2 | 0 | 3 | 0 | 0 |  |  |  | 0.00 | 1.20 | 0.00 | 0.00 |  |  |  | 0.30 | 0.60 | -1.68 | 27 |
| 1_B9 | FLJ34389 | 0 | 1 | 1 | 1 |  |  |  | 0.00 | 0.40 | 0.32 | 0.31 |  |  |  | 0.26 | 0.18 | -1.81 | 38 |
| 1_C9 | MAP3K14 | 0 | 2 | 2 | 2 |  |  |  | 0.00 | 0.80 | 0.64 | 0.62 |  |  |  | 0.51 | 0.35 | -1.03 | 38 |
| 1_D9 | SSTK | 2 | 1 | 0 | 0 |  |  |  | 0.89 | 0.40 | 0.00 | 0.00 |  |  |  | 0.32 | 0.42 | -1.61 | 45 |
| 1_E9 | PRKCN | 1 | 3 | 3 | 2 |  |  |  | 0.44 | 1.20 | 0.96 | 0.62 |  |  |  | 0.80 | 0.34 | -0.14 | 33 |
| 1_F9 | CAMK2D | 1 | 1 | 1 | 0 |  |  |  | 0.44 | 0.40 | 0.32 | 0.00 |  |  |  | 0.29 | 0.20 | -1.71 | 23 |
| 1_G9 | RNASEL | 0 | 2 | 1 | 2 |  |  |  | 0.00 | 0.80 | 0.32 | 0.62 |  |  |  | 0.43 | 0.35 | -1.27 | 46 |
| 1_H9 | CALM3 | 2 | 0 | 3 | 3 |  |  |  | 0.89 | 0.00 | 0.96 | 0.92 |  |  |  | 0.69 | 0.46 | -0.48 | 33 |
| 1_A10 | FER | 1 | 0 | 3 | 3 |  |  |  | 0.44 | 0.00 | 0.96 | 0.92 |  |  |  | 0.58 | 0.45 | -0.82 | 32 |
| 1_B10 | HK1 | 2 | 0 | 2 | 2 |  |  |  | 0.89 | 0.00 | 0.64 | 0.62 |  |  |  | 0.54 | 0.38 | -0.96 | 40 |
| 1_C10 | MAP3K7 | 1 | 0 | 2 | 4 |  |  |  | 0.44 | 0.00 | 0.64 | 1.23 |  |  |  | 0.58 | 0.51 | -0.83 | 49 |
| 1_D10 | TAO1 | 3 | 2 | 2 | 2 |  |  |  | 1.33 | 0.80 | 0.64 | 0.62 |  |  |  | 0.85 | 0.33 | -0.02 | 24 |
| 1_E10 | TTBK1 | 0 | 1 | 3 | 2 |  |  |  | 0.00 | 0.40 | 0.96 | 0.62 |  |  |  | 0.49 | 0.40 | -1.09 | 41 |
| 1_F10 | MAP4K5 | 1 | 0 | 2 | 2 |  |  |  | 0.44 | 0.00 | 0.64 | 0.62 |  |  |  | 0.42 | 0.30 | -1.30 | 40 |
| 1_G10 | STK16 | 2 | 1 | 3 | 2 |  |  |  | 0.89 | 0.40 | 0.96 | 0.62 |  |  |  | 0.72 | 0.26 | -0.41 | 38 |
| 1_H10 | PKM2 | 2 | 1 | 3 | 3 |  |  |  | 0.89 | 0.40 | 0.96 | 0.92 |  |  |  | 0.79 | 0.26 | -0.18 | 36 |
| 1_A11 | CAMK2B | 1 | 1 | 3 | 3 |  |  |  | 0.44 | 0.40 | 0.96 | 0.92 |  |  |  | 0.68 | 0.30 | -0.52 | 28 |
| 1_B11 | THNSL1 | 1 | 1 | 2 | 2 |  |  |  | 0.44 | 0.40 | 0.64 | 0.62 |  |  |  | 0.52 | 0.12 | -1.00 | 20 |
| 1_C11 | CKB | 1 | 1 | 2 | 2 |  |  |  | 0.44 | 0.40 | 0.64 | 0.62 |  |  |  | 0.52 | 0.12 | -1.00 | 23 |
| 1_D11 | NME3 | 0 | 1 | 0 | 0 |  |  |  | 0.00 | 0.40 | 0.00 | 0.00 |  |  |  | 0.10 | 0.20 | -2.29 | 23 |
| 1_E11 | ROCK1 | 1 | 2 | 2 | 1 |  |  |  | 0.44 | 0.80 | 0.64 | 0.31 |  |  |  | 0.55 | 0.22 | -0.93 | 19 |
| 1_F11 | PKN3 | 0 | 0 | 0 | 0 |  |  |  | 0.00 | 0.00 | 0.00 | 0.00 |  |  |  | 0.00 | 0.00 | -2.59 | 18 |
| 1_G11 | GRK1 | 2 | 2 | 4 | 4 |  |  |  | 0.89 | 0.80 | 1.28 | 1.23 |  |  |  | 1.05 | 0.24 | 0.60 | 28 |
| 1_H11 | CDKL5 | 3 | 4 | 2 | 2 |  |  |  | 1.33 | 1.60 | 0.64 | 0.62 |  |  |  | 1.05 | 0.50 | 0.59 | 11 |
| 2_A1 | NEG | 4 | 4 | 3 | 3 | 3 |  |  |  |  |  |  |  |  |  |  |  |  |  |
| 2_B1 | NEG | 3 | 3 | 3 | 3 | 4 |  |  |  |  |  |  |  |  |  |  |  |  |  |
| 2_C1 | NEG | 3 | 2 | 3 | 3 | 3 |  |  |  |  |  |  |  |  |  |  |  |  |  |
| 2_D1 | NEG | 3 | 4 | 3 | 4 | 4 |  |  |  |  |  |  |  |  |  |  |  |  |  |
| 2_E1 | NEG | 3 | 3 | 3 | 3 |  |  |  |  |  |  |  |  |  |  |  |  |  |  |
| 2_F1 | NEG | 3 | 2 | 3 | 3 |  |  |  |  |  |  |  |  |  |  |  |  |  |  |
| 2_G1 | NEG | 3 | 4 | 3 | 4 |  |  |  |  |  |  |  |  |  |  |  |  |  |  |
| 2_H1 | NEG | 4 | 4 | 3 | 4 |  |  |  | 3.25 | 3.25 | 3.00 | 3.38 | 3.50 |  |  | 3.28 |  |  | 0 |
| 2_A12 | MEK | 1 | 1 | 2 | 1 | 3 |  |  |  |  |  |  |  |  |  |  |  |  |  |
| 2_B12 | MEK | 1 | 3 | 0 | 0 | 0 |  |  |  |  |  |  |  |  |  |  |  |  |  |
| 2_C12 | MEK | 1 | 2 | 0 | 2 | 2 |  |  |  |  |  |  |  |  |  |  |  |  |  |
| 2_D12 | MEK | 0 | 0 | 1 | 1 |  |  |  |  |  |  |  |  |  |  |  |  |  |  |
| 2_E12 | MEK | 2 | 2 | 2 | 2 |  |  |  | 0.31 | 0.49 | 0.33 | 0.36 | 0.48 |  |  | 0.39 |  |  | 0 |
| 2_F12 | TOX | 0 | 0 | 0 | 0 | 0 |  |  |  |  |  |  |  |  |  |  |  |  |  |
| 2_G12 | TOX | 0 | 0 | 0 | 0 |  |  |  |  |  |  |  |  |  |  |  |  |  |  |
| 2_H12 | TOX | 0 | 0 | 0 | 0 |  |  |  | 0.00 | 0.00 | 0.00 | 0.00 | 0.00 |  |  | 0.00 |  |  | 100 |
| 2_A2 | PIK3CA | 1 | 2 | 2 | 2 |  |  |  | 0.31 | 0.62 | 0.67 | 0.59 |  |  |  | 0.55 | 0.16 | -0.93 | 0 |
| 2_B2 | FLJ32685 | 3 | 3 | 4 | 5 |  |  |  | 0.92 | 0.92 | 1.33 | 1.48 |  |  |  | 1.17 | 0.29 | 0.95 | 0 |
| 2_C2 | PAPSS2 | 3 | 4 | 4 | 4 |  |  |  | 0.92 | 1.23 | 1.33 | 1.19 |  |  |  | 1.17 | 0.17 | 0.96 | 0 |
| 2_D2 | KIAA1811 | 2 | 3 | 4 | 4 |  |  |  | 0.62 | 0.92 | 1.33 | 1.19 |  |  |  | 1.01 | 0.32 | 0.49 | 3 |
| 2_E2 | RIOK2 | 1 | 2 | 2 | 3 |  |  |  | 0.31 | 0.62 | 0.67 | 0.89 |  |  |  | 0.62 | 0.24 | -0.71 | 0 |
| 2_F2 | STK25 | 4 | 4 | 5 | 5 |  |  |  | 1.23 | 1.23 | 1.67 | 1.48 |  |  |  | 1.40 | 0.21 | 1.67 | 1 |
| 2_G2 | PRKCL1 | 2 | 2 | 3 | 2 |  |  |  | 0.62 | 0.62 | 1.00 | 0.59 |  |  |  | 0.71 | 0.20 | -0.45 | 2 |
| 2_H2 | TRIO | 3 | 3 | 4 | 4 |  |  |  | 0.92 | 0.92 | 1.33 | 1.19 |  |  |  | 1.09 | 0.20 | 0.73 | 3 |
| 2_A3 | KIAA1804 | 3 | 4 | 0 | 0 |  |  |  | 0.92 | 1.23 | 0.00 | 0.00 |  |  |  | 0.54 | 0.63 | -0.95 | 6 |
| 2_B3 | ITPKC | 4 | 4 | 3 | 3 |  |  |  | 1.23 | 1.23 | 1.00 | 0.89 |  |  |  | 1.09 | 0.17 | 0.72 | 0 |
| 2_C3 | RPS6KB2 | 3 | 2 | 3 | 2 |  |  |  | 0.92 | 0.62 | 1.00 | 0.59 |  |  |  | 0.78 | 0.21 | -0.21 | 0 |
| 2_D3 | PDK2 | 0 | 1 | 1 | 0 | 1 |  |  | 0.00 | 0.31 | 0.33 | 0.00 | 0.29 |  |  | 0.19 | 0.17 | -2.03 | 1 |
| 2_E3 | FRAP1 | 4 | 3 | 4 | 4 |  |  |  | 1.23 | 0.92 | 1.33 | 1.19 |  |  |  | 1.17 | 0.17 | 0.96 | 0 |
| 2_F3 | CDC7 | 3 | 3 | 3 | 4 |  |  |  | 0.92 | 0.92 | 1.00 | 1.19 |  |  |  | 1.01 | 0.12 | 0.47 | 0 |
| 2_G3 | RIPK2 | 0 | 1 | 1 | 2 |  |  |  | 0.00 | 0.31 | 0.33 | 0.59 |  |  |  | 0.31 | 0.24 | -1.65 | 0 |
| 2_H3 | ETNK1 | 3 | 3 | 1 | 2 |  |  |  | 0.92 | 0.92 | 0.33 | 0.59 |  |  |  | 0.69 | 0.29 | -0.48 | 4 |
| 2_A4 | NAGK | 4 | 4 | 3 | 3 |  |  |  | 1.23 | 1.23 | 1.00 | 0.89 |  |  |  | 1.09 | 0.17 | 0.72 | 0 |
| 2_B4 | EPHA5 | 1 | 1 | 3 | 2 |  |  |  | 0.31 | 0.31 | 1.00 | 0.59 |  |  |  | 0.55 | 0.33 | -0.91 | 0 |
| 2_C4 | PFTK1 | 2 | 2 | 4 | 4 |  |  |  | 0.62 | 0.62 | 1.33 | 1.19 |  |  |  | 0.94 | 0.38 | 0.26 | 0 |
| 2_D4 | MYLK2 | 1 | 1 | 0 | 1 | 0 |  |  | 0.31 | 0.31 | 0.00 | 0.30 | 0.00 |  |  | 0.18 | 0.17 | -2.04 | 0 |
| 2_E4 | PDGFRL | 1 | 2 | 1 | 6 |  |  |  | 0.31 | 0.62 | 0.33 | 1.78 |  |  |  | 0.76 | 0.69 | -0.29 | 1 |
| 2_F4 | CAMK2G | 4 | 4 | 5 | 5 |  |  |  | 1.23 | 1.23 | 1.67 | 1.48 |  |  |  | 1.40 | 0.21 | 1.67 | 1 |
| 2_G4 | CKMT1B | 1 | 1 | 1 | 2 |  |  |  | 0.31 | 0.31 | 0.33 | 0.59 |  |  |  | 0.39 | 0.14 | -1.42 | 3 |
| 2_H4 | ERK8 | 2 | 3 | 0 | 0 |  |  |  | 0.62 | 0.92 | 0.00 | 0.00 |  |  |  | 0.38 | 0.46 | -1.42 | 0 |
| 2_A5 | IRAK1 | 3 | 4 | 2 | 3 |  |  |  | 0.92 | 1.23 | 0.67 | 0.89 |  |  |  | 0.93 | 0.23 | 0.23 | 0 |
| 2_B5 | MLCK | 4 | 4 | 4 | 4 |  |  |  | 1.23 | 1.23 | 1.33 | 1.19 |  |  |  | 1.25 | 0.06 | 1.19 | 0 |
| 2_C5 | NME5 | 4 | 4 | 4 | 4 |  |  |  | 1.23 | 1.23 | 1.33 | 1.19 |  |  |  | 1.25 | 0.06 | 1.19 | 0 |
| 2_D5 | NEK11 | 2 | 1 | 2 | 3 |  |  |  | 0.62 | 0.31 | 0.67 | 0.89 |  |  |  | 0.62 | 0.24 | -0.71 | 0 |
| 2_E5 | AK7 | 1 | 1 | 4 | 8 |  |  |  | 0.31 | 0.31 | 1.33 | 2.37 |  |  |  | 1.08 | 0.99 | 0.69 | 0 |
| 2_F5 | URKL1 | 2 | 3 | 3 | 4 |  |  |  | 0.62 | 0.92 | 1.00 | 1.19 |  |  |  | 0.93 | 0.24 | 0.24 | 0 |
| 2_G5 | MKNK2 | 4 | 4 | 2 | 1 |  |  |  | 1.23 | 1.23 | 0.67 | 0.30 |  |  |  | 0.86 | 0.46 | 0.01 | 4 |
| 2_H5 | ADP-GK | 3 | 4 | 4 | 5 |  |  |  | 0.92 | 1.23 | 1.33 | 1.48 |  |  |  | 1.24 | 0.24 | 1.19 | 3 |
| 2_A6 | EEF2K | 4 | 3 | 0 | 0 |  |  |  | 1.23 | 0.92 | 0.00 | 0.00 |  |  |  | 0.54 | 0.63 | -0.95 | 0 |
| 2_B6 | PSKH1 | 0 | 0 | 3 | 2 |  |  |  | 0.00 | 0.00 | 1.00 | 0.59 |  |  |  | 0.40 | 0.49 | -1.38 | 2 |
| 2_C6 | SRC | 2 | 1 | 0 | 0 |  |  |  | 0.62 | 0.31 | 0.00 | 0.00 |  |  |  | 0.23 | 0.29 | -1.89 | 0 |
| 2_D6 | KIAA1361 | 4 | 4 | 4 | 4 |  |  |  | 1.23 | 1.23 | 1.33 | 1.19 |  |  |  | 1.25 | 0.06 | 1.19 | 0 |
| 2_E6 | EPHA10 | 2 | 1 | 3 | 8 |  |  |  | 0.62 | 0.31 | 1.00 | 2.37 |  |  |  | 1.07 | 0.91 | 0.67 | 2 |
| 2_F6 | MAP3K9 | 4 | 4 | 5 | 4 |  |  |  | 1.23 | 1.23 | 1.67 | 1.19 |  |  |  | 1.33 | 0.23 | 1.45 | 0 |
| 2_G6 | TNNI3K | 4 | 4 | 3 | 3 |  |  |  | 1.23 | 1.23 | 1.00 | 0.89 |  |  |  | 1.09 | 0.17 | 0.72 | 1 |
| 2_H6 | PIK3CG | 3 | 3 | 0 | 0 |  |  |  | 0.92 | 0.92 | 0.00 | 0.00 |  |  |  | 0.46 | 0.53 | -1.19 | 5 |
| 2_A7 | ANKK1 | 2 | 3 | 3 | 3 |  |  |  | 0.62 | 0.92 | 1.00 | 0.89 |  |  |  | 0.86 | 0.17 | 0.01 | 0 |
| 2_B7 | AURKC | 3 | 3 | 1 | 1 |  |  |  | 0.92 | 0.92 | 0.33 | 0.30 |  |  |  | 0.62 | 0.35 | -0.71 | 1 |
| 2_C7 | NEK4 | 4 | 4 | 4 | 4 |  |  |  | 1.23 | 1.23 | 1.33 | 1.19 |  |  |  | 1.25 | 0.06 | 1.19 | 0 |
| 2_D7 | SNF1LK | 4 | 4 | 4 | 4 |  |  |  | 1.23 | 1.23 | 1.33 | 1.19 |  |  |  | 1.25 | 0.06 | 1.19 | 0 |
| 2_E7 | STK17B | 3 | 2 | 3 | 4 |  |  |  | 0.92 | 0.62 | 1.00 | 1.19 |  |  |  | 0.93 | 0.24 | 0.24 | 0 |
| 2_F7 | GUCY2D | 0 | 0 | 2 | 2 |  |  |  | 0.00 | 0.00 | 0.67 | 0.59 |  |  |  | 0.31 | 0.36 | -1.63 | 3 |
| 2_G7 | ALS2CR7 | 4 | 4 | 5 | 4 |  |  |  | 1.23 | 1.23 | 1.67 | 1.19 |  |  |  | 1.33 | 0.23 | 1.45 | 0 |
| 2_H7 | FGFR3 | 3 | 3 | 1 | 2 |  |  |  | 0.92 | 0.92 | 0.33 | 0.59 |  |  |  | 0.69 | 0.29 | -0.48 | 6 |
| 2_A8 | MAP3K5 | 3 | 3 | 3 | 3 |  |  |  | 0.92 | 0.92 | 1.00 | 0.89 |  |  |  | 0.93 | 0.05 | 0.25 | 0 |
| 2_B8 | PRKCE | 1 | 3 | 1 | 1 |  |  |  | 0.31 | 0.92 | 0.33 | 0.30 |  |  |  | 0.47 | 0.31 | -1.18 | 0 |
| 2_C8 | CSNK1G1 | 3 | 4 | 4 | 4 |  |  |  | 0.92 | 1.23 | 1.33 | 1.19 |  |  |  | 1.17 | 0.17 | 0.96 | 0 |
| 2_D8 | PTK9 | 4 | 4 | 3 | 3 |  |  |  | 1.23 | 1.23 | 1.00 | 0.89 |  |  |  | 1.09 | 0.17 | 0.72 | 0 |
| 2_E8 | NPR2 | 4 | 2 | 8 | 8 | 6 |  |  | 1.23 | 0.62 | 2.67 | 2.37 | 1.71 |  |  | 1.72 | 0.83 | 2.64 | 1 |
| 2_F8 | CAMKK2 | 3 | 0 | 2 | 3 |  |  |  | 0.92 | 0.00 | 0.67 | 0.89 |  |  |  | 0.62 | 0.43 | -0.71 | 0 |
| 2_G8 | HCK | 4 | 4 | 4 | 6 |  |  |  | 1.23 | 1.23 | 1.33 | 1.78 |  |  |  | 1.39 | 0.26 | 1.64 | 2 |
| 2_H8 | IRAK4 | 4 | 3 | 4 | 4 |  |  |  | 1.23 | 0.92 | 1.33 | 1.19 |  |  |  | 1.17 | 0.17 | 0.96 | 3 |
| 2_A9 | PRKAA1 | 4 | 2 | 0 | 0 |  |  |  | 1.23 | 0.62 | 0.00 | 0.00 |  |  |  | 0.46 | 0.59 | -1.19 | 0 |
| 2_B9 | AK1 | 3 | 2 | 4 | 4 |  |  |  | 0.92 | 0.62 | 1.33 | 1.19 |  |  |  | 1.01 | 0.32 | 0.49 | 0 |
| 2_C9 | RIOK1 | 3 | 3 | 0 | 0 |  |  |  | 0.92 | 0.92 | 0.00 | 0.00 |  |  |  | 0.46 | 0.53 | -1.19 | 0 |
| 2_D9 | LATS1 | 3 | 3 | 3 | 4 |  |  |  | 0.92 | 0.92 | 1.00 | 1.19 |  |  |  | 1.01 | 0.12 | 0.47 | 0 |
| 2_E9 | GOLGA5 | 1 | 4 | 4 | 3 |  |  |  | 0.31 | 1.23 | 1.33 | 0.89 |  |  |  | 0.94 | 0.46 | 0.27 | 4 |
| 2_F9 | KIAA1639 | 3 | 3 | 3 | 4 |  |  |  | 0.92 | 0.92 | 1.00 | 1.19 |  |  |  | 1.01 | 0.12 | 0.47 | 2 |
| 2_G9 | CKS1B | 4 | 4 | 3 | 5 |  |  |  | 1.23 | 1.23 | 1.00 | 1.48 |  |  |  | 1.24 | 0.20 | 1.17 | 15 |
| 2_H9 | PCTK3 | 4 | 4 | 3 | 5 |  |  |  | 1.23 | 1.23 | 1.00 | 1.48 |  |  |  | 1.24 | 0.20 | 1.17 | 4 |
| 2_A10 | RPS6KA4 | 3 | 4 | 2 | 4 |  |  |  | 0.92 | 1.23 | 0.67 | 1.19 |  |  |  | 1.00 | 0.26 | 0.45 | 0 |
| 2_B10 | TRPM6 | 3 | 3 | 2 | 2 |  |  |  | 0.92 | 0.92 | 0.67 | 0.59 |  |  |  | 0.78 | 0.17 | -0.23 | 0 |
| 2_C10 | AKT1 | 3 | 2 | 4 | 3 |  |  |  | 0.92 | 0.62 | 1.33 | 0.89 |  |  |  | 0.94 | 0.30 | 0.27 | 0 |
| 2_D10 | PFKP | 4 | 2 | 4 | 3 |  |  |  | 1.23 | 0.62 | 1.33 | 0.89 |  |  |  | 1.02 | 0.33 | 0.50 | 6 |
| 2_E10 | STK24 | 3 | 1 | 2 | 3 |  |  |  | 0.92 | 0.31 | 0.67 | 0.89 |  |  |  | 0.70 | 0.28 | -0.47 | 7 |
| 2_F10 | CAMKIINALPHA | 3 | 2 | 3 | 3 |  |  |  | 0.92 | 0.62 | 1.00 | 0.89 |  |  |  | 0.86 | 0.17 | 0.01 | 8 |
| 2_G10 | BMP2K | 3 | 3 | 1 | 0 |  |  |  | 0.92 | 0.92 | 0.33 | 0.00 |  |  |  | 0.54 | 0.46 | -0.93 | 6 |
| 2_H10 | SGK2 | 4 | 4 | 3 | 4 |  |  |  | 1.23 | 1.23 | 1.00 | 1.19 |  |  |  | 1.16 | 0.11 | 0.94 | 3 |
| 2_A11 | CALM1 | 3 | 4 | 5 | 4 |  |  |  | 0.92 | 1.23 | 1.67 | 1.19 |  |  |  | 1.25 | 0.31 | 1.21 | 0 |
| 2_B11 | CLK1 | 2 | 1 | 1 | 1 |  |  |  | 0.62 | 0.31 | 0.33 | 0.30 |  |  |  | 0.39 | 0.15 | -1.41 | 0 |
| 2_C11 | SNRK | 3 | 3 | 2 | 2 |  |  |  | 0.92 | 0.92 | 0.67 | 0.59 |  |  |  | 0.78 | 0.17 | -0.23 | 0 |
| 2_D11 | PRKG1 | 3 | 3 | 4 | 4 |  |  |  | 0.92 | 0.92 | 1.33 | 1.19 |  |  |  | 1.09 | 0.20 | 0.73 | 0 |
| 2_E11 | DGKB | 3 | 3 | 2 | 4 |  |  |  | 0.92 | 0.92 | 0.67 | 1.19 |  |  |  | 0.92 | 0.21 | 0.22 | 4 |
| 2_F11 | KIT | 4 | 3 | 5 | 4 |  |  |  | 1.23 | 0.92 | 1.67 | 1.19 |  |  |  | 1.25 | 0.31 | 1.21 | 1 |
| 2_G11 | PHKB | 3 | 3 | 5 | 4 |  |  |  | 0.92 | 0.92 | 1.67 | 1.19 |  |  |  | 1.17 | 0.35 | 0.98 | 0 |
| 2_H11 | DYRK4 | 4 | 4 | 3 | 4 |  |  |  | 1.23 | 1.23 | 1.00 | 1.19 |  |  |  | 1.16 | 0.11 | 0.94 | 5 |
| 3_A1 | NEG | 4 | 4 | 3 | 3 | 2 | 2 | 3 |  |  |  |  |  |  |  |  |  |  |  |
| 3_B1 | NEG | 3 | 3 | 3 | 3 | 3 | 3 | 4 |  |  |  |  |  |  |  |  |  |  |  |
| 3_C1 | NEG | 3 | 3 | 4 | 3 | 4 | 3 | 3 |  |  |  |  |  |  |  |  |  |  |  |
| 3_D1 | NEG | 3 | 3 | 3 | 3 | 1 | 1 | 4 |  |  |  |  |  |  |  |  |  |  |  |
| 3_E1 | NEG | 4 | 4 | 3 | 3 | 4 | 4 |  |  |  |  |  |  |  |  |  |  |  |  |
| 3_F1 | NEG | 3 | 2 | 3 | 3 | 3 | 4 |  |  |  |  |  |  |  |  |  |  |  |  |
| 3_G1 | NEG | 3 | 3 | 4 | 4 | 2 | 2 |  |  |  |  |  |  |  |  |  |  |  |  |
| 3_H1 | NEG | 3 | 3 | 4 | 3 | 4 | 3 |  | 3.25 | 3.13 | 3.38 | 3.13 | 2.88 | 2.75 | 3.50 | 3.14 |  |  | 0 |
| 3_A12 | MEK | 2 | 3 | 2 | 2 | 3 | 2 | 3 |  |  |  |  |  |  |  |  |  |  |  |
| 3_B12 | MEK | 2 | 2 | 2 | 2 | 2 | 4 | 0 |  |  |  |  |  |  |  |  |  |  |  |
| 3_C12 | MEK | 3 | 3 | 2 | 2 | 2 | 1 | 2 |  |  |  |  |  |  |  |  |  |  |  |
| 3_D12 | MEK | 3 | 2 | 1 | 2 | 2 | 2 |  |  |  |  |  |  |  |  |  |  |  |  |
| 3_E12 | MEK | 3 | 3 | 1 | 2 | 3 | 2 |  | 0.80 | 0.83 | 0.47 | 0.64 | 0.83 | 0.80 | 0.48 | 0.69 |  |  | 3 |
| 3_F12 | TOX | 0 | 0 | 0 | 0 | 0 | 0 | 0 |  |  |  |  |  |  |  |  |  |  |  |
| 3_G12 | TOX | 0 | 0 | 0 | 0 | 0 | 0 |  |  |  |  |  |  |  |  |  |  |  |  |
| 3_H12 | TOX | 0 | 0 | 0 | 0 | 0 | 0 |  | 0.00 | 0.00 | 0.00 | 0.00 | 0.00 | 0.00 | 0.00 | 0.00 |  |  | 100 |
| 3_A2 | MAP3K2 | 3 | 3 | 4 | 3 | 4 | 4 |  | 0.92 | 0.92 | 1.23 | 0.92 | 1.39 | 1.45 |  | 1.14 | 0.25 | 0.88 | 0 |
| 3_B2 | DGKK | 4 | 4 | 4 | 4 | 3 | 3 |  | 1.23 | 1.28 | 1.19 | 1.28 | 1.04 | 1.09 |  | 1.19 | 0.10 | 1.01 | 0 |
| 3_C2 | CSNK1E | 1 | 0 | 2 | 2 | 3 | 4 |  | 0.31 | 0.00 | 0.59 | 0.64 | 1.04 | 1.45 |  | 0.67 | 0.52 | -0.55 | 3 |
| 3_D2 | JAK3 | 3 | 4 | 4 | 4 | 4 | 4 |  | 0.92 | 1.28 | 1.19 | 1.28 | 1.39 | 1.45 |  | 1.25 | 0.19 | 1.22 | 2 |
| 3_E2 | FLT3 | 2 | 1 | 2 | 2 | 2 | 1 |  | 0.62 | 0.32 | 0.59 | 0.64 | 0.70 | 0.36 |  | 0.54 | 0.16 | -0.96 | 1 |
| 3_F2 | RPS6KA3 | 3 | 4 | 5 | 4 | 2 | 3 |  | 0.92 | 1.28 | 1.48 | 1.28 | 0.70 | 1.09 |  | 1.13 | 0.28 | 0.83 | 1 |
| 3_G2 | MAP3K12 | 2 | 2 | 2 | 3 | 3 | 1 |  | 0.62 | 0.64 | 0.59 | 0.96 | 1.04 | 0.36 |  | 0.70 | 0.25 | -0.46 | 3 |
| 3_H2 | FN3KRP | 3 | 4 | 4 | 3 | 4 | 4 |  | 0.92 | 1.28 | 1.19 | 0.96 | 1.39 | 1.45 |  | 1.20 | 0.22 | 1.05 | 21 |
| 3_A3 | LATS2 | 4 | 4 | 2 | 1 | 4 | 4 |  | 1.23 | 1.28 | 0.59 | 0.32 | 1.39 | 1.45 |  | 1.04 | 0.47 | 0.59 | 5 |
| 3_B3 | MPP2 | 3 | 4 | 2 | 1 | 3 | 4 |  | 0.92 | 1.28 | 0.59 | 0.32 | 1.04 | 1.45 |  | 0.94 | 0.42 | 0.25 | 0 |
| 3_C3 | RELA | 2 | 1 | 1 | 2 | 3 | 2 |  | 0.62 | 0.32 | 0.30 | 0.64 | 1.04 | 0.73 |  | 0.61 | 0.28 | -0.75 | 7 |
| 3_D3 | STK22D | 4 | 4 | 4 | 3 | 4 | 4 |  | 1.23 | 1.28 | 1.19 | 0.96 | 1.39 | 1.45 |  | 1.25 | 0.17 | 1.21 | 2 |
| 3_E3 | PIP5K2A | 4 | 4 | 3 | 3 | 4 | 4 |  | 1.23 | 1.28 | 0.89 | 0.96 | 1.39 | 1.45 |  | 1.20 | 0.23 | 1.06 | 3 |
| 3_F3 | EFNA4 | 4 | 3 | 3 | 4 | 3 | 3 |  | 1.23 | 0.96 | 0.89 | 1.28 | 1.04 | 1.09 |  | 1.08 | 0.15 | 0.70 | 5 |
| 3_G3 | STK22C | 4 | 3 | 2 | 3 | 3 | 3 |  | 1.23 | 0.96 | 0.59 | 0.96 | 1.04 | 1.09 |  | 0.98 | 0.21 | 0.39 | 8 |
| 3_H3 | AKT2 | 2 | 2 | 1 | 2 | 2 | 2 |  | 0.62 | 0.64 | 0.30 | 0.64 | 0.70 | 0.73 |  | 0.60 | 0.16 | -0.76 | 6 |
| 3_A4 | P101-PI3K | 3 | 3 | 2 | 2 | 1 | 2 |  | 0.92 | 0.96 | 0.59 | 0.64 | 0.35 | 0.73 |  | 0.70 | 0.23 | -0.47 | 7 |
| 3_B4 | BUB1 | 4 | 4 | 6 | 5 | 4 | 4 |  | 1.23 | 1.28 | 1.78 | 1.60 | 1.39 | 1.45 |  | 1.46 | 0.21 | 1.83 | 8 |
| 3_C4 | CCRK | 0 | 0 | 2 | 2 | 3 | 1 | 0 | 0.00 | 0.00 | 0.59 | 0.64 | 1.04 | 0.36 | 0.00 | 0.38 | 0.41 | -1.44 | 3 |
| 3_D4 | FGFR1 | 2 | 1 | 2 | 2 | 3 | 4 |  | 0.62 | 0.32 | 0.59 | 0.64 | 1.04 | 1.45 |  | 0.78 | 0.40 | -0.23 | 1 |
| 3_E4 | DAPK1 | 3 | 3 | 3 | 3 | 2 | 2 |  | 0.92 | 0.96 | 0.89 | 0.96 | 0.70 | 0.73 |  | 0.86 | 0.12 | 0.02 | 0 |
| 3_F4 | ADCK1 | 3 | 2 | 1 | 1 | 1 | 2 |  | 0.92 | 0.64 | 0.30 | 0.32 | 0.35 | 0.73 |  | 0.54 | 0.26 | -0.94 | 5 |
| 3_G4 | PI4KII | 4 | 4 | 2 | 2 | 4 | 4 |  | 1.23 | 1.28 | 0.59 | 0.64 | 1.39 | 1.45 |  | 1.10 | 0.38 | 0.75 | 11 |
| 3_H4 | KIAA0999 | 3 | 3 | 2 | 2 | 3 | 4 |  | 0.92 | 0.96 | 0.59 | 0.64 | 1.04 | 1.45 |  | 0.94 | 0.31 | 0.25 | 1 |
| 3_A5 | DGKG | 3 | 4 | 3 | 4 | 4 | 4 |  | 0.92 | 1.28 | 0.89 | 1.28 | 1.39 | 1.45 |  | 1.20 | 0.24 | 1.07 | 0 |
| 3_B5 | UHMK1 | 4 | 3 | 2 | 2 | 4 | 4 |  | 1.23 | 0.96 | 0.59 | 0.64 | 1.39 | 1.45 |  | 1.04 | 0.37 | 0.59 | 5 |
| 3_C5 | MAPK9 | 3 | 3 | 5 | 2 | 2 | 4 |  | 0.92 | 0.96 | 1.48 | 0.64 | 0.70 | 1.45 |  | 1.03 | 0.36 | 0.53 | 0 |
| 3_D5 | PNKP | 0 | 0 | 3 | 2 | 3 | 0 | 0 | 0.00 | 0.00 | 0.89 | 0.64 | 1.04 | 0.00 | 0.00 | 0.37 | 0.47 | -1.47 | 5 |
| 3_E5 | FASTK | 4 | 4 | 3 | 3 | 4 | 4 |  | 1.23 | 1.28 | 0.89 | 0.96 | 1.39 | 1.45 |  | 1.20 | 0.23 | 1.06 | 6 |
| 3_F5 | MAP3K8 | 4 | 4 | 4 | 5 | 4 | 4 |  | 1.23 | 1.28 | 1.19 | 1.60 | 1.39 | 1.45 |  | 1.36 | 0.16 | 1.53 | 0 |
| 3_G5 | TAF1L | 2 | 1 | 3 | 3 | 3 | 2 |  | 0.62 | 0.32 | 0.89 | 0.96 | 1.04 | 0.73 |  | 0.76 | 0.27 | -0.28 | 2 |
| 3_H5 | PLK4 | 0 | 0 | 1 | 2 | 0 | 0 | 0 | 0.00 | 0.00 | 0.30 | 0.64 | 0.00 | 0.00 | 0.00 | 0.13 | 0.25 | -2.18 | 14 |
| 3_A6 | MAST3 | 3 | 4 | 2 | 2 | 4 | 4 |  | 0.92 | 1.28 | 0.59 | 0.64 | 1.39 | 1.45 |  | 1.05 | 0.38 | 0.59 | 12 |
| 3_B6 | PRKCM | 2 | 3 | 3 | 3 | 4 | 4 |  | 0.62 | 0.96 | 0.89 | 0.96 | 1.39 | 1.45 |  | 1.05 | 0.32 | 0.59 | 0 |
| 3_C6 | TNK2 | 1 | 0 | 2 | 3 | 3 | 3 |  | 0.31 | 0.00 | 0.59 | 0.96 | 1.04 | 1.09 |  | 0.67 | 0.44 | -0.57 | 0 |
| 3_D6 | ERBB2 | 1 | 1 | 1 | 1 | 3 | 4 |  | 0.31 | 0.32 | 0.30 | 0.32 | 1.04 | 1.45 |  | 0.62 | 0.50 | -0.70 | 0 |
| 3_E6 | PRKG2 | 3 | 4 | 2 | 2 | 4 | 4 |  | 0.92 | 1.28 | 0.59 | 0.64 | 1.39 | 1.45 |  | 1.05 | 0.38 | 0.59 | 0 |
| 3_F6 | CDK8 | 3 | 3 | 3 | 3 | 4 | 4 |  | 0.92 | 0.96 | 0.89 | 0.96 | 1.39 | 1.45 |  | 1.10 | 0.26 | 0.74 | 0 |
| 3_G6 | CAMK1G | 3 | 3 | 2 | 2 | 4 | 4 |  | 0.92 | 0.96 | 0.59 | 0.64 | 1.39 | 1.45 |  | 0.99 | 0.36 | 0.43 | 0 |
| 3_H6 | HIPK3 | 3 | 4 | 0 | 0 | 1 | 1 |  | 0.92 | 1.28 | 0.00 | 0.00 | 0.35 | 0.36 |  | 0.49 | 0.52 | -1.11 | 7 |
| 3_A7 | EFNA3 | 4 | 4 | 2 | 2 | 4 | 4 |  | 1.23 | 1.28 | 0.59 | 0.64 | 1.39 | 1.45 |  | 1.10 | 0.38 | 0.75 | 10 |
| 3_B7 | PIK4CB | 4 | 4 | 3 | 2 | 3 | 4 |  | 1.23 | 1.28 | 0.89 | 0.64 | 1.04 | 1.45 |  | 1.09 | 0.29 | 0.72 | 0 |
| 3_C7 | PDIK1L | 4 | 4 | 8 | 0 | 4 | 4 |  | 1.23 | 1.28 | 2.37 | 0.00 | 1.39 | 1.45 |  | 1.29 | 0.76 | 1.32 | 1 |
| 3_D7 | PRKAG1 | 3 | 3 | 2 | 2 | 4 | 4 |  | 0.92 | 0.96 | 0.59 | 0.64 | 1.39 | 1.45 |  | 0.99 | 0.36 | 0.43 | 5 |
| 3_E7 | MPP1 | 3 | 3 | 3 | 3 | 4 | 2 |  | 0.92 | 0.96 | 0.89 | 0.96 | 1.39 | 0.73 |  | 0.98 | 0.22 | 0.37 | 0 |
| 3_F7 | MKNK1 | 4 | 4 | 6 | 5 | 4 | 4 | 4 | 1.23 | 1.28 | 1.78 | 1.60 | 1.39 | 1.45 | 1.14 | 1.41 | 0.22 | 1.70 | 0 |
| 3_G7 | HSMDPKIN | 3 | 4 | 0 | 0 | 3 | 4 |  | 0.92 | 1.28 | 0.00 | 0.00 | 1.04 | 1.45 |  | 0.78 | 0.63 | -0.21 | 0 |
| 3_H7 | TYRO3 | 4 | 4 | 5 | 4 | 4 | 4 |  | 1.23 | 1.28 | 1.48 | 1.28 | 1.39 | 1.45 |  | 1.35 | 0.10 | 1.52 | 0 |
| 3_A8 | BCKDK | 4 | 4 | 4 | 4 | 4 | 4 |  | 1.23 | 1.28 | 1.19 | 1.28 | 1.39 | 1.45 |  | 1.30 | 0.10 | 1.37 | 3 |
| 3_B8 | DLG4 | 4 | 4 | 4 | 5 | 4 | 4 |  | 1.23 | 1.28 | 1.19 | 1.60 | 1.39 | 1.45 |  | 1.36 | 0.16 | 1.53 | 0 |
| 3_C8 | AXL | 3 | 4 | 2 | 2 | 4 | 3 |  | 0.92 | 1.28 | 0.59 | 0.64 | 1.39 | 1.09 |  | 0.99 | 0.33 | 0.41 | 3 |
| 3_D8 | FLJ10761 | 2 | 3 | 1 | 1 | 4 | 4 |  | 0.62 | 0.96 | 0.30 | 0.32 | 1.39 | 1.45 |  | 0.84 | 0.51 | -0.04 | 0 |
| 3_E8 | HSPB8 | 4 | 3 | 2 | 2 | 2 | 2 |  | 1.23 | 0.96 | 0.59 | 0.64 | 0.70 | 0.73 |  | 0.81 | 0.24 | -0.14 | 0 |
| 3_F8 | PIK3C2G | 3 | 4 | 0 | 0 | 0 | 2 |  | 0.92 | 1.28 | 0.00 | 0.00 | 0.00 | 0.73 |  | 0.49 | 0.56 | -1.11 | 0 |
| 3_G8 | STK10 | 1 | 4 | 3 | 3 | 3 | 3 |  | 0.31 | 1.28 | 0.89 | 0.96 | 1.04 | 1.09 |  | 0.93 | 0.33 | 0.23 | 1 |
| 3_H8 | MAPK3 | 3 | 4 | 3 | 3 | 4 | 3 |  | 0.92 | 1.28 | 0.89 | 0.96 | 1.39 | 1.09 |  | 1.09 | 0.21 | 0.72 | 8 |
| 3_A9 | ITPKA | 4 | 3 | 5 | 4 | 4 | 4 |  | 1.23 | 0.96 | 1.48 | 1.28 | 1.39 | 1.45 |  | 1.30 | 0.19 | 1.36 | 5 |
| 3_B9 | BTK | 3 | 4 | 3 | 3 | 4 | 4 |  | 0.92 | 1.28 | 0.89 | 0.96 | 1.39 | 1.45 |  | 1.15 | 0.25 | 0.90 | 0 |
| 3_C9 | ANKRD3 | 4 | 3 | 3 | 3 | 4 | 0 |  | 1.23 | 0.96 | 0.89 | 0.96 | 1.39 | 0.00 |  | 0.91 | 0.48 | 0.16 | 0 |
| 3_D9 | IGF1R | 3 | 2 | 3 | 2 | 4 | 4 |  | 0.92 | 0.64 | 0.89 | 0.64 | 1.39 | 1.45 |  | 0.99 | 0.36 | 0.42 | 1 |
| 3_E9 | CSF1R | 3 | 3 | 6 | 4 | 3 | 2 |  | 0.92 | 0.96 | 1.78 | 1.28 | 1.04 | 0.73 |  | 1.12 | 0.37 | 0.81 | 0 |
| 3_F9 | CDK4 | 2 | 2 | 3 | 3 | 4 | 4 |  | 0.62 | 0.64 | 0.89 | 0.96 | 1.39 | 1.45 |  | 0.99 | 0.36 | 0.42 | 3 |
| 3_G9 | AIP1 | 4 | 1 | 3 | 2 | 3 | 4 |  | 1.23 | 0.32 | 0.89 | 0.64 | 1.04 | 1.45 |  | 0.93 | 0.41 | 0.23 | 0 |
| 3_H9 | YES1 | 4 | 4 | 3 | 3 | 4 | 4 |  | 1.23 | 1.28 | 0.89 | 0.96 | 1.39 | 1.45 |  | 1.20 | 0.23 | 1.06 | 3 |
| 3_A10 | NEK6 | 4 | 4 | 4 | 3 | 4 | 4 |  | 1.23 | 1.28 | 1.19 | 0.96 | 1.39 | 1.45 |  | 1.25 | 0.17 | 1.21 | 2 |
| 3_B10 | MAP3K15 | 4 | 1 | 6 | 5 | 4 | 4 |  | 1.23 | 0.32 | 1.78 | 1.60 | 1.39 | 1.45 |  | 1.30 | 0.51 | 1.35 | 2 |
| 3_C10 | RIPK1 | 4 | 4 | 5 | 4 | 4 | 0 |  | 1.23 | 1.28 | 1.48 | 1.28 | 1.39 | 0.00 |  | 1.11 | 0.55 | 0.79 | 5 |
| 3_D10 | NEK9 | 4 | 3 | 3 | 3 | 4 | 4 |  | 1.23 | 0.96 | 0.89 | 0.96 | 1.39 | 1.45 |  | 1.15 | 0.24 | 0.90 | 7 |
| 3_E10 | DYRK2 | 4 | 4 | 2 | 3 | 3 | 3 |  | 1.23 | 1.28 | 0.59 | 0.96 | 1.04 | 1.09 |  | 1.03 | 0.25 | 0.55 | 5 |
| 3_F10 | PIM1 | 3 | 2 | 3 | 3 | 4 | 4 |  | 0.92 | 0.64 | 0.89 | 0.96 | 1.39 | 1.45 |  | 1.04 | 0.32 | 0.58 | 0 |
| 3_G10 | ABL1 | 4 | 4 | 3 | 3 | 4 | 4 |  | 1.23 | 1.28 | 0.89 | 0.96 | 1.39 | 1.45 |  | 1.20 | 0.23 | 1.06 | 6 |
| 3_H10 | NYD-SP25 | 4 | 4 | 3 | 3 | 4 | 4 |  | 1.23 | 1.28 | 0.89 | 0.96 | 1.39 | 1.45 |  | 1.20 | 0.23 | 1.06 | 9 |
| 3_A11 | BLK | 4 | 4 | 4 | 3 | 4 | 4 |  | 1.23 | 1.28 | 1.19 | 0.96 | 1.39 | 1.45 |  | 1.25 | 0.17 | 1.21 | 1 |
| 3_B11 | GSK3A | 3 | 2 | 4 | 4 | 3 | 4 |  | 0.92 | 0.64 | 1.19 | 1.28 | 1.04 | 1.45 |  | 1.09 | 0.29 | 0.72 | 5 |
| 3_C11 | CDC42BPB | 4 | 4 | 5 | 4 | 4 | 4 |  | 1.23 | 1.28 | 1.48 | 1.28 | 1.39 | 1.45 |  | 1.35 | 0.10 | 1.52 | 0 |
| 3_D11 | FLJ23356 | 4 | 4 | 3 | 3 | 4 | 3 |  | 1.23 | 1.28 | 0.89 | 0.96 | 1.39 | 1.09 |  | 1.14 | 0.19 | 0.88 | 1 |
| 3_E11 | PRKCL2 | 4 | 4 | 2 | 2 | 3 | 3 |  | 1.23 | 1.28 | 0.59 | 0.64 | 1.04 | 1.09 |  | 0.98 | 0.29 | 0.39 | 0 |
| 3_F11 | DGKQ | 4 | 4 | 3 | 4 | 4 | 4 |  | 1.23 | 1.28 | 0.89 | 1.28 | 1.39 | 1.45 |  | 1.25 | 0.20 | 1.22 | 0 |
| 3_G11 | PAK6 | 2 | 3 | 2 | 3 | 4 | 2 |  | 0.62 | 0.96 | 0.59 | 0.96 | 1.39 | 0.73 |  | 0.87 | 0.30 | 0.07 | 0 |
| 3_H11 | CDADC1 | 3 | 4 | 3 | 3 | 4 | 3 |  | 0.92 | 1.28 | 0.89 | 0.96 | 1.39 | 1.09 |  | 1.09 | 0.21 | 0.72 | 10 |
| 4_A1 | NEG | 4 | 3 | 4 | 4 |  |  |  |  |  |  |  |  |  |  |  |  |  |  |
| 4_B1 | NEG | 4 | 4 | 4 | 4 |  |  |  |  |  |  |  |  |  |  |  |  |  |  |
| 4_C1 | NEG | 4 | 4 | 4 | 5 |  |  |  |  |  |  |  |  |  |  |  |  |  |  |
| 4_D1 | NEG | 3 | 3 | 3 | 4 |  |  |  |  |  |  |  |  |  |  |  |  |  |  |
| 4_E1 | NEG | 3 | 3 | 4 | 5 |  |  |  |  |  |  |  |  |  |  |  |  |  |  |
| 4_F1 | NEG | 4 | 4 | 4 | 4 |  |  |  |  |  |  |  |  |  |  |  |  |  |  |
| 4_G1 | NEG | 4 | 4 | 4 | 4 |  |  |  |  |  |  |  |  |  |  |  |  |  |  |
| 4_H1 | NEG | 4 | 4 | 4 | 4 |  |  |  | 3.75 | 3.63 | 3.88 | 4.25 |  |  |  | 3.88 |  |  | 0 |
| 4_A12 | MEK | 2 | 3 | 2 | 2 |  |  |  |  |  |  |  |  |  |  |  |  |  |  |
| 4_B12 | MEK | 2 | 4 | 2 | 1 |  |  |  |  |  |  |  |  |  |  |  |  |  |  |
| 4_C12 | MEK | 3 | 3 | 2 | 0 |  |  |  |  |  |  |  |  |  |  |  |  |  |  |
| 4_D12 | MEK | 2 | 2 | 2 | 2 |  |  |  |  |  |  |  |  |  |  |  |  |  |  |
| 4_E12 | MEK | 2 | 2 | 3 | 2 |  |  |  | 0.59 | 0.77 | 0.57 | 0.33 |  |  |  | 0.56 |  |  | 12 |
| 4_F12 | TOX | 0 | 0 | 0 | 0 |  |  |  |  |  |  |  |  |  |  |  |  |  |  |
| 4_G12 | TOX | 0 | 0 | 0 | 0 |  |  |  |  |  |  |  |  |  |  |  |  |  |  |
| 4_H12 | TOX | 0 | 0 | 0 | 0 |  |  |  | 0.00 | 0.00 | 0.00 | 0.00 |  |  |  | 0.00 |  |  | 100 |
| 4_A2 | STK31 | 4 | 4 | 3 | 4 |  |  |  | 1.07 | 1.10 | 0.77 | 0.94 |  |  |  | 0.97 | 0.15 | 0.36 | 4 |
| 4_B2 | CDKN2C | 2 | 4 | 3 | 3 |  |  |  | 0.53 | 1.10 | 0.77 | 0.71 |  |  |  | 0.78 | 0.24 | -0.22 | 18 |
| 4_C2 | CAMK1 | 3 | 3 | 4 | 4 |  |  |  | 0.80 | 0.83 | 1.03 | 0.94 |  |  |  | 0.90 | 0.11 | 0.15 | 11 |
| 4_D2 | EPHB6 | 3 | 3 | 4 | 4 |  |  |  | 0.80 | 0.83 | 1.03 | 0.94 |  |  |  | 0.90 | 0.11 | 0.15 | 11 |
| 4_E2 | MAST4 | 4 | 4 | 5 | 5 |  |  |  | 1.07 | 1.10 | 1.29 | 1.18 |  |  |  | 1.16 | 0.10 | 0.93 | 11 |
| 4_F2 | TGFBR1 | 4 | 4 | 4 | 4 |  |  |  | 1.07 | 1.10 | 1.03 | 0.94 |  |  |  | 1.04 | 0.07 | 0.56 | 3 |
| 4_G2 | PAK1 | 4 | 3 | 3 | 4 |  |  |  | 1.07 | 0.83 | 0.77 | 0.94 |  |  |  | 0.90 | 0.13 | 0.15 | 0 |
| 4_H2 | ACVR1 | 3 | 3 | 4 | 3 |  |  |  | 0.80 | 0.83 | 1.03 | 0.71 |  |  |  | 0.84 | 0.14 | -0.03 | 22 |
| 4_A3 | AKT3 | 4 | 4 | 3 | 3 |  |  |  | 1.07 | 1.10 | 0.77 | 0.71 |  |  |  | 0.91 | 0.20 | 0.18 | 14 |
| 4_B3 | MAP2K3 | 2 | 4 | 2 | 2 |  |  |  | 0.53 | 1.10 | 0.52 | 0.47 |  |  |  | 0.66 | 0.30 | -0.60 | 8 |
| 4_C3 | MARK3 | 3 | 3 | 4 | 3 |  |  |  | 0.80 | 0.83 | 1.03 | 0.71 |  |  |  | 0.84 | 0.14 | -0.03 | 11 |
| 4_D3 | MARK1 | 4 | 3 | 1 | 2 |  |  |  | 1.07 | 0.83 | 0.26 | 0.47 |  |  |  | 0.66 | 0.36 | -0.60 | 15 |
| 4_E3 | CKMT2 | 3 | 2 | 2 | 3 |  |  |  | 0.80 | 0.55 | 0.52 | 0.71 |  |  |  | 0.64 | 0.13 | -0.64 | 0 |
| 4_F3 | CDK5R2 | 4 | 3 | 3 | 2 |  |  |  | 1.07 | 0.83 | 0.77 | 0.47 |  |  |  | 0.78 | 0.25 | -0.21 | 24 |
| 4_G3 | RYK | 4 | 4 | 5 | 6 |  |  |  | 1.07 | 1.10 | 1.29 | 1.41 |  |  |  | 1.22 | 0.16 | 1.11 | 12 |
| 4_H3 | PRKACB | 4 | 4 | 4 | 4 |  |  |  | 1.07 | 1.10 | 1.03 | 0.94 |  |  |  | 1.04 | 0.07 | 0.56 | 5 |
| 4_A4 | NLK | 4 | 4 | 4 | 4 |  |  |  | 1.07 | 1.10 | 1.03 | 0.94 |  |  |  | 1.04 | 0.07 | 0.56 | 0 |
| 4_B4 | LCK | 3 | 2 | 3 | 2 |  |  |  | 0.80 | 0.55 | 0.77 | 0.47 |  |  |  | 0.65 | 0.16 | -0.62 | 13 |
| 4_C4 | MGC42105 | 3 | 4 | 4 | 3 |  |  |  | 0.80 | 1.10 | 1.03 | 0.71 |  |  |  | 0.91 | 0.19 | 0.18 | 3 |
| 4_D4 | PRKAR2A | 2 | 3 | 3 | 4 |  |  |  | 0.53 | 0.83 | 0.77 | 0.94 |  |  |  | 0.77 | 0.17 | -0.25 | 20 |
| 4_E4 | PIK3R3 | 4 | 4 | 5 | 4 |  |  |  | 1.07 | 1.10 | 1.29 | 0.94 |  |  |  | 1.10 | 0.14 | 0.75 | 8 |
| 4_F4 | LOC91461 | 4 | 4 | 5 | 3 |  |  |  | 1.07 | 1.10 | 1.29 | 0.71 |  |  |  | 1.04 | 0.24 | 0.58 | 26 |
| 4_G4 | PDXK | 4 | 4 | 2 | 2 |  |  |  | 1.07 | 1.10 | 0.52 | 0.47 |  |  |  | 0.79 | 0.34 | -0.19 | 21 |
| 4_H4 | STK39 | 4 | 4 | 4 | 3 |  |  |  | 1.07 | 1.10 | 1.03 | 0.71 |  |  |  | 0.98 | 0.18 | 0.38 | 12 |
| 4_A5 | PFKFB3 | 3 | 4 | 4 | 4 |  |  |  | 0.80 | 1.10 | 1.03 | 0.94 |  |  |  | 0.97 | 0.13 | 0.36 | 0 |
| 4_B5 | CIB2 | 3 | 4 | 3 | 4 |  |  |  | 0.80 | 1.10 | 0.77 | 0.94 |  |  |  | 0.90 | 0.15 | 0.16 | 9 |
| 4_C5 | CHEK1 | 4 | 4 | 0 | 1 |  |  |  | 1.07 | 1.10 | 0.00 | 0.24 |  |  |  | 0.60 | 0.57 | -0.76 | 22 |
| 4_D5 | PINK1 | 3 | 3 | 3 | 3 |  |  |  | 0.80 | 0.83 | 0.77 | 0.71 |  |  |  | 0.78 | 0.05 | -0.23 | 4 |
| 4_E5 | ACVR2 | 4 | 4 | 3 | 3 |  |  |  | 1.07 | 1.10 | 0.77 | 0.71 |  |  |  | 0.91 | 0.20 | 0.18 | 10 |
| 4_F5 | DKFZP761P0423 | 4 | 4 | 3 | 2 |  |  |  | 1.07 | 1.10 | 0.77 | 0.47 |  |  |  | 0.85 | 0.29 | 0.00 | 23 |
| 4_G5 | NEK8 | 1 | 1 | 0 | 0 |  |  |  | 0.27 | 0.28 | 0.00 | 0.00 |  |  |  | 0.14 | 0.16 | -2.18 | 16 |
| 4_H5 | MATK | 3 | 3 | 5 | 5 |  |  |  | 0.80 | 0.83 | 1.29 | 1.18 |  |  |  | 1.02 | 0.25 | 0.52 | 44 |
| 4_A6 | TESK2 | 4 | 4 | 4 | 4 |  |  |  | 1.07 | 1.10 | 1.03 | 0.94 |  |  |  | 1.04 | 0.07 | 0.56 | 14 |
| 4_B6 | AAK1 | 4 | 4 | 4 | 4 |  |  |  | 1.07 | 1.10 | 1.03 | 0.94 |  |  |  | 1.04 | 0.07 | 0.56 | 1 |
| 4_C6 | SCAP1 | 4 | 4 | 2 | 3 |  |  |  | 1.07 | 1.10 | 0.52 | 0.71 |  |  |  | 0.85 | 0.28 | -0.01 | 0 |
| 4_D6 | MUSK | 4 | 3 | 2 | 3 |  |  |  | 1.07 | 0.83 | 0.52 | 0.71 |  |  |  | 0.78 | 0.23 | -0.22 | 5 |
| 4_E6 | PRKWNK3 | 4 | 4 | 4 | 4 |  |  |  | 1.07 | 1.10 | 1.03 | 0.94 |  |  |  | 1.04 | 0.07 | 0.56 | 8 |
| 4_F6 | DUSTYPK | 4 | 4 | 5 | 2 |  |  |  | 1.07 | 1.10 | 1.29 | 0.47 |  |  |  | 0.98 | 0.36 | 0.40 | 24 |
| 4_G6 | GK | 2 | 1 | 4 | 2 |  |  |  | 0.53 | 0.28 | 1.03 | 0.47 |  |  |  | 0.58 | 0.32 | -0.83 | 22 |
| 4_H6 | RPS6KB1 | 3 | 2 | 2 | 3 |  |  |  | 0.80 | 0.55 | 0.52 | 0.71 |  |  |  | 0.64 | 0.13 | -0.64 | 21 |
| 4_A7 | EPHA7 | 4 | 3 | 3 | 4 |  |  |  | 1.07 | 0.83 | 0.77 | 0.94 |  |  |  | 0.90 | 0.13 | 0.15 | 12 |
| 4_B7 | PIK3R2 | 2 | 3 | 2 | 1 |  |  |  | 0.53 | 0.83 | 0.52 | 0.24 |  |  |  | 0.53 | 0.24 | -0.99 | 22 |
| 4_C7 | CDC2L2 | 3 | 4 | 5 | 3 |  |  |  | 0.80 | 1.10 | 1.29 | 0.71 |  |  |  | 0.97 | 0.27 | 0.37 | 9 |
| 4_D7 | ULK2 | 3 | 3 | 5 | 4 |  |  |  | 0.80 | 0.83 | 1.29 | 0.94 |  |  |  | 0.96 | 0.23 | 0.34 | 14 |
| 4_E7 | PRKCZ | 3 | 2 | 3 | 3 |  |  |  | 0.80 | 0.55 | 0.77 | 0.71 |  |  |  | 0.71 | 0.11 | -0.44 | 18 |
| 4_F7 | FLJ23356 | 4 | 4 | 5 | 5 |  |  |  | 1.07 | 1.10 | 1.29 | 1.18 |  |  |  | 1.16 | 0.10 | 0.93 | 13 |
| 4_G7 | KSR2 | 4 | 4 | 4 | 3 |  |  |  | 1.07 | 1.10 | 1.03 | 0.71 |  |  |  | 0.98 | 0.18 | 0.38 | 21 |
| 4_H7 | RFK | 4 | 4 | 5 | 5 |  |  |  | 1.07 | 1.10 | 1.29 | 1.18 |  |  |  | 1.16 | 0.10 | 0.93 | 32 |
| 4_A8 | STK35 | 2 | 3 | 4 | 4 |  |  |  | 0.53 | 0.83 | 1.03 | 0.94 |  |  |  | 0.83 | 0.22 | -0.06 | 11 |
| 4_B8 | IRAK2 | 3 | 3 | 5 | 5 |  |  |  | 0.80 | 0.83 | 1.29 | 1.18 |  |  |  | 1.02 | 0.25 | 0.52 | 1 |
| 4_C8 | STK3 | 3 | 4 | 5 | 4 |  |  |  | 0.80 | 1.10 | 1.29 | 0.94 |  |  |  | 1.03 | 0.21 | 0.55 | 10 |
| 4_D8 | MGC8407 | 4 | 3 | 0 | 0 |  |  |  | 1.07 | 0.83 | 0.00 | 0.00 |  |  |  | 0.47 | 0.56 | -1.15 | 4 |
| 4_E8 | ITK | 4 | 2 | 4 | 4 |  |  |  | 1.07 | 0.55 | 1.03 | 0.94 |  |  |  | 0.90 | 0.24 | 0.14 | 10 |
| 4_F8 | PDK3 | 3 | 4 | 2 | 3 |  |  |  | 0.80 | 1.10 | 0.52 | 0.71 |  |  |  | 0.78 | 0.25 | -0.22 | 2 |
| 4_G8 | PRKAR1A | 3 | 4 | 3 | 5 |  |  |  | 0.80 | 1.10 | 0.77 | 1.18 |  |  |  | 0.96 | 0.21 | 0.34 | 9 |
| 4_H8 | TTBK2 | 4 | 4 | 5 | 5 |  |  |  | 1.07 | 1.10 | 1.29 | 1.18 |  |  |  | 1.16 | 0.10 | 0.93 | 38 |
| 4_A9 | STK11 | 4 | 2 | 3 | 3 |  |  |  | 1.07 | 0.55 | 0.77 | 0.71 |  |  |  | 0.77 | 0.22 | -0.24 | 26 |
| 4_B9 | ACVR1C | 4 | 4 | 4 | 5 |  |  |  | 1.07 | 1.10 | 1.03 | 1.18 |  |  |  | 1.09 | 0.06 | 0.74 | 18 |
| 4_C9 | FLT4 | 4 | 4 | 4 | 4 |  |  |  | 1.07 | 1.10 | 1.03 | 0.94 |  |  |  | 1.04 | 0.07 | 0.56 | 11 |
| 4_D9 | AURKA | 2 | 4 | 3 | 2 |  |  |  | 0.53 | 1.10 | 0.77 | 0.47 |  |  |  | 0.72 | 0.29 | -0.40 | 10 |
| 4_E9 | CAMKK1 | 4 | 4 | 2 | 4 |  |  |  | 1.07 | 1.10 | 0.52 | 0.94 |  |  |  | 0.91 | 0.27 | 0.17 | 7 |
| 4_F9 | DGKA | 4 | 3 | 2 | 3 |  |  |  | 1.07 | 0.83 | 0.52 | 0.71 |  |  |  | 0.78 | 0.23 | -0.22 | 8 |
| 4_G9 | NUP62 | 3 | 4 | 4 | 4 |  |  |  | 0.80 | 1.10 | 1.03 | 0.94 |  |  |  | 0.97 | 0.13 | 0.36 | 21 |
| 4_H9 | CDC42BPA | 2 | 4 | 2 | 2 |  |  |  | 0.53 | 1.10 | 0.52 | 0.47 |  |  |  | 0.66 | 0.30 | -0.60 | 40 |
| 4_A10 | JAK2 | 4 | 4 | 4 | 5 |  |  |  | 1.07 | 1.10 | 1.03 | 1.18 |  |  |  | 1.09 | 0.06 | 0.74 | 8 |
| 4_B10 | ACVR1B | 3 | 4 | 4 | 5 |  |  |  | 0.80 | 1.10 | 1.03 | 1.18 |  |  |  | 1.03 | 0.16 | 0.53 | 3 |
| 4_C10 | CDC2L1 | 4 | 4 | 3 | 3 |  |  |  | 1.07 | 1.10 | 0.77 | 0.71 |  |  |  | 0.91 | 0.20 | 0.18 | 13 |
| 4_D10 | PAK4 | 3 | 3 | 3 | 4 |  |  |  | 0.80 | 0.83 | 0.77 | 0.94 |  |  |  | 0.84 | 0.07 | -0.05 | 21 |
| 4_E10 | STK36 | 2 | 2 | 5 | 4 |  |  |  | 0.53 | 0.55 | 1.29 | 0.94 |  |  |  | 0.83 | 0.36 | -0.07 | 5 |
| 4_F10 | C9ORF12 | 0 | 4 | 3 | 4 |  |  |  | 0.00 | 1.10 | 0.77 | 0.94 |  |  |  | 0.70 | 0.49 | -0.45 | 18 |
| 4_G10 | ROR2 | 4 | 4 | 4 | 4 |  |  |  | 1.07 | 1.10 | 1.03 | 0.94 |  |  |  | 1.04 | 0.07 | 0.56 | 13 |
| 4_H10 | GSG2 | 2 | 3 | 3 | 2 |  |  |  | 0.53 | 0.83 | 0.77 | 0.47 |  |  |  | 0.65 | 0.18 | -0.61 | 11 |
| 4_A11 | BRD2 | 4 | 4 | 3 | 3 |  |  |  | 1.07 | 1.10 | 0.77 | 0.71 |  |  |  | 0.91 | 0.20 | 0.18 | 22 |
| 4_B11 | CPNE3 | 3 | 3 | 4 | 4 |  |  |  | 0.80 | 0.83 | 1.03 | 0.94 |  |  |  | 0.90 | 0.11 | 0.15 | 9 |
| 4_C11 | MAP2K7 | 3 | 3 | 0 | 1 |  |  |  | 0.80 | 0.83 | 0.00 | 0.24 |  |  |  | 0.47 | 0.41 | -1.18 | 3 |
| 4_D11 | MAPK1 | 4 | 4 | 3 | 3 |  |  |  | 1.07 | 1.10 | 0.77 | 0.71 |  |  |  | 0.91 | 0.20 | 0.18 | 4 |
| 4_E11 | MAPK8 | 4 | 4 | 3 | 2 |  |  |  | 1.07 | 1.10 | 0.77 | 0.47 |  |  |  | 0.85 | 0.29 | 0.00 | 6 |
| 4_F11 | PTK6 | 0 | 0 | 2 | 2 |  |  |  | 0.00 | 0.00 | 0.52 | 0.47 |  |  |  | 0.25 | 0.29 | -1.84 | 12 |
| 4_G11 | PIP5K2C | 4 | 3 | 5 | 6 |  |  |  | 1.07 | 0.83 | 1.29 | 1.41 |  |  |  | 1.15 | 0.26 | 0.90 | 14 |
| 4_H11 | HIPK1 | 4 | 4 | 3 | 3 |  |  |  | 1.07 | 1.10 | 0.77 | 0.71 |  |  |  | 0.91 | 0.20 | 0.18 | 20 |
| 5_A1 | NEG | 3 | 3 | 2 | 1 |  |  |  |  |  |  |  |  |  |  |  |  |  |  |
| 5_B1 | NEG | 2 | 2 | 1 | 1 |  |  |  |  |  |  |  |  |  |  |  |  |  |  |
| 5_C1 | NEG | 2 | 3 | 1 | 1 |  |  |  |  |  |  |  |  |  |  |  |  |  |  |
| 5_D1 | NEG | 4 | 3 | 2 | 3 |  |  |  |  |  |  |  |  |  |  |  |  |  |  |
| 5_E1 | NEG | 3 | 2 | 2 | 2 |  |  |  |  |  |  |  |  |  |  |  |  |  |  |
| 5_F1 | NEG | 3 | 2 | 2 | 3 |  |  |  |  |  |  |  |  |  |  |  |  |  |  |
| 5_G1 | NEG | 1 | 1 | 2 | 2 |  |  |  |  |  |  |  |  |  |  |  |  |  |  |
| 5_H1 | NEG | 3 | 3 | 1 | 1 |  |  |  | 2.63 | 2.38 | 1.63 | 1.75 |  |  |  | 2.09 |  |  | 0 |
| 5_A12 | MEK | 2 | 3 | 0 | 1 |  |  |  |  |  |  |  |  |  |  |  |  |  |  |
| 5_B12 | MEK | 1 | 1 | 1 | 1 |  |  |  |  |  |  |  |  |  |  |  |  |  |  |
| 5_C12 | MEK | 1 | 1 | 1 | 1 |  |  |  |  |  |  |  |  |  |  |  |  |  |  |
| 5_D12 | MEK | 2 | 3 | 0 | 2 |  |  |  |  |  |  |  |  |  |  |  |  |  |  |
| 5_E12 | MEK | 0 | 1 | 1 | 0 |  |  |  | 0.46 | 0.76 | 0.37 | 0.57 |  |  |  | 0.54 |  |  | 0 |
| 5_F12 | TOX | 0 | 0 | 0 | 0 |  |  |  |  |  |  |  |  |  |  |  |  |  |  |
| 5_G12 | TOX | 0 | 0 | 0 | 0 |  |  |  |  |  |  |  |  |  |  |  |  |  |  |
| 5_H12 | TOX | 0 | 0 | 0 | 0 |  |  |  | 0.00 | 0.00 | 0.00 | 0.00 |  |  |  | 0.00 |  |  | 100 |
| 5_A2 | MAP3K11 | 1 | 1 | 0 | 0 |  |  |  | 0.38 | 0.42 | 0.00 | 0.00 |  |  |  | 0.20 | 0.23 | -1.98 | 18 |
| 5_B2 | CDK5 | 2 | 2 | 0 | 0 |  |  |  | 0.76 | 0.84 | 0.00 | 0.00 |  |  |  | 0.40 | 0.46 | -1.37 | 19 |
| 5_C2 | ITPK1 | 2 | 3 | 0 | 0 |  |  |  | 0.76 | 1.26 | 0.00 | 0.00 |  |  |  | 0.51 | 0.62 | -1.05 | 23 |
| 5_D2 | BCR | 3 | 2 | 1 | 1 |  |  |  | 1.14 | 0.84 | 0.62 | 0.57 |  |  |  | 0.79 | 0.26 | -0.18 | 28 |
| 5_E2 | ADCK2 | 1 | 2 | 2 | 2 |  |  |  | 0.38 | 0.84 | 1.23 | 1.14 |  |  |  | 0.90 | 0.38 | 0.14 | 27 |
| 5_F2 | SPHK1 | 2 | 2 | 2 | 2 |  |  |  | 0.76 | 0.84 | 1.23 | 1.14 |  |  |  | 0.99 | 0.23 | 0.43 | 36 |
| 5_G2 | PRKY | 2 | 3 | 2 | 2 |  |  |  | 0.76 | 1.26 | 1.23 | 1.14 |  |  |  | 1.10 | 0.23 | 0.75 | 34 |
| 5_H2 | MERTK | 0 | 0 | 0 | 0 |  |  |  | 0.00 | 0.00 | 0.00 | 0.00 |  |  |  | 0.00 | 0.00 | -2.59 | 28 |
| 5_A3 | AURKB | 2 | 1 | 0 | 0 |  |  |  | 0.76 | 0.42 | 0.00 | 0.00 |  |  |  | 0.30 | 0.37 | -1.69 | 10 |
| 5_B3 | ADCK4 | 1 | 1 | 0 | 0 |  |  |  | 0.38 | 0.42 | 0.00 | 0.00 |  |  |  | 0.20 | 0.23 | -1.98 | 0 |
| 5_C3 | ILK-2 | 2 | 2 | 1 | 2 |  |  |  | 0.76 | 0.84 | 0.62 | 1.14 |  |  |  | 0.84 | 0.22 | -0.04 | 37 |
| 5_D3 | DCK | 3 | 2 | 3 | 3 |  |  |  | 1.14 | 0.84 | 1.85 | 1.71 |  |  |  | 1.39 | 0.47 | 1.62 | 16 |
| 5_E3 | PI4K2B | 3 | 3 | 1 | 1 |  |  |  | 1.14 | 1.26 | 0.62 | 0.57 |  |  |  | 0.90 | 0.36 | 0.14 | 41 |
| 5_F3 | ASK | 4 | 3 | 2 | 2 |  |  |  | 1.52 | 1.26 | 1.23 | 1.14 |  |  |  | 1.29 | 0.16 | 1.33 | 17 |
| 5_G3 | MAP3K1 | 4 | 4 | 3 | 3 |  |  |  | 1.52 | 1.68 | 1.85 | 1.71 |  |  |  | 1.69 | 0.13 | 2.55 | 44 |
| 5_H3 | CERK | 3 | 4 | 2 | 2 |  |  |  | 1.14 | 1.68 | 1.23 | 1.14 |  |  |  | 1.30 | 0.26 | 1.36 | 21 |
| 5_A4 | LIMK2 | 3 | 2 | 1 | 1 |  |  |  | 1.14 | 0.84 | 0.62 | 0.57 |  |  |  | 0.79 | 0.26 | -0.18 | 14 |
| 5_B4 | HRI | 2 | 2 | 1 | 1 |  |  |  | 0.76 | 0.84 | 0.62 | 0.57 |  |  |  | 0.70 | 0.13 | -0.47 | 14 |
| 5_C4 | EPHA2 | 3 | 2 | 2 | 3 |  |  |  | 1.14 | 0.84 | 1.23 | 1.71 |  |  |  | 1.23 | 0.36 | 1.16 | 29 |
| 5_D4 | MGC16169 | 1 | 1 | 1 | 0 |  |  |  | 0.38 | 0.42 | 0.62 | 0.00 |  |  |  | 0.35 | 0.26 | -1.51 | 17 |
| 5_E4 | TRIB3 | 1 | 2 | 2 | 3 |  |  |  | 0.38 | 0.84 | 1.23 | 1.71 |  |  |  | 1.04 | 0.57 | 0.58 | 0 |
| 5_F4 | PDGFRB | 3 | 2 | 2 | 2 |  |  |  | 1.14 | 0.84 | 1.23 | 1.14 |  |  |  | 1.09 | 0.17 | 0.72 | 15 |
| 5_G4 | ZAP70 | 2 | 2 | 2 | 1 |  |  |  | 0.76 | 0.84 | 1.23 | 0.57 |  |  |  | 0.85 | 0.28 | 0.00 | 3 |
| 5_H4 | NUCKS | 3 | 4 | 2 | 2 |  |  |  | 1.14 | 1.68 | 1.23 | 1.14 |  |  |  | 1.30 | 0.26 | 1.36 | 23 |
| 5_A5 | GRK4 | 2 | 2 | 2 | 3 |  |  |  | 0.76 | 0.84 | 1.23 | 1.71 |  |  |  | 1.14 | 0.44 | 0.87 | 0 |
| 5_B5 | MAPKAPK3 | 3 | 2 | 0 | 0 |  |  |  | 1.14 | 0.84 | 0.00 | 0.00 |  |  |  | 0.50 | 0.59 | -1.08 | 3 |
| 5_C5 | PHKG1 | 2 | 2 | 2 | 2 |  |  |  | 0.76 | 0.84 | 1.23 | 1.14 |  |  |  | 0.99 | 0.23 | 0.43 | 8 |
| 5_D5 | STK22D | 3 | 3 | 2 | 3 |  |  |  | 1.14 | 1.26 | 1.23 | 1.71 |  |  |  | 1.34 | 0.26 | 1.48 | 0 |
| 5_E5 | FLJ23074 | 2 | 2 | 0 | 0 |  |  |  | 0.76 | 0.84 | 0.00 | 0.00 |  |  |  | 0.40 | 0.46 | -1.37 | 0 |
| 5_F5 | FGR | 4 | 4 | 1 | 1 |  |  |  | 1.52 | 1.68 | 0.62 | 0.57 |  |  |  | 1.10 | 0.59 | 0.75 | 9 |
| 5_G5 | STK23 | 4 | 2 | 3 | 3 |  |  |  | 1.52 | 0.84 | 1.85 | 1.71 |  |  |  | 1.48 | 0.45 | 1.91 | 5 |
| 5_H5 | CIT | 3 | 2 | 0 | 0 |  |  |  | 1.14 | 0.84 | 0.00 | 0.00 |  |  |  | 0.50 | 0.59 | -1.08 | 28 |
| 5_A6 | LOC340156 | 1 | 2 | 2 | 2 |  |  |  | 0.38 | 0.84 | 1.23 | 1.14 |  |  |  | 0.90 | 0.38 | 0.14 | 15 |
| 5_B6 | ACVR2B | 3 | 3 | 3 | 2 |  |  |  | 1.14 | 1.26 | 1.85 | 1.14 |  |  |  | 1.35 | 0.34 | 1.51 | 26 |
| 5_C6 | FUK | 4 | 4 | 1 | 1 |  |  |  | 1.52 | 1.68 | 0.62 | 0.57 |  |  |  | 1.10 | 0.59 | 0.75 | 24 |
| 5_D6 | SRP72 | 3 | 3 | 2 | 3 |  |  |  | 1.14 | 1.26 | 1.23 | 1.71 |  |  |  | 1.34 | 0.26 | 1.48 | 47 |
| 5_E6 | HIPK2 | 2 | 1 | 2 | 2 |  |  |  | 0.76 | 0.42 | 1.23 | 1.14 |  |  |  | 0.89 | 0.37 | 0.11 | 42 |
| 5_F6 | ROR1 | 4 | 4 | 3 | 2 |  |  |  | 1.52 | 1.68 | 1.85 | 1.14 |  |  |  | 1.55 | 0.30 | 2.12 | 32 |
| 5_G6 | PTK7 | 2 | 4 | 2 | 3 |  |  |  | 0.76 | 1.68 | 1.23 | 1.71 |  |  |  | 1.35 | 0.45 | 1.51 | 1 |
| 5_H6 | CDKL4 | 3 | 3 | 3 | 3 |  |  |  | 1.14 | 1.26 | 1.85 | 1.71 |  |  |  | 1.49 | 0.34 | 1.94 | 37 |
| 5_A7 | PRKX | 2 | 1 | 3 | 2 |  |  |  | 0.76 | 0.42 | 1.85 | 1.14 |  |  |  | 1.04 | 0.61 | 0.58 | 0 |
| 5_B7 | EGFR | 1 | 1 | 0 | 0 |  |  |  | 0.38 | 0.42 | 0.00 | 0.00 |  |  |  | 0.20 | 0.23 | -1.98 | 25 |
| 5_C7 | AK3 | 4 | 3 | 1 | 1 |  |  |  | 1.52 | 1.26 | 0.62 | 0.57 |  |  |  | 0.99 | 0.47 | 0.43 | 21 |
| 5_D7 | DGKI | 2 | 2 | 2 | 1 |  |  |  | 0.76 | 0.84 | 1.23 | 0.57 |  |  |  | 0.85 | 0.28 | 0.00 | 11 |
| 5_E7 | PRPS1 | 2 | 2 | 2 | 2 |  |  |  | 0.76 | 0.84 | 1.23 | 1.14 |  |  |  | 0.99 | 0.23 | 0.43 | 16 |
| 5_F7 | CRK7 | 2 | 2 | 1 | 2 |  |  |  | 0.76 | 0.84 | 0.62 | 1.14 |  |  |  | 0.84 | 0.22 | -0.04 | 28 |
| 5_G7 | RPS6KL1 | 4 | 3 | 2 | 2 |  |  |  | 1.52 | 1.26 | 1.23 | 1.14 |  |  |  | 1.29 | 0.16 | 1.33 | 16 |
| 5_H7 | KIAA2002 | 2 | 1 | 1 | 1 |  |  |  | 0.76 | 0.42 | 0.62 | 0.57 |  |  |  | 0.59 | 0.14 | -0.79 | 29 |
| 5_A8 | GNE | 3 | 1 | 0 | 0 |  |  |  | 1.14 | 0.42 | 0.00 | 0.00 |  |  |  | 0.39 | 0.54 | -1.40 | 13 |
| 5_B8 | ROS1 | 2 | 1 | 1 | 1 |  |  |  | 0.76 | 0.42 | 0.62 | 0.57 |  |  |  | 0.59 | 0.14 | -0.79 | 11 |
| 5_C8 | CSNK2A2 | 1 | 3 | 0 | 0 |  |  |  | 0.38 | 1.26 | 0.00 | 0.00 |  |  |  | 0.41 | 0.60 | -1.34 | 18 |
| 5_D8 | CSK | 4 | 2 | 1 | 1 |  |  |  | 1.52 | 0.84 | 0.62 | 0.57 |  |  |  | 0.89 | 0.44 | 0.11 | 20 |
| 5_E8 | CSNK1D | 2 | 2 | 3 | 2 |  |  |  | 0.76 | 0.84 | 1.85 | 1.14 |  |  |  | 1.15 | 0.49 | 0.90 | 0 |
| 5_F8 | CDKN1B | 3 | 2 | 2 | 2 |  |  |  | 1.14 | 0.84 | 1.23 | 1.14 |  |  |  | 1.09 | 0.17 | 0.72 | 17 |
| 5_G8 | RPS6KA6 | 2 | 3 | 3 | 2 |  |  |  | 0.76 | 1.26 | 1.85 | 1.14 |  |  |  | 1.25 | 0.45 | 1.22 | 20 |
| 5_H8 | OSR1 | 2 | 1 | 2 | 1 |  |  |  | 0.76 | 0.42 | 1.23 | 0.57 |  |  |  | 0.75 | 0.35 | -0.32 | 20 |
| 5_A9 | PRKAR1B | 0 | 3 | 0 | 1 |  |  |  | 0.00 | 1.26 | 0.00 | 0.57 |  |  |  | 0.46 | 0.60 | -1.20 | 0 |
| 5_B9 | HK3 | 1 | 3 | 1 | 2 |  |  |  | 0.38 | 1.26 | 0.62 | 1.14 |  |  |  | 0.85 | 0.42 | -0.01 | 21 |
| 5_C9 | VRK2 | 4 | 2 | 2 | 3 |  |  |  | 1.52 | 0.84 | 1.23 | 1.71 |  |  |  | 1.33 | 0.38 | 1.45 | 0 |
| 5_D9 | IKBKAP | 3 | 2 | 2 | 2 |  |  |  | 1.14 | 0.84 | 1.23 | 1.14 |  |  |  | 1.09 | 0.17 | 0.72 | 8 |
| 5_E9 | DYRK3 | 4 | 3 | 3 | 3 |  |  |  | 1.52 | 1.26 | 1.85 | 1.71 |  |  |  | 1.59 | 0.25 | 2.23 | 5 |
| 5_F9 | PACSIN1 | 1 | 3 | 2 | 1 |  |  |  | 0.38 | 1.26 | 1.23 | 0.57 |  |  |  | 0.86 | 0.45 | 0.03 | 18 |
| 5_G9 | SRPK1 | 1 | 3 | 0 | 0 |  |  |  | 0.38 | 1.26 | 0.00 | 0.00 |  |  |  | 0.41 | 0.60 | -1.34 | 20 |
| 5_H9 | RAGE | 3 | 2 | 2 | 2 |  |  |  | 1.14 | 0.84 | 1.23 | 1.14 |  |  |  | 1.09 | 0.17 | 0.72 | 22 |
| 5_A10 | MVK | 1 | 0 | 1 | 1 |  |  |  | 0.38 | 0.00 | 0.62 | 0.57 |  |  |  | 0.39 | 0.28 | -1.40 | 0 |
| 5_B10 | DDR2 | 2 | 1 | 2 | 3 |  |  |  | 0.76 | 0.42 | 1.23 | 1.71 |  |  |  | 1.03 | 0.56 | 0.55 | 3 |
| 5_C10 | KALRN | 1 | 4 | 2 | 4 |  |  |  | 0.38 | 1.68 | 1.23 | 2.29 |  |  |  | 1.40 | 0.80 | 1.65 | 0 |
| 5_D10 | ADRBK2 | 2 | 4 | 1 | 1 |  |  |  | 0.76 | 1.68 | 0.62 | 0.57 |  |  |  | 0.91 | 0.52 | 0.17 | 10 |
| 5_E10 | TYK2 | 2 | 4 | 0 | 0 |  |  |  | 0.76 | 1.68 | 0.00 | 0.00 |  |  |  | 0.61 | 0.80 | -0.73 | 3 |
| 5_F10 | DAPK3 | 3 | 2 | 3 | 3 |  |  |  | 1.14 | 0.84 | 1.85 | 1.71 |  |  |  | 1.39 | 0.47 | 1.62 | 5 |
| 5_G10 | PRKACA | 4 | 2 | 3 | 3 |  |  |  | 1.52 | 0.84 | 1.85 | 1.71 |  |  |  | 1.48 | 0.45 | 1.91 | 0 |
| 5_H10 | BMPR2 | 2 | 3 | 2 | 2 |  |  |  | 0.76 | 1.26 | 1.23 | 1.14 |  |  |  | 1.10 | 0.23 | 0.75 | 0 |
| 5_A11 | PKIB | 2 | 1 | 2 | 3 |  |  |  | 0.76 | 0.42 | 1.23 | 1.71 |  |  |  | 1.03 | 0.56 | 0.55 | 14 |
| 5_B11 | DAPK2 | 3 | 3 | 2 | 2 |  |  |  | 1.14 | 1.26 | 1.23 | 1.14 |  |  |  | 1.19 | 0.06 | 1.04 | 0 |
| 5_C11 | ARAF1 | 3 | 2 | 3 | 2 |  |  |  | 1.14 | 0.84 | 1.85 | 1.14 |  |  |  | 1.24 | 0.43 | 1.19 | 0 |
| 5_D11 | ICK | 2 | 2 | 0 | 0 |  |  |  | 0.76 | 0.84 | 0.00 | 0.00 |  |  |  | 0.40 | 0.46 | -1.37 | 0 |
| 5_E11 | MYO3A | 4 | 3 | 0 | 0 |  |  |  | 1.52 | 1.26 | 0.00 | 0.00 |  |  |  | 0.70 | 0.81 | -0.47 | 20 |
| 5_F11 | PIK3R1 | 4 | 3 | 2 | 2 |  |  |  | 1.52 | 1.26 | 1.23 | 1.14 |  |  |  | 1.29 | 0.16 | 1.33 | 7 |
| 5_G11 | PTK2B | 4 | 3 | 2 | 2 |  |  |  | 1.52 | 1.26 | 1.23 | 1.14 |  |  |  | 1.29 | 0.16 | 1.33 | 16 |
| 5_H11 | NRBP2 | 2 | 1 | 1 | 1 |  |  |  | 0.76 | 0.42 | 0.62 | 0.57 |  |  |  | 0.59 | 0.14 | -0.79 | 18 |
| 6_A1 | NEG | 4 | 4 | 4 | 4 |  |  |  |  |  |  |  |  |  |  |  |  |  |  |
| 6_B1 | NEG | 4 | 4 | 3 | 4 |  |  |  |  |  |  |  |  |  |  |  |  |  |  |
| 6_C1 | NEG | 2 | 2 | 3 | 3 |  |  |  |  |  |  |  |  |  |  |  |  |  |  |
| 6_D1 | NEG | 4 | 4 | 5 | 4 |  |  |  |  |  |  |  |  |  |  |  |  |  |  |
| 6_E1 | NEG | 4 | 4 | 4 | 3 |  |  |  |  |  |  |  |  |  |  |  |  |  |  |
| 6_F1 | NEG | 3 | 3 | 4 | 4 |  |  |  |  |  |  |  |  |  |  |  |  |  |  |
| 6_G1 | NEG | 2 | 3 | 5 | 5 |  |  |  |  |  |  |  |  |  |  |  |  |  |  |
| 6_H1 | NEG | 4 | 4 | 3 | 3 |  |  |  | 3.38 | 3.50 | 3.88 | 3.75 |  |  |  | 3.63 |  |  | 0 |
| 6_A12 | MEK | 3 | 3 | 3 | 3 |  |  |  |  |  |  |  |  |  |  |  |  |  |  |
| 6_B12 | MEK | 3 | 2 | 3 | 3 |  |  |  |  |  |  |  |  |  |  |  |  |  |  |
| 6_C12 | MEK | 3 | 3 | 2 | 2 |  |  |  |  |  |  |  |  |  |  |  |  |  |  |
| 6_D12 | MEK | 0 | 0 | 3 | 3 |  |  |  |  |  |  |  |  |  |  |  |  |  |  |
| 6_E12 | MEK | 2 | 2 | 2 | 2 |  |  |  | 0.65 | 0.57 | 0.67 | 0.69 |  |  |  | 0.65 |  |  | 8 |
| 6_F12 | TOX | 0 | 0 | 0 | 0 |  |  |  |  |  |  |  |  |  |  |  |  |  |  |
| 6_G12 | TOX | 0 | 0 | 0 | 0 |  |  |  |  |  |  |  |  |  |  |  |  |  |  |
| 6_H12 | TOX | 0 | 0 | 0 | 0 |  |  |  | 0.00 | 0.00 | 0.00 | 0.00 |  |  |  | 0.00 |  |  | 100 |
| 6_A2 | MAP2K4 | 1 | 1 | 2 | 2 |  |  |  | 0.30 | 0.29 | 0.52 | 0.53 |  |  |  | 0.41 | 0.14 | -1.35 | 33 |
| 6_B2 | CDK7 | 3 | 4 | 2 | 3 |  |  |  | 0.89 | 1.14 | 0.52 | 0.80 |  |  |  | 0.84 | 0.26 | -0.05 | 9 |
| 6_C2 | STK17A | 2 | 3 | 3 | 4 |  |  |  | 0.59 | 0.86 | 0.77 | 1.07 |  |  |  | 0.82 | 0.20 | -0.09 | 0 |
| 6_D2 | MGC4796 | 4 | 3 | 4 | 5 |  |  |  | 1.19 | 0.86 | 1.03 | 1.33 |  |  |  | 1.10 | 0.20 | 0.76 | 8 |
| 6_E2 | SRPK2 | 4 | 4 | 4 | 5 |  |  |  | 1.19 | 1.14 | 1.03 | 1.33 |  |  |  | 1.17 | 0.12 | 0.98 | 0 |
| 6_F2 | CDKN1A | 2 | 2 | 4 | 3 |  |  |  | 0.59 | 0.57 | 1.03 | 0.80 |  |  |  | 0.75 | 0.22 | -0.31 | 20 |
| 6_G2 | MAST2 | 4 | 4 | 4 | 4 |  |  |  | 1.19 | 1.14 | 1.03 | 1.07 |  |  |  | 1.11 | 0.07 | 0.77 | 2 |
| 6_H2 | TNK1 | 1 | 1 | 2 | 2 |  |  |  | 0.30 | 0.29 | 0.52 | 0.53 |  |  |  | 0.41 | 0.14 | -1.35 | 8 |
| 6_A3 | FLJ13052 | 3 | 2 | 4 | 3 |  |  |  | 0.89 | 0.57 | 1.03 | 0.80 |  |  |  | 0.82 | 0.19 | -0.09 | 10 |
| 6_B3 | LRRK1 | 4 | 3 | 4 | 4 |  |  |  | 1.19 | 0.86 | 1.03 | 1.07 |  |  |  | 1.04 | 0.14 | 0.56 | 0 |
| 6_C3 | TEX14 | 4 | 4 | 4 | 4 |  |  |  | 1.19 | 1.14 | 1.03 | 1.07 |  |  |  | 1.11 | 0.07 | 0.77 | 6 |
| 6_D3 | PKIA | 2 | 3 | 4 | 3 |  |  |  | 0.59 | 0.86 | 1.03 | 0.80 |  |  |  | 0.82 | 0.18 | -0.10 | 1 |
| 6_E3 | CLK2 | 3 | 3 | 1 | 0 |  |  |  | 0.89 | 0.86 | 0.26 | 0.00 |  |  |  | 0.50 | 0.44 | -1.07 | 0 |
| 6_F3 | PANK2 | 4 | 4 | 4 | 5 |  |  |  | 1.19 | 1.14 | 1.03 | 1.33 |  |  |  | 1.17 | 0.12 | 0.98 | 11 |
| 6_G3 | CDK5R1 | 4 | 4 | 3 | 4 |  |  |  | 1.19 | 1.14 | 0.77 | 1.07 |  |  |  | 1.04 | 0.19 | 0.58 | 9 |
| 6_H3 | STK29 | 3 | 3 | 2 | 3 |  |  |  | 0.89 | 0.86 | 0.52 | 0.80 |  |  |  | 0.77 | 0.17 | -0.26 | 11 |
| 6_A4 | TLK2 | 4 | 4 | 6 | 4 |  |  |  | 1.19 | 1.14 | 1.55 | 1.07 |  |  |  | 1.24 | 0.21 | 1.17 | 0 |
| 6_B4 | FLJ25006 | 4 | 3 | 3 | 3 |  |  |  | 1.19 | 0.86 | 0.77 | 0.80 |  |  |  | 0.90 | 0.19 | 0.16 | 4 |
| 6_C4 | MAPKAPK2 | 3 | 3 | 5 | 4 |  |  |  | 0.89 | 0.86 | 1.29 | 1.07 |  |  |  | 1.03 | 0.20 | 0.53 | 25 |
| 6_D4 | RBKS | 2 | 3 | 4 | 4 |  |  |  | 0.59 | 0.86 | 1.03 | 1.07 |  |  |  | 0.89 | 0.22 | 0.11 | 5 |
| 6_E4 | TGFBR2 | 4 | 4 | 4 | 4 |  |  |  | 1.19 | 1.14 | 1.03 | 1.07 |  |  |  | 1.11 | 0.07 | 0.77 | 9 |
| 6_F4 | EPHA3 | 2 | 2 | 4 | 4 |  |  |  | 0.59 | 0.57 | 1.03 | 1.07 |  |  |  | 0.82 | 0.27 | -0.11 | 0 |
| 6_G4 | WEE1 | 4 | 4 | 4 | 4 |  |  |  | 1.19 | 1.14 | 1.03 | 1.07 |  |  |  | 1.11 | 0.07 | 0.77 | 6 |
| 6_H4 | NTRK3 | 4 | 4 | 3 | 3 |  |  |  | 1.19 | 1.14 | 0.77 | 0.80 |  |  |  | 0.98 | 0.22 | 0.37 | 0 |
| 6_A5 | TJP2 | 4 | 4 | 6 | 5 |  |  |  | 1.19 | 1.14 | 1.55 | 1.33 |  |  |  | 1.30 | 0.18 | 1.37 | 18 |
| 6_B5 | MAPK14 | 4 | 4 | 5 | 5 |  |  |  | 1.19 | 1.14 | 1.29 | 1.33 |  |  |  | 1.24 | 0.09 | 1.17 | 14 |
| 6_C5 | LTK | 4 | 4 | 5 | 5 |  |  |  | 1.19 | 1.14 | 1.29 | 1.33 |  |  |  | 1.24 | 0.09 | 1.17 | 4 |
| 6_D5 | PHKA1 | 2 | 2 | 5 | 4 |  |  |  | 0.59 | 0.57 | 1.29 | 1.07 |  |  |  | 0.88 | 0.36 | 0.08 | 5 |
| 6_E5 | STK22B | 3 | 4 | 4 | 4 |  |  |  | 0.89 | 1.14 | 1.03 | 1.07 |  |  |  | 1.03 | 0.11 | 0.55 | 0 |
| 6_F5 | PYCS | 2 | 2 | 3 | 0 |  |  |  | 0.59 | 0.57 | 0.77 | 0.00 |  |  |  | 0.48 | 0.34 | -1.12 | 0 |
| 6_G5 | CDK6 | 3 | 3 | 1 | 2 |  |  |  | 0.89 | 0.86 | 0.26 | 0.53 |  |  |  | 0.63 | 0.30 | -0.66 | 0 |
| 6_H5 | LMTK2 | 3 | 3 | 2 | 3 |  |  |  | 0.89 | 0.86 | 0.52 | 0.80 |  |  |  | 0.77 | 0.17 | -0.26 | 11 |
| 6_A6 | ADCK5 | 0 | 0 | 0 | 0 |  |  |  | 0.00 | 0.00 | 0.00 | 0.00 |  |  |  | 0.00 | 0.00 | -2.59 | 38 |
| 6_B6 | TBK1 | 3 | 4 | 5 | 5 |  |  |  | 0.89 | 1.14 | 1.29 | 1.33 |  |  |  | 1.16 | 0.20 | 0.95 | 2 |
| 6_C6 | CASK | 3 | 3 | 2 | 2 |  |  |  | 0.89 | 0.86 | 0.52 | 0.53 |  |  |  | 0.70 | 0.20 | -0.47 | 10 |
| 6_D6 | ERN1 | 4 | 4 | 0 | 0 |  |  |  | 1.19 | 1.14 | 0.00 | 0.00 |  |  |  | 0.58 | 0.67 | -0.82 | 33 |
| 6_E6 | GAK | 3 | 3 | 0 | 0 |  |  |  | 0.89 | 0.86 | 0.00 | 0.00 |  |  |  | 0.44 | 0.50 | -1.26 | 0 |
| 6_F6 | IHPK1 | 3 | 3 | 3 | 3 |  |  |  | 0.89 | 0.86 | 0.77 | 0.80 |  |  |  | 0.83 | 0.05 | -0.07 | 0 |
| 6_G6 | CDKN2D | 3 | 3 | 4 | 4 |  |  |  | 0.89 | 0.86 | 1.03 | 1.07 |  |  |  | 0.96 | 0.10 | 0.33 | 3 |
| 6_H6 | DTYMK | 4 | 4 | 3 | 2 |  |  |  | 1.19 | 1.14 | 0.77 | 0.53 |  |  |  | 0.91 | 0.31 | 0.17 | 0 |
| 6_A7 | MAPK11 | 3 | 3 | 5 | 5 |  |  |  | 0.89 | 0.86 | 1.29 | 1.33 |  |  |  | 1.09 | 0.25 | 0.73 | 4 |
| 6_B7 | ERN2 | 3 | 2 | 4 | 4 |  |  |  | 0.89 | 0.57 | 1.03 | 1.07 |  |  |  | 0.89 | 0.23 | 0.11 | 21 |
| 6_C7 | DYRK1B | 4 | 3 | 3 | 3 |  |  |  | 1.19 | 0.86 | 0.77 | 0.80 |  |  |  | 0.90 | 0.19 | 0.16 | 18 |
| 6_D7 | STYK1 | 4 | 3 | 3 | 4 |  |  |  | 1.19 | 0.86 | 0.77 | 1.07 |  |  |  | 0.97 | 0.19 | 0.36 | 6 |
| 6_E7 | RPS6KA2 | 0 | 0 | 2 | 1 |  |  |  | 0.00 | 0.00 | 0.52 | 0.27 |  |  |  | 0.20 | 0.25 | -2.00 | 0 |
| 6_F7 | BRD4 | 2 | 2 | 4 | 4 |  |  |  | 0.59 | 0.57 | 1.03 | 1.07 |  |  |  | 0.82 | 0.27 | -0.11 | 0 |
| 6_G7 | PRKCQ | 3 | 3 | 4 | 3 |  |  |  | 0.89 | 0.86 | 1.03 | 0.80 |  |  |  | 0.89 | 0.10 | 0.13 | 9 |
| 6_H7 | PIK3R4 | 4 | 4 | 1 | 1 |  |  |  | 1.19 | 1.14 | 0.26 | 0.27 |  |  |  | 0.71 | 0.52 | -0.42 | 2 |
| 6_A8 | PGK2 | 3 | 3 | 6 | 5 |  |  |  | 0.89 | 0.86 | 1.55 | 1.33 |  |  |  | 1.16 | 0.34 | 0.93 | 14 |
| 6_B8 | PRKCI | 4 | 2 | 4 | 4 |  |  |  | 1.19 | 0.57 | 1.03 | 1.07 |  |  |  | 0.96 | 0.27 | 0.34 | 22 |
| 6_C8 | SBK1 | 4 | 3 | 3 | 3 |  |  |  | 1.19 | 0.86 | 0.77 | 0.80 |  |  |  | 0.90 | 0.19 | 0.16 | 21 |
| 6_D8 | ARK5 | 2 | 3 | 2 | 2 |  |  |  | 0.59 | 0.86 | 0.52 | 0.53 |  |  |  | 0.62 | 0.16 | -0.69 | 37 |
| 6_E8 | MAPK6 | 3 | 0 | 4 | 4 |  |  |  | 0.89 | 0.00 | 1.03 | 1.07 |  |  |  | 0.75 | 0.50 | -0.32 | 0 |
| 6_F8 | TEC | 2 | 2 | 4 | 4 |  |  |  | 0.59 | 0.57 | 1.03 | 1.07 |  |  |  | 0.82 | 0.27 | -0.11 | 0 |
| 6_G8 | PRKAG2 | 4 | 3 | 5 | 5 |  |  |  | 1.19 | 0.86 | 1.29 | 1.33 |  |  |  | 1.17 | 0.22 | 0.96 | 36 |
| 6_H8 | GCK | 4 | 4 | 2 | 2 |  |  |  | 1.19 | 1.14 | 0.52 | 0.53 |  |  |  | 0.84 | 0.37 | -0.02 | 11 |
| 6_A9 | BUB1B | 2 | 4 | 5 | 5 |  |  |  | 0.59 | 1.14 | 1.29 | 1.33 |  |  |  | 1.09 | 0.34 | 0.72 | 12 |
| 6_B9 | IKBKE | 2 | 4 | 3 | 3 |  |  |  | 0.59 | 1.14 | 0.77 | 0.80 |  |  |  | 0.83 | 0.23 | -0.08 | 24 |
| 6_C9 | PCTK1 | 1 | 4 | 4 | 5 |  |  |  | 0.30 | 1.14 | 1.03 | 1.33 |  |  |  | 0.95 | 0.45 | 0.30 | 37 |
| 6_D9 | PIP5K1B | 4 | 2 | 4 | 4 |  |  |  | 1.19 | 0.57 | 1.03 | 1.07 |  |  |  | 0.96 | 0.27 | 0.34 | 22 |
| 6_E9 | GRK7 | 4 | 2 | 3 | 3 |  |  |  | 1.19 | 0.57 | 0.77 | 0.80 |  |  |  | 0.83 | 0.26 | -0.06 | 11 |
| 6_F9 | FRDA | 4 | 2 | 2 | 3 |  |  |  | 1.19 | 0.57 | 0.52 | 0.80 |  |  |  | 0.77 | 0.30 | -0.26 | 9 |
| 6_G9 | MAP3K3 | 3 | 4 | 1 | 1 |  |  |  | 0.89 | 1.14 | 0.26 | 0.27 |  |  |  | 0.64 | 0.45 | -0.65 | 9 |
| 6_H9 | EPHB2 | 4 | 4 | 2 | 2 |  |  |  | 1.19 | 1.14 | 0.52 | 0.53 |  |  |  | 0.84 | 0.37 | -0.02 | 21 |
| 6_A10 | MAP2K5 | 2 | 3 | 3 | 3 |  |  |  | 0.59 | 0.86 | 0.77 | 0.80 |  |  |  | 0.76 | 0.11 | -0.29 | 11 |
| 6_B10 | CSNK1A1L | 4 | 2 | 4 | 5 |  |  |  | 1.19 | 0.57 | 1.03 | 1.33 |  |  |  | 1.03 | 0.33 | 0.54 | 0 |
| 6_C10 | MAP3K4 | 3 | 2 | 4 | 5 |  |  |  | 0.89 | 0.57 | 1.03 | 1.33 |  |  |  | 0.96 | 0.32 | 0.32 | 33 |
| 6_D10 | DGUOK | 2 | 4 | 3 | 3 |  |  |  | 0.59 | 1.14 | 0.77 | 0.80 |  |  |  | 0.83 | 0.23 | -0.08 | 0 |
| 6_E10 | ADRBK1 | 3 | 4 | 5 | 5 |  |  |  | 0.89 | 1.14 | 1.29 | 1.33 |  |  |  | 1.16 | 0.20 | 0.95 | 0 |
| 6_F10 | PRPS2 | 3 | 4 | 3 | 3 |  |  |  | 0.89 | 1.14 | 0.77 | 0.80 |  |  |  | 0.90 | 0.17 | 0.15 | 18 |
| 6_G10 | FGFRL1 | 3 | 4 | 4 | 4 |  |  |  | 0.89 | 1.14 | 1.03 | 1.07 |  |  |  | 1.03 | 0.11 | 0.55 | 9 |
| 6_H10 | SGKL | 0 | 0 | 1 | 1 |  |  |  | 0.00 | 0.00 | 0.26 | 0.27 |  |  |  | 0.13 | 0.15 | -2.19 | 2 |
| 6_A11 | LAK | 2 | 1 | 0 | 0 |  |  |  | 0.59 | 0.29 | 0.00 | 0.00 |  |  |  | 0.22 | 0.28 | -1.92 | 14 |
| 6_B11 | PIP5K1A | 2 | 3 | 3 | 4 |  |  |  | 0.59 | 0.86 | 0.77 | 1.07 |  |  |  | 0.82 | 0.20 | -0.09 | 0 |
| 6_C11 | STK38L | 3 | 2 | 3 | 3 |  |  |  | 0.89 | 0.57 | 0.77 | 0.80 |  |  |  | 0.76 | 0.13 | -0.28 | 20 |
| 6_D11 | PANK3 | 2 | 3 | 5 | 4 |  |  |  | 0.59 | 0.86 | 1.29 | 1.07 |  |  |  | 0.95 | 0.30 | 0.30 | 0 |
| 6_E11 | CDK10 | 2 | 2 | 3 | 3 |  |  |  | 0.59 | 0.57 | 0.77 | 0.80 |  |  |  | 0.68 | 0.12 | -0.51 | 47 |
| 6_F11 | AK5 | 3 | 3 | 4 | 4 |  |  |  | 0.89 | 0.86 | 1.03 | 1.07 |  |  |  | 0.96 | 0.10 | 0.33 | 36 |
| 6_G11 | NME1 | 4 | 4 | 4 | 3 |  |  |  | 1.19 | 1.14 | 1.03 | 0.80 |  |  |  | 1.04 | 0.17 | 0.57 | 6 |
| 6_H11 | DLG3 | 3 | 3 | 2 | 2 |  |  |  | 0.89 | 0.86 | 0.52 | 0.53 |  |  |  | 0.70 | 0.20 | -0.47 | 3 |
| 7_A1 | NEG | 4 | 4 | 4 | 3 |  |  |  |  |  |  |  |  |  |  |  |  |  |  |
| 7_B1 | NEG | 4 | 4 | 4 | 4 |  |  |  |  |  |  |  |  |  |  |  |  |  |  |
| 7_C1 | NEG | 3 | 3 | 4 | 4 |  |  |  |  |  |  |  |  |  |  |  |  |  |  |
| 7_D1 | NEG | 3 | 2 | 5 | 5 |  |  |  |  |  |  |  |  |  |  |  |  |  |  |
| 7_E1 | NEG | 4 | 4 | 5 | 5 |  |  |  |  |  |  |  |  |  |  |  |  |  |  |
| 7_F1 | NEG | 4 | 4 | 5 | 5 |  |  |  |  |  |  |  |  |  |  |  |  |  |  |
| 7_G1 | NEG | 3 | 3 | 5 | 5 |  |  |  |  |  |  |  |  |  |  |  |  |  |  |
| 7_H1 | NEG | 4 | 4 | 5 | 5 |  |  |  | 3.63 | 3.50 | 4.63 | 4.50 |  |  |  | 4.06 |  |  | 0 |
| 7_A12 | MEK | 2 | 2 | 3 | 3 |  |  |  |  |  |  |  |  |  |  |  |  |  |  |
| 7_B12 | MEK | 2 | 3 | 3 | 4 |  |  |  |  |  |  |  |  |  |  |  |  |  |  |
| 7_C12 | MEK | 3 | 4 | 3 | 3 |  |  |  |  |  |  |  |  |  |  |  |  |  |  |
| 7_D12 | MEK | 1 | 1 | 3 | 4 |  |  |  |  |  |  |  |  |  |  |  |  |  |  |
| 7_E12 | MEK | 4 | 3 | 3 | 4 |  |  |  | 0.66 | 0.74 | 0.65 | 0.80 |  |  |  | 0.71 |  |  | 0 |
| 7_F12 | TOX | 0 | 0 | 0 | 0 |  |  |  |  |  |  |  |  |  |  |  |  |  |  |
| 7_G12 | TOX | 0 | 0 | 0 | 0 |  |  |  |  |  |  |  |  |  |  |  |  |  |  |
| 7_H12 | TOX | 0 | 0 | 0 | 0 |  |  |  | 0.00 | 0.00 | 0.00 | 0.00 |  |  |  | 0.00 |  |  | 100 |
| 7_A2 | RET | 2 | 2 | 3 | 4 |  |  |  | 0.55 | 0.57 | 0.65 | 0.89 |  |  |  | 0.67 | 0.15 | -0.57 | 15 |
| 7_B2 | CDC2L5 | 1 | 0 | 0 | 0 |  |  |  | 0.28 | 0.00 | 0.00 | 0.00 |  |  |  | 0.07 | 0.14 | -2.38 | 2 |
| 7_C2 | KCNH2 | 3 | 3 | 5 | 6 |  |  |  | 0.83 | 0.86 | 1.08 | 1.33 |  |  |  | 1.02 | 0.23 | 0.52 | 5 |
| 7_D2 | PRKCD | 0 | 1 | 3 | 3 |  |  |  | 0.00 | 0.29 | 0.65 | 0.67 |  |  |  | 0.40 | 0.32 | -1.37 | 0 |
| 7_E2 | STK32B | 4 | 4 | 4 | 5 |  |  |  | 1.10 | 1.14 | 0.86 | 1.11 |  |  |  | 1.06 | 0.13 | 0.62 | 0 |
| 7_F2 | PKMYT1 | 4 | 4 | 5 | 5 |  |  |  | 1.10 | 1.14 | 1.08 | 1.11 |  |  |  | 1.11 | 0.03 | 0.78 | 2 |
| 7_G2 | SLK | 4 | 4 | 5 | 5 |  |  |  | 1.10 | 1.14 | 1.08 | 1.11 |  |  |  | 1.11 | 0.03 | 0.78 | 0 |
| 7_H2 | PRKD2 | 1 | 1 | 4 | 4 |  |  |  | 0.28 | 0.29 | 0.86 | 0.89 |  |  |  | 0.58 | 0.34 | -0.83 | 0 |
| 7_A3 | MAP2K6 | 3 | 2 | 4 | 5 |  |  |  | 0.83 | 0.57 | 0.86 | 1.11 |  |  |  | 0.84 | 0.22 | -0.03 | 0 |
| 7_B3 | ALK | 4 | 4 | 4 | 4 |  |  |  | 1.10 | 1.14 | 0.86 | 0.89 |  |  |  | 1.00 | 0.14 | 0.45 | 24 |
| 7_C3 | CDKL1 | 4 | 4 | 0 | 0 |  |  |  | 1.10 | 1.14 | 0.00 | 0.00 |  |  |  | 0.56 | 0.65 | -0.88 | 0 |
| 7_D3 | ATR | 4 | 3 | 4 | 3 |  |  |  | 1.10 | 0.86 | 0.86 | 0.67 |  |  |  | 0.87 | 0.18 | 0.06 | 20 |
| 7_E3 | MAP3K7IP1 | 4 | 4 | 4 | 4 |  |  |  | 1.10 | 1.14 | 0.86 | 0.89 |  |  |  | 1.00 | 0.14 | 0.45 | 0 |
| 7_F3 | PACE-1 | 2 | 3 | 5 | 4 |  |  |  | 0.55 | 0.86 | 1.08 | 0.89 |  |  |  | 0.84 | 0.22 | -0.02 | 4 |
| 7_G3 | CDKN1C | 2 | 2 | 4 | 4 |  |  |  | 0.55 | 0.57 | 0.86 | 0.89 |  |  |  | 0.72 | 0.18 | -0.40 | 0 |
| 7_H3 | CAMKK1 | 4 | 3 | 5 | 5 |  |  |  | 1.10 | 0.86 | 1.08 | 1.11 |  |  |  | 1.04 | 0.12 | 0.57 | 0 |
| 7_A4 | RPS6KC1 | 4 | 4 | 5 | 5 |  |  |  | 1.10 | 1.14 | 1.08 | 1.11 |  |  |  | 1.11 | 0.03 | 0.78 | 14 |
| 7_B4 | SPEG | 4 | 4 | 3 | 4 |  |  |  | 1.10 | 1.14 | 0.65 | 0.89 |  |  |  | 0.95 | 0.23 | 0.28 | 5 |
| 7_C4 | NTRK2 | 1 | 1 | 3 | 5 |  |  |  | 0.28 | 0.29 | 0.65 | 1.11 |  |  |  | 0.58 | 0.39 | -0.83 | 8 |
| 7_D4 | CAMK2A | 4 | 4 | 4 | 6 |  |  |  | 1.10 | 1.14 | 0.86 | 1.33 |  |  |  | 1.11 | 0.19 | 0.79 | 3 |
| 7_E4 | PRKR | 3 | 3 | 5 | 6 |  |  |  | 0.83 | 0.86 | 1.08 | 1.33 |  |  |  | 1.02 | 0.23 | 0.52 | 0 |
| 7_F4 | IKBKB | 3 | 3 | 3 | 3 |  |  |  | 0.83 | 0.86 | 0.65 | 0.67 |  |  |  | 0.75 | 0.11 | -0.31 | 5 |
| 7_G4 | PIP5K1C | 4 | 4 | 4 | 4 |  |  |  | 1.10 | 1.14 | 0.86 | 0.89 |  |  |  | 1.00 | 0.14 | 0.45 | 0 |
| 7_H4 | LYK5 | 4 | 4 | 4 | 4 |  |  |  | 1.10 | 1.14 | 0.86 | 0.89 |  |  |  | 1.00 | 0.14 | 0.45 | 0 |
| 7_A5 | NME7 | 4 | 4 | 6 | 5 |  |  |  | 1.10 | 1.14 | 1.30 | 1.11 |  |  |  | 1.16 | 0.09 | 0.95 | 1 |
| 7_B5 | TP53RK | 3 | 3 | 4 | 5 |  |  |  | 0.83 | 0.86 | 0.86 | 1.11 |  |  |  | 0.92 | 0.13 | 0.19 | 8 |
| 7_C5 | AATK | 4 | 3 | 4 | 6 |  |  |  | 1.10 | 0.86 | 0.86 | 1.33 |  |  |  | 1.04 | 0.23 | 0.57 | 0 |
| 7_D5 | KDR | 3 | 3 | 4 | 4 |  |  |  | 0.83 | 0.86 | 0.86 | 0.89 |  |  |  | 0.86 | 0.03 | 0.02 | 0 |
| 7_E5 | RIPK3 | 0 | 0 | 3 | 4 |  |  |  | 0.00 | 0.00 | 0.65 | 0.89 |  |  |  | 0.38 | 0.45 | -1.42 | 0 |
| 7_F5 | MAP3K13 | 2 | 3 | 3 | 5 |  |  |  | 0.55 | 0.86 | 0.65 | 1.11 |  |  |  | 0.79 | 0.25 | -0.18 | 0 |
| 7_G5 | PFKL | 2 | 3 | 6 | 7 |  |  |  | 0.55 | 0.86 | 1.30 | 1.56 |  |  |  | 1.07 | 0.45 | 0.65 | 5 |
| 7_H5 | COMMD3 | 4 | 4 | 4 | 4 |  |  |  | 1.10 | 1.14 | 0.86 | 0.89 |  |  |  | 1.00 | 0.14 | 0.45 | 0 |
| 7_A6 | MAP3K10 | 4 | 4 | 6 | 5 |  |  |  | 1.10 | 1.14 | 1.30 | 1.11 |  |  |  | 1.16 | 0.09 | 0.95 | 14 |
| 7_B6 | EFNA5 | 3 | 2 | 3 | 5 |  |  |  | 0.83 | 0.57 | 0.65 | 1.11 |  |  |  | 0.79 | 0.24 | -0.19 | 0 |
| 7_C6 | PRKCG | 2 | 2 | 5 | 6 |  |  |  | 0.55 | 0.57 | 1.08 | 1.33 |  |  |  | 0.88 | 0.39 | 0.10 | 21 |
| 7_D6 | TAF1 | 3 | 3 | 4 | 4 |  |  |  | 0.83 | 0.86 | 0.86 | 0.89 |  |  |  | 0.86 | 0.03 | 0.02 | 0 |
| 7_E6 | ULK4 | 4 | 4 | 6 | 5 |  |  |  | 1.10 | 1.14 | 1.30 | 1.11 |  |  |  | 1.16 | 0.09 | 0.95 | 0 |
| 7_F6 | CAMK1D | 4 | 4 | 4 | 5 |  |  |  | 1.10 | 1.14 | 0.86 | 1.11 |  |  |  | 1.06 | 0.13 | 0.62 | 0 |
| 7_G6 | PCK1 | 4 | 4 | 4 | 3 |  |  |  | 1.10 | 1.14 | 0.86 | 0.67 |  |  |  | 0.94 | 0.22 | 0.28 | 0 |
| 7_H6 | TK2 | 2 | 2 | 4 | 4 |  |  |  | 0.55 | 0.57 | 0.86 | 0.89 |  |  |  | 0.72 | 0.18 | -0.40 | 0 |
| 7_A7 | UCK1 | 4 | 4 | 4 | 4 |  |  |  | 1.10 | 1.14 | 0.86 | 0.89 |  |  |  | 1.00 | 0.14 | 0.45 | 9 |
| 7_B7 | MAPK13 | 3 | 3 | 4 | 5 |  |  |  | 0.83 | 0.86 | 0.86 | 1.11 |  |  |  | 0.92 | 0.13 | 0.19 | 3 |
| 7_C7 | STK38 | 3 | 2 | 1 | 2 |  |  |  | 0.83 | 0.57 | 0.22 | 0.44 |  |  |  | 0.51 | 0.26 | -1.03 | 0 |
| 7_D7 | CDK11 | 4 | 4 | 6 | 6 |  |  |  | 1.10 | 1.14 | 1.30 | 1.33 |  |  |  | 1.22 | 0.11 | 1.12 | 0 |
| 7_E7 | SPHK2 | 4 | 3 | 4 | 4 |  |  |  | 1.10 | 0.86 | 0.86 | 0.89 |  |  |  | 0.93 | 0.12 | 0.23 | 0 |
| 7_F7 | IRAK3 | 0 | 0 | 4 | 4 |  |  |  | 0.00 | 0.00 | 0.86 | 0.89 |  |  |  | 0.44 | 0.51 | -1.26 | 0 |
| 7_G7 | CDK9 | 4 | 3 | 4 | 5 |  |  |  | 1.10 | 0.86 | 0.86 | 1.11 |  |  |  | 0.98 | 0.14 | 0.40 | 0 |
| 7_H7 | CSNK2A1 | 4 | 4 | 6 | 6 |  |  |  | 1.10 | 1.14 | 1.30 | 1.33 |  |  |  | 1.22 | 0.11 | 1.12 | 0 |
| 7_A8 | FGFR2 | 3 | 2 | 5 | 4 |  |  |  | 0.83 | 0.57 | 1.08 | 0.89 |  |  |  | 0.84 | 0.21 | -0.03 | 18 |
| 7_B8 | TRIB2 | 4 | 4 | 0 | 0 |  |  |  | 1.10 | 1.14 | 0.00 | 0.00 |  |  |  | 0.56 | 0.65 | -0.88 | 8 |
| 7_C8 | PMVK | 4 | 4 | 5 | 4 |  |  |  | 1.10 | 1.14 | 1.08 | 0.89 |  |  |  | 1.05 | 0.11 | 0.61 | 8 |
| 7_D8 | SNARK | 4 | 4 | 4 | 4 |  |  |  | 1.10 | 1.14 | 0.86 | 0.89 |  |  |  | 1.00 | 0.14 | 0.45 | 1 |
| 7_E8 | TESK1 | 2 | 2 | 2 | 2 |  |  |  | 0.55 | 0.57 | 0.43 | 0.44 |  |  |  | 0.50 | 0.07 | -1.07 | 14 |
| 7_F8 | MOS | 3 | 4 | 4 | 3 |  |  |  | 0.83 | 1.14 | 0.86 | 0.67 |  |  |  | 0.88 | 0.20 | 0.07 | 0 |
| 7_G8 | FGFR4 | 4 | 4 | 4 | 3 |  |  |  | 1.10 | 1.14 | 0.86 | 0.67 |  |  |  | 0.94 | 0.22 | 0.28 | 3 |
| 7_H8 | SRMS | 0 | 0 | 4 | 4 |  |  |  | 0.00 | 0.00 | 0.86 | 0.89 |  |  |  | 0.44 | 0.51 | -1.26 | 0 |
| 7_A9 | ERBB3 | 4 | 4 | 5 | 4 |  |  |  | 1.10 | 1.14 | 1.08 | 0.89 |  |  |  | 1.05 | 0.11 | 0.61 | 39 |
| 7_B9 | DGKH | 2 | 4 | 5 | 5 |  |  |  | 0.55 | 1.14 | 1.08 | 1.11 |  |  |  | 0.97 | 0.28 | 0.36 | 13 |
| 7_C9 | EPHA1 | 0 | 0 | 5 | 4 |  |  |  | 0.00 | 0.00 | 1.08 | 0.89 |  |  |  | 0.49 | 0.57 | -1.09 | 0 |
| 7_D9 | SCYL1 | 4 | 4 | 5 | 4 |  |  |  | 1.10 | 1.14 | 1.08 | 0.89 |  |  |  | 1.05 | 0.11 | 0.61 | 0 |
| 7_E9 | CSNK2B | 4 | 4 | 5 | 4 |  |  |  | 1.10 | 1.14 | 1.08 | 0.89 |  |  |  | 1.05 | 0.11 | 0.61 | 9 |
| 7_F9 | PIP5K2B | 4 | 3 | 4 | 4 |  |  |  | 1.10 | 0.86 | 0.86 | 0.89 |  |  |  | 0.93 | 0.12 | 0.23 | 0 |
| 7_G9 | CRKL | 4 | 4 | 6 | 4 |  |  |  | 1.10 | 1.14 | 1.30 | 0.89 |  |  |  | 1.11 | 0.17 | 0.78 | 1 |
| 7_H9 | MAP4K2 | 2 | 3 | 4 | 4 |  |  |  | 0.55 | 0.86 | 0.86 | 0.89 |  |  |  | 0.79 | 0.16 | -0.19 | 0 |
| 7_A10 | NEK3 | 1 | 0 | 1 | 0 |  |  |  | 0.28 | 0.00 | 0.22 | 0.00 |  |  |  | 0.12 | 0.14 | -2.22 | 14 |
| 7_B10 | FRK | 3 | 3 | 4 | 3 |  |  |  | 0.83 | 0.86 | 0.86 | 0.67 |  |  |  | 0.80 | 0.09 | -0.15 | 7 |
| 7_C10 | RAF1 | 3 | 3 | 4 | 5 |  |  |  | 0.83 | 0.86 | 0.86 | 1.11 |  |  |  | 0.92 | 0.13 | 0.19 | 4 |
| 7_D10 | PRKAB2 | 3 | 3 | 1 | 3 |  |  |  | 0.83 | 0.86 | 0.22 | 0.67 |  |  |  | 0.64 | 0.30 | -0.64 | 0 |
| 7_E10 | ERBB4 | 0 | 0 | 0 | 0 |  |  |  | 0.00 | 0.00 | 0.00 | 0.00 |  |  |  | 0.00 | 0.00 | -2.59 | 10 |
| 7_F10 | STK4 | 4 | 4 | 6 | 6 |  |  |  | 1.10 | 1.14 | 1.30 | 1.33 |  |  |  | 1.22 | 0.11 | 1.12 | 1 |
| 7_G10 | RPS6KA5 | 4 | 4 | 4 | 3 |  |  |  | 1.10 | 1.14 | 0.86 | 0.67 |  |  |  | 0.94 | 0.22 | 0.28 | 5 |
| 7_H10 | KIAA1811 | 4 | 4 | 5 | 5 |  |  |  | 1.10 | 1.14 | 1.08 | 1.11 |  |  |  | 1.11 | 0.03 | 0.78 | 0 |
| 7_A11 | COL4A3BP | 3 | 4 | 4 | 4 |  |  |  | 0.83 | 1.14 | 0.86 | 0.89 |  |  |  | 0.93 | 0.14 | 0.24 | 42 |
| 7_B11 | PIK3CB | 1 | 2 | 1 | 3 |  |  |  | 0.28 | 0.57 | 0.22 | 0.67 |  |  |  | 0.43 | 0.22 | -1.28 | 0 |
| 7_C11 | PRKCSH | 4 | 4 | 5 | 4 |  |  |  | 1.10 | 1.14 | 1.08 | 0.89 |  |  |  | 1.05 | 0.11 | 0.61 | 0 |
| 7_D11 | MGC4796 | 4 | 4 | 2 | 2 |  |  |  | 1.10 | 1.14 | 0.43 | 0.44 |  |  |  | 0.78 | 0.40 | -0.22 | 0 |
| 7_E11 | PFKFB4 | 4 | 4 | 0 | 0 |  |  |  | 1.10 | 1.14 | 0.00 | 0.00 |  |  |  | 0.56 | 0.65 | -0.88 | 0 |
| 7_F11 | BAIAP1 | 1 | 0 | 4 | 4 |  |  |  | 0.28 | 0.00 | 0.86 | 0.89 |  |  |  | 0.51 | 0.44 | -1.05 | 0 |
| 7_G11 | STK32C | 4 | 4 | 5 | 6 |  |  |  | 1.10 | 1.14 | 1.08 | 1.33 |  |  |  | 1.17 | 0.11 | 0.95 | 0 |
| 7_H11 | PDK1 | 4 | 3 | 0 | 0 |  |  |  | 1.10 | 0.86 | 0.00 | 0.00 |  |  |  | 0.49 | 0.57 | -1.10 | 0 |
| 8_A1 | NEG | 4 | 4 | 4 | 4 |  |  |  |  |  |  |  |  |  |  |  |  |  |  |
| 8_B1 | NEG | 4 | 4 | 3 | 4 |  |  |  |  |  |  |  |  |  |  |  |  |  |  |
| 8_C1 | NEG | 4 | 4 | 4 | 3 |  |  |  |  |  |  |  |  |  |  |  |  |  |  |
| 8_D1 | NEG | 4 | 4 | 4 | 4 |  |  |  |  |  |  |  |  |  |  |  |  |  |  |
| 8_E1 | NEG | 4 | 4 | 5 | 4 |  |  |  |  |  |  |  |  |  |  |  |  |  |  |
| 8_F1 | NEG | 4 | 4 | 4 | 4 |  |  |  |  |  |  |  |  |  |  |  |  |  |  |
| 8_G1 | NEG | 4 | 4 | 4 | 4 |  |  |  |  |  |  |  |  |  |  |  |  |  |  |
| 8_H1 | NEG | 4 | 4 | 4 | 4 |  |  |  | 4.00 | 4.00 | 4.00 | 3.88 |  |  |  | 3.97 |  |  | 0 |
| 8_A12 | MEK | 1 | 1 | 0 | 0 |  |  |  |  |  |  |  |  |  |  |  |  |  |  |
| 8_B12 | MEK | 4 | 4 | 2 | 1 |  |  |  |  |  |  |  |  |  |  |  |  |  |  |
| 8_C12 | MEK | 3 | 4 | 2 | 2 |  |  |  |  |  |  |  |  |  |  |  |  |  |  |
| 8_D12 | MEK | 3 | 4 | 2 | 3 |  |  |  |  |  |  |  |  |  |  |  |  |  |  |
| 8_E12 | MEK | 3 | 3 | 2 | 3 |  |  |  | 0.70 | 0.80 | 0.40 | 0.46 |  |  |  | 0.59 |  |  | 20 |
| 8_F12 | TOX | 0 | 0 | 0 | 0 |  |  |  |  |  |  |  |  |  |  |  |  |  |  |
| 8_G12 | TOX | 0 | 0 | 0 | 0 |  |  |  |  |  |  |  |  |  |  |  |  |  |  |
| 8_H12 | TOX | 0 | 0 | 0 | 0 |  |  |  | 0.00 | 0.00 | 0.00 | 0.00 |  |  |  | 0.00 |  |  | 100 |
| 8_A2 | MELK | 4 | 4 | 5 | 5 |  |  |  | 1.00 | 1.00 | 1.25 | 1.29 |  |  |  | 1.14 | 0.16 | 0.86 | 0 |
| 8_B2 | TGFBR3 | 4 | 4 | 2 | 3 |  |  |  | 1.00 | 1.00 | 0.50 | 0.77 |  |  |  | 0.82 | 0.24 | -0.10 | 3 |
| 8_C2 | CSNK1A1 | 3 | 3 | 4 | 3 |  |  |  | 0.75 | 0.75 | 1.00 | 0.77 |  |  |  | 0.82 | 0.12 | -0.10 | 7 |
| 8_D2 | JIK | 4 | 4 | 2 | 2 |  |  |  | 1.00 | 1.00 | 0.50 | 0.52 |  |  |  | 0.75 | 0.28 | -0.30 | 0 |
| 8_E2 | PTK9L | 4 | 4 | 5 | 4 |  |  |  | 1.00 | 1.00 | 1.25 | 1.03 |  |  |  | 1.07 | 0.12 | 0.66 | 5 |
| 8_F2 | PIM3 | 4 | 4 | 4 | 6 |  |  |  | 1.00 | 1.00 | 1.00 | 1.55 |  |  |  | 1.14 | 0.27 | 0.87 | 0 |
| 8_G2 | PXK | 4 | 4 | 3 | 3 |  |  |  | 1.00 | 1.00 | 0.75 | 0.77 |  |  |  | 0.88 | 0.14 | 0.09 | 0 |
| 8_H2 | SAST | 4 | 3 | 3 | 2 |  |  |  | 1.00 | 0.75 | 0.75 | 0.52 |  |  |  | 0.75 | 0.20 | -0.30 | 0 |
| 8_A3 | PRKCH | 1 | 0 | 2 | 2 |  |  |  | 0.25 | 0.00 | 0.50 | 0.52 |  |  |  | 0.32 | 0.24 | -1.63 | 9 |
| 8_B3 | UMP-CMPK | 4 | 4 | 6 | 4 |  |  |  | 1.00 | 1.00 | 1.50 | 1.03 |  |  |  | 1.13 | 0.25 | 0.85 | 0 |
| 8_C3 | NME2 | 3 | 3 | 2 | 3 |  |  |  | 0.75 | 0.75 | 0.50 | 0.77 |  |  |  | 0.69 | 0.13 | -0.48 | 3 |
| 8_D3 | PKLR | 4 | 4 | 5 | 4 |  |  |  | 1.00 | 1.00 | 1.25 | 1.03 |  |  |  | 1.07 | 0.12 | 0.66 | 11 |
| 8_E3 | PIK3C2A | 2 | 3 | 6 | 4 |  |  |  | 0.50 | 0.75 | 1.50 | 1.03 |  |  |  | 0.95 | 0.43 | 0.28 | 90 |
| 8_F3 | LMTK3 | 3 | 3 | 1 | 1 |  |  |  | 0.75 | 0.75 | 0.25 | 0.26 |  |  |  | 0.50 | 0.29 | -1.07 | 0 |
| 8_G3 | HUNK | 3 | 2 | 2 | 3 |  |  |  | 0.75 | 0.50 | 0.50 | 0.77 |  |  |  | 0.63 | 0.15 | -0.67 | 0 |
| 8_H3 | NEK2 | 4 | 3 | 4 | 4 |  |  |  | 1.00 | 0.75 | 1.00 | 1.03 |  |  |  | 0.95 | 0.13 | 0.28 | 0 |
| 8_A4 | PAK3 | 4 | 4 | 5 | 3 |  |  |  | 1.00 | 1.00 | 1.25 | 0.77 |  |  |  | 1.01 | 0.19 | 0.47 | 4 |
| 8_B4 | CHKA | 3 | 3 | 2 | 4 |  |  |  | 0.75 | 0.75 | 0.50 | 1.03 |  |  |  | 0.76 | 0.22 | -0.29 | 0 |
| 8_C4 | MAK | 3 | 4 | 3 | 2 |  |  |  | 0.75 | 1.00 | 0.75 | 0.52 |  |  |  | 0.75 | 0.20 | -0.30 | 26 |
| 8_D4 | PAK7 | 4 | 4 | 5 | 6 |  |  |  | 1.00 | 1.00 | 1.25 | 1.55 |  |  |  | 1.20 | 0.26 | 1.06 | 2 |
| 8_E4 | STK32A | 4 | 4 | 4 | 4 |  |  |  | 1.00 | 1.00 | 1.00 | 1.03 |  |  |  | 1.01 | 0.02 | 0.47 | 7 |
| 8_F4 | CHEK2 | 4 | 4 | 4 | 5 |  |  |  | 1.00 | 1.00 | 1.00 | 1.29 |  |  |  | 1.07 | 0.15 | 0.67 | 0 |
| 8_G4 | MAP4K1 | 1 | 3 | 4 | 4 |  |  |  | 0.25 | 0.75 | 1.00 | 1.03 |  |  |  | 0.76 | 0.36 | -0.29 | 5 |
| 8_H4 | MAPKAPK5 | 4 | 4 | 3 | 3 |  |  |  | 1.00 | 1.00 | 0.75 | 0.77 |  |  |  | 0.88 | 0.14 | 0.09 | 8 |
| 8_A5 | PFKFB1 | 3 | 3 | 2 | 3 |  |  |  | 0.75 | 0.75 | 0.50 | 0.77 |  |  |  | 0.69 | 0.13 | -0.48 | 10 |
| 8_B5 | WNK4 | 4 | 4 | 4 | 4 |  |  |  | 1.00 | 1.00 | 1.00 | 1.03 |  |  |  | 1.01 | 0.02 | 0.47 | 9 |
| 8_C5 | PIK3CD | 4 | 4 | 3 | 2 |  |  |  | 1.00 | 1.00 | 0.75 | 0.52 |  |  |  | 0.82 | 0.23 | -0.11 | 19 |
| 8_D5 | VRK1 | 4 | 4 | 5 | 5 |  |  |  | 1.00 | 1.00 | 1.25 | 1.29 |  |  |  | 1.14 | 0.16 | 0.86 | 6 |
| 8_E5 | GTF2H1 | 1 | 2 | 1 | 3 |  |  |  | 0.25 | 0.50 | 0.25 | 0.77 |  |  |  | 0.44 | 0.25 | -1.24 | 16 |
| 8_F5 | JAK1 | 4 | 4 | 5 | 5 |  |  |  | 1.00 | 1.00 | 1.25 | 1.29 |  |  |  | 1.14 | 0.16 | 0.86 | 0 |
| 8_G5 | PCTK2 | 3 | 3 | 3 | 3 |  |  |  | 0.75 | 0.75 | 0.75 | 0.77 |  |  |  | 0.76 | 0.01 | -0.29 | 1 |
| 8_H5 | NRK | 4 | 4 | 4 | 5 |  |  |  | 1.00 | 1.00 | 1.00 | 1.29 |  |  |  | 1.07 | 0.15 | 0.67 | 11 |
| 8_A6 | INSRR | 3 | 3 | 2 | 2 |  |  |  | 0.75 | 0.75 | 0.50 | 0.52 |  |  |  | 0.63 | 0.14 | -0.68 | 0 |
| 8_B6 | RPS6KA1 | 3 | 3 | 3 | 3 |  |  |  | 0.75 | 0.75 | 0.75 | 0.77 |  |  |  | 0.76 | 0.01 | -0.29 | 1 |
| 8_C6 | MAP3K6 | 4 | 4 | 4 | 4 |  |  |  | 1.00 | 1.00 | 1.00 | 1.03 |  |  |  | 1.01 | 0.02 | 0.47 | 4 |
| 8_D6 | EPHA6 | 0 | 0 | 1 | 2 |  |  |  | 0.00 | 0.00 | 0.25 | 0.52 |  |  |  | 0.19 | 0.25 | -2.01 | 0 |
| 8_E6 | TOPK | 4 | 3 | 5 | 4 |  |  |  | 1.00 | 0.75 | 1.25 | 1.03 |  |  |  | 1.01 | 0.20 | 0.47 | 0 |
| 8_F6 | EIF2AK3 | 3 | 4 | 3 | 3 |  |  |  | 0.75 | 1.00 | 0.75 | 0.77 |  |  |  | 0.82 | 0.12 | -0.10 | 4 |
| 8_G6 | INSR | 2 | 2 | 2 | 2 |  |  |  | 0.50 | 0.50 | 0.50 | 0.52 |  |  |  | 0.50 | 0.01 | -1.06 | 0 |
| 8_H6 | PFKFB2 | 4 | 4 | 5 | 4 |  |  |  | 1.00 | 1.00 | 1.25 | 1.03 |  |  |  | 1.07 | 0.12 | 0.66 | 11 |
| 8_A7 | DLG2 | 4 | 4 | 2 | 3 |  |  |  | 1.00 | 1.00 | 0.50 | 0.77 |  |  |  | 0.82 | 0.24 | -0.10 | 1 |
| 8_B7 | IHPK3 | 4 | 4 | 5 | 3 |  |  |  | 1.00 | 1.00 | 1.25 | 0.77 |  |  |  | 1.01 | 0.19 | 0.47 | 13 |
| 8_C7 | BRDT | 1 | 2 | 3 | 4 |  |  |  | 0.25 | 0.50 | 0.75 | 1.03 |  |  |  | 0.63 | 0.34 | -0.67 | 7 |
| 8_D7 | GUCY2C | 4 | 4 | 4 | 4 |  |  |  | 1.00 | 1.00 | 1.00 | 1.03 |  |  |  | 1.01 | 0.02 | 0.47 | 24 |
| 8_E7 | ZAK | 4 | 4 | 2 | 4 |  |  |  | 1.00 | 1.00 | 0.50 | 1.03 |  |  |  | 0.88 | 0.26 | 0.09 | 13 |
| 8_F7 | PIK3C3 | 3 | 1 | 2 | 2 |  |  |  | 0.75 | 0.25 | 0.50 | 0.52 |  |  |  | 0.50 | 0.20 | -1.06 | 8 |
| 8_G7 | HIPK4 | 2 | 3 | 0 | 0 |  |  |  | 0.50 | 0.75 | 0.00 | 0.00 |  |  |  | 0.31 | 0.38 | -1.64 | 0 |
| 8_H7 | MARK4 | 4 | 4 | 4 | 5 |  |  |  | 1.00 | 1.00 | 1.00 | 1.29 |  |  |  | 1.07 | 0.15 | 0.67 | 3 |
| 8_A8 | PIP5K3 | 4 | 4 | 3 | 6 |  |  |  | 1.00 | 1.00 | 0.75 | 1.55 |  |  |  | 1.07 | 0.34 | 0.68 | 5 |
| 8_B8 | BMX | 4 | 4 | 5 | 8 |  |  |  | 1.00 | 1.00 | 1.25 | 2.06 |  |  |  | 1.33 | 0.50 | 1.45 | 11 |
| 8_C8 | EIF2AK4 | 4 | 4 | 5 | 3 |  |  |  | 1.00 | 1.00 | 1.25 | 0.77 |  |  |  | 1.01 | 0.19 | 0.47 | 17 |
| 8_D8 | PNCK | 4 | 4 | 4 | 5 |  |  |  | 1.00 | 1.00 | 1.00 | 1.29 |  |  |  | 1.07 | 0.15 | 0.67 | 6 |
| 8_E8 | PDK4 | 1 | 1 | 2 | 4 |  |  |  | 0.25 | 0.25 | 0.50 | 1.03 |  |  |  | 0.51 | 0.37 | -1.05 | 18 |
| 8_F8 | ROCK2 | 4 | 4 | 3 | 6 |  |  |  | 1.00 | 1.00 | 0.75 | 1.55 |  |  |  | 1.07 | 0.34 | 0.68 | 0 |
| 8_G8 | SIK2 | 2 | 3 | 1 | 0 |  |  |  | 0.50 | 0.75 | 0.25 | 0.00 |  |  |  | 0.38 | 0.32 | -1.45 | 0 |
| 8_H8 | EPHB1 | 2 | 1 | 3 | 2 |  |  |  | 0.50 | 0.25 | 0.75 | 0.52 |  |  |  | 0.50 | 0.20 | -1.06 | 9 |
| 8_A9 | BMPR1A | 2 | 3 | 3 | 4 |  |  |  | 0.50 | 0.75 | 0.75 | 1.03 |  |  |  | 0.76 | 0.22 | -0.29 | 13 |
| 8_B9 | CLK4 | 4 | 4 | 6 | 6 |  |  |  | 1.00 | 1.00 | 1.50 | 1.55 |  |  |  | 1.26 | 0.30 | 1.25 | 5 |
| 8_C9 | NME6 | 3 | 4 | 4 | 6 |  |  |  | 0.75 | 1.00 | 1.00 | 1.55 |  |  |  | 1.07 | 0.34 | 0.68 | 28 |
| 8_D9 | VRK3 | 2 | 3 | 4 | 3 |  |  |  | 0.50 | 0.75 | 1.00 | 0.77 |  |  |  | 0.76 | 0.20 | -0.29 | 7 |
| 8_E9 | CHUK | 3 | 3 | 3 | 3 |  |  |  | 0.75 | 0.75 | 0.75 | 0.77 |  |  |  | 0.76 | 0.01 | -0.29 | 0 |
| 8_F9 | PIK3C2B | 4 | 4 | 3 | 6 |  |  |  | 1.00 | 1.00 | 0.75 | 1.55 |  |  |  | 1.07 | 0.34 | 0.68 | 0 |
| 8_G9 | MET | 4 | 4 | 5 | 4 |  |  |  | 1.00 | 1.00 | 1.25 | 1.03 |  |  |  | 1.07 | 0.12 | 0.66 | 4 |
| 8_H9 | MAGI-3 | 4 | 4 | 3 | 4 |  |  |  | 1.00 | 1.00 | 0.75 | 1.03 |  |  |  | 0.95 | 0.13 | 0.28 | 0 |
| 8_A10 | PRPS1L1 | 3 | 3 | 1 | 0 |  |  |  | 0.75 | 0.75 | 0.25 | 0.00 |  |  |  | 0.44 | 0.38 | -1.26 | 12 |
| 8_B10 | PAK2 | 2 | 2 | 3 | 4 |  |  |  | 0.50 | 0.50 | 0.75 | 1.03 |  |  |  | 0.70 | 0.25 | -0.48 | 20 |
| 8_C10 | DYRK1A | 4 | 4 | 5 | 5 |  |  |  | 1.00 | 1.00 | 1.25 | 1.29 |  |  |  | 1.14 | 0.16 | 0.86 | 13 |
| 8_D10 | PDGFRA | 3 | 3 | 2 | 4 |  |  |  | 0.75 | 0.75 | 0.50 | 1.03 |  |  |  | 0.76 | 0.22 | -0.29 | 8 |
| 8_E10 | TPK1 | 0 | 0 | 1 | 2 |  |  |  | 0.00 | 0.00 | 0.25 | 0.52 |  |  |  | 0.19 | 0.25 | -2.01 | 0 |
| 8_F10 | SGK | 4 | 4 | 4 | 3 |  |  |  | 1.00 | 1.00 | 1.00 | 0.77 |  |  |  | 0.94 | 0.11 | 0.28 | 0 |
| 8_G10 | FYN | 4 | 4 | 4 | 5 |  |  |  | 1.00 | 1.00 | 1.00 | 1.29 |  |  |  | 1.07 | 0.15 | 0.67 | 7 |
| 8_H10 | SSTK | 4 | 4 | 3 | 2 |  |  |  | 1.00 | 1.00 | 0.75 | 0.52 |  |  |  | 0.82 | 0.23 | -0.11 | 8 |
| 8_A11 | IGF2R | 3 | 3 | 3 | 2 |  |  |  | 0.75 | 0.75 | 0.75 | 0.52 |  |  |  | 0.69 | 0.12 | -0.49 | 2 |
| 8_B11 | LOC390226 | 4 | 4 | 3 | 5 |  |  |  | 1.00 | 1.00 | 0.75 | 1.29 |  |  |  | 1.01 | 0.22 | 0.48 | 10 |
| 8_C11 | CAMK4 | 4 | 4 | 5 | 6 |  |  |  | 1.00 | 1.00 | 1.25 | 1.55 |  |  |  | 1.20 | 0.26 | 1.06 | 19 |
| 8_D11 | DDR1 | 4 | 4 | 4 | 5 |  |  |  | 1.00 | 1.00 | 1.00 | 1.29 |  |  |  | 1.07 | 0.15 | 0.67 | 37 |
| 8_E11 | C14ORF20 | 3 | 3 | 4 | 5 |  |  |  | 0.75 | 0.75 | 1.00 | 1.29 |  |  |  | 0.95 | 0.26 | 0.29 | 13 |
| 8_F11 | CLK3 | 4 | 3 | 4 | 4 |  |  |  | 1.00 | 0.75 | 1.00 | 1.03 |  |  |  | 0.95 | 0.13 | 0.28 | 12 |
| 8_G11 | DKFZP434C131 | 3 | 4 | 2 | 3 |  |  |  | 0.75 | 1.00 | 0.50 | 0.77 |  |  |  | 0.76 | 0.20 | -0.29 | 23 |
| 8_H11 | IKBKG | 0 | 1 | 2 | 2 |  |  |  | 0.00 | 0.25 | 0.50 | 0.52 |  |  |  | 0.32 | 0.24 | -1.63 | 18 |
| 9_A1 | NEG | 4 | 4 | 3 | 3 |  |  |  |  |  |  |  |  |  |  |  |  |  |  |
| 9_B1 | NEG | 4 | 4 | 3 | 3 |  |  |  |  |  |  |  |  |  |  |  |  |  |  |
| 9_C1 | NEG | 4 | 4 | 3 | 3 |  |  |  |  |  |  |  |  |  |  |  |  |  |  |
| 9_D1 | NEG | 4 | 4 | 3 | 3 |  |  |  |  |  |  |  |  |  |  |  |  |  |  |
| 9_E1 | NEG | 4 | 4 | 3 | 3 |  |  |  |  |  |  |  |  |  |  |  |  |  |  |
| 9_F1 | NEG | 4 | 4 | 4 | 4 |  |  |  |  |  |  |  |  |  |  |  |  |  |  |
| 9_G1 | NEG | 4 | 4 | 3 | 3 |  |  |  |  |  |  |  |  |  |  |  |  |  |  |
| 9_H1 | NEG | 4 | 4 | 3 | 3 |  |  |  | 4.00 | 4.00 | 3.13 | 3.13 |  |  |  | 3.56 |  |  | 0 |
| 9_A12 | MEK | 2 | 2 | 1 | 2 |  |  |  |  |  |  |  |  |  |  |  |  |  |  |
| 9_B12 | MEK | 3 | 2 | 1 | 2 |  |  |  |  |  |  |  |  |  |  |  |  |  |  |
| 9_C12 | MEK | 1 | 1 | 0 | 0 |  |  |  |  |  |  |  |  |  |  |  |  |  |  |
| 9_D12 | MEK | 3 | 2 | 0 | 0 |  |  |  |  |  |  |  |  |  |  |  |  |  |  |
| 9_E12 | MEK | 0 | 0 | 1 | 1 |  |  |  | 0.45 | 0.35 | 0.19 | 0.32 |  |  |  | 0.33 |  |  | 24 |
| 9_F12 | TOX | 0 | 0 | 0 | 0 |  |  |  |  |  |  |  |  |  |  |  |  |  |  |
| 9_G12 | TOX | 0 | 0 | 0 | 0 |  |  |  |  |  |  |  |  |  |  |  |  |  |  |
| 9_H12 | TOX | 0 | 0 | 0 | 0 |  |  |  | 0.00 | 0.00 | 0.00 | 0.00 |  |  |  | 0.00 |  |  | 100 |
| 9_A2 | N4BP2 | 3 | 4 | 3 | 2 |  |  |  | 0.75 | 1.00 | 0.96 | 0.64 |  |  |  | 0.84 | 0.17 | -0.05 | 15 |
| 9_B2 | PRKCA | 4 | 4 | 4 | 5 |  |  |  | 1.00 | 1.00 | 1.28 | 1.60 |  |  |  | 1.22 | 0.29 | 1.12 | 10 |
| 9_C2 | GUK1 | 4 | 3 | 2 | 3 |  |  |  | 1.00 | 0.75 | 0.64 | 0.96 |  |  |  | 0.84 | 0.17 | -0.05 | 9 |
| 9_D2 | PLK3 | 4 | 4 | 2 | 2 |  |  |  | 1.00 | 1.00 | 0.64 | 0.64 |  |  |  | 0.82 | 0.21 | -0.10 | 0 |
| 9_E2 | AMHR2 | 0 | 0 | 1 | 2 |  |  |  | 0.00 | 0.00 | 0.32 | 0.64 |  |  |  | 0.24 | 0.31 | -1.86 | 11 |
| 9_F2 | PASK | 4 | 4 | 1 | 2 |  |  |  | 1.00 | 1.00 | 0.32 | 0.64 |  |  |  | 0.74 | 0.33 | -0.34 | 1 |
| 9_G2 | EPHA4 | 4 | 4 | 3 | 2 |  |  |  | 1.00 | 1.00 | 0.96 | 0.64 |  |  |  | 0.90 | 0.17 | 0.14 | 0 |
| 9_H2 | NEK7 | 3 | 3 | 2 | 1 |  |  |  | 0.75 | 0.75 | 0.64 | 0.32 |  |  |  | 0.62 | 0.20 | -0.72 | 1 |
| 9_A3 | NEK1 | 4 | 4 | 3 | 4 |  |  |  | 1.00 | 1.00 | 0.96 | 1.28 |  |  |  | 1.06 | 0.15 | 0.63 | 13 |
| 9_B3 | CDKL2 | 2 | 2 | 1 | 1 |  |  |  | 0.50 | 0.50 | 0.32 | 0.32 |  |  |  | 0.41 | 0.10 | -1.34 | 12 |
| 9_C3 | CKM | 2 | 2 | 2 | 2 |  |  |  | 0.50 | 0.50 | 0.64 | 0.64 |  |  |  | 0.57 | 0.08 | -0.86 | 12 |
| 9_D3 | EFNB3 | 3 | 3 | 3 | 2 |  |  |  | 0.75 | 0.75 | 0.96 | 0.64 |  |  |  | 0.78 | 0.13 | -0.24 | 8 |
| 9_E3 | DUSP21 | 4 | 4 | 4 | 4 |  |  |  | 1.00 | 1.00 | 1.28 | 1.28 |  |  |  | 1.14 | 0.16 | 0.87 | 0 |
| 9_F3 | HUS1 | 4 | 4 | 2 | 2 |  |  |  | 1.00 | 1.00 | 0.64 | 0.64 |  |  |  | 0.82 | 0.21 | -0.10 | 2 |
| 9_G3 | DCAMKL1 | 4 | 4 | 4 | 4 |  |  |  | 1.00 | 1.00 | 1.28 | 1.28 |  |  |  | 1.14 | 0.16 | 0.87 | 11 |
| 9_H3 | PRKWNK1 | 4 | 4 | 5 | 4 |  |  |  | 1.00 | 1.00 | 1.60 | 1.28 |  |  |  | 1.22 | 0.29 | 1.12 | 1 |
| 9_A4 | XYLB | 4 | 4 | 2 | 2 |  |  |  | 1.00 | 1.00 | 0.64 | 0.64 |  |  |  | 0.82 | 0.21 | -0.10 | 19 |
| 9_B4 | FES | 4 | 4 | 3 | 3 |  |  |  | 1.00 | 1.00 | 0.96 | 0.96 |  |  |  | 0.98 | 0.02 | 0.39 | 8 |
| 9_C4 | MAP4K3 | 3 | 4 | 3 | 3 |  |  |  | 0.75 | 1.00 | 0.96 | 0.96 |  |  |  | 0.92 | 0.11 | 0.20 | 1 |
| 9_D4 | EPHB3 | 3 | 3 | 2 | 2 |  |  |  | 0.75 | 0.75 | 0.64 | 0.64 |  |  |  | 0.70 | 0.06 | -0.48 | 13 |
| 9_E4 | CDKN2B | 4 | 3 | 4 | 3 |  |  |  | 1.00 | 0.75 | 1.28 | 0.96 |  |  |  | 1.00 | 0.22 | 0.44 | 5 |
| 9_F4 | TLK1 | 4 | 4 | 2 | 3 |  |  |  | 1.00 | 1.00 | 0.64 | 0.96 |  |  |  | 0.90 | 0.17 | 0.14 | 4 |
| 9_G4 | GK2 | 3 | 4 | 2 | 3 |  |  |  | 0.75 | 1.00 | 0.64 | 0.96 |  |  |  | 0.84 | 0.17 | -0.05 | 3 |
| 9_H4 | TSKS | 4 | 3 | 2 | 3 |  |  |  | 1.00 | 0.75 | 0.64 | 0.96 |  |  |  | 0.84 | 0.17 | -0.05 | 4 |
| 9_A5 | RP6-213H19.1 | 4 | 4 | 2 | 2 |  |  |  | 1.00 | 1.00 | 0.64 | 0.64 |  |  |  | 0.82 | 0.21 | -0.10 | 8 |
| 9_B5 | PRKAB1 | 3 | 3 | 3 | 3 |  |  |  | 0.75 | 0.75 | 0.96 | 0.96 |  |  |  | 0.86 | 0.12 | 0.01 | 27 |
| 9_C5 | PRKDC | 3 | 3 | 5 | 4 |  |  |  | 0.75 | 0.75 | 1.60 | 1.28 |  |  |  | 1.10 | 0.42 | 0.74 | 5 |
| 9_D5 | PRKCB1 | 2 | 4 | 2 | 3 |  |  |  | 0.50 | 1.00 | 0.64 | 0.96 |  |  |  | 0.78 | 0.24 | -0.24 | 0 |
| 9_E5 | DLG1 | 4 | 4 | 4 | 4 |  |  |  | 1.00 | 1.00 | 1.28 | 1.28 |  |  |  | 1.14 | 0.16 | 0.87 | 6 |
| 9_F5 | PFKM | 3 | 3 | 3 | 4 |  |  |  | 0.75 | 0.75 | 0.96 | 1.28 |  |  |  | 0.94 | 0.25 | 0.25 | 7 |
| 9_G5 | KUB3 | 2 | 2 | 2 | 3 |  |  |  | 0.50 | 0.50 | 0.64 | 0.96 |  |  |  | 0.65 | 0.22 | -0.62 | 8 |
| 9_H5 | STK19 | 4 | 4 | 4 | 5 |  |  |  | 1.00 | 1.00 | 1.28 | 1.60 |  |  |  | 1.22 | 0.29 | 1.12 | 0 |
| 9_A6 | SMG1 | 3 | 3 | 3 | 3 |  |  |  | 0.75 | 0.75 | 0.96 | 0.96 |  |  |  | 0.86 | 0.12 | 0.01 | 25 |
| 9_B6 | HAK | 4 | 4 | 2 | 2 |  |  |  | 1.00 | 1.00 | 0.64 | 0.64 |  |  |  | 0.82 | 0.21 | -0.10 | 3 |
| 9_C6 | PRPF4B | 3 | 3 | 4 | 3 |  |  |  | 0.75 | 0.75 | 1.28 | 0.96 |  |  |  | 0.94 | 0.25 | 0.25 | 0 |
| 9_D6 | DGKD | 4 | 4 | 4 | 5 |  |  |  | 1.00 | 1.00 | 1.28 | 1.60 |  |  |  | 1.22 | 0.29 | 1.12 | 3 |
| 9_E6 | C9ORF96 | 0 | 1 | 0 | 1 |  |  |  | 0.00 | 0.25 | 0.00 | 0.32 |  |  |  | 0.14 | 0.17 | -2.16 | 7 |
| 9_F6 | PRKAA2 | 0 | 0 | 2 | 1 |  |  |  | 0.00 | 0.00 | 0.64 | 0.32 |  |  |  | 0.24 | 0.31 | -1.86 | 5 |
| 9_G6 | TRPM7 | 3 | 3 | 3 | 2 |  |  |  | 0.75 | 0.75 | 0.96 | 0.64 |  |  |  | 0.78 | 0.13 | -0.24 | 9 |
| 9_H6 | MAPK12 | 3 | 2 | 2 | 2 |  |  |  | 0.75 | 0.50 | 0.64 | 0.64 |  |  |  | 0.63 | 0.10 | -0.67 | 1 |
| 9_A7 | IHPK2 | 4 | 4 | 3 | 2 |  |  |  | 1.00 | 1.00 | 0.96 | 0.64 |  |  |  | 0.90 | 0.17 | 0.14 | 17 |
| 9_B7 | CDK2 | 3 | 3 | 0 | 1 |  |  |  | 0.75 | 0.75 | 0.00 | 0.32 |  |  |  | 0.46 | 0.36 | -1.21 | 11 |
| 9_C7 | RIOK3 | 4 | 4 | 1 | 3 |  |  |  | 1.00 | 1.00 | 0.32 | 0.96 |  |  |  | 0.82 | 0.33 | -0.10 | 0 |
| 9_D7 | EPHB4 | 4 | 4 | 1 | 3 |  |  |  | 1.00 | 1.00 | 0.32 | 0.96 |  |  |  | 0.82 | 0.33 | -0.10 | 9 |
| 9_E7 | MAP2K1 | 0 | 0 | 2 | 1 |  |  |  | 0.00 | 0.00 | 0.64 | 0.32 |  |  |  | 0.24 | 0.31 | -1.86 | 13 |
| 9_F7 | ITPKB | 0 | 0 | 0 | 1 |  |  |  | 0.00 | 0.00 | 0.00 | 0.32 |  |  |  | 0.08 | 0.16 | -2.35 | 14 |
| 9_G7 | MYLK | 4 | 3 | 1 | 3 |  |  |  | 1.00 | 0.75 | 0.32 | 0.96 |  |  |  | 0.76 | 0.31 | -0.29 | 17 |
| 9_H7 | GALK1 | 4 | 4 | 4 | 3 |  |  |  | 1.00 | 1.00 | 1.28 | 0.96 |  |  |  | 1.06 | 0.15 | 0.63 | 5 |
| 9_A8 | ILK | 4 | 4 | 5 | 4 |  |  |  | 1.00 | 1.00 | 1.60 | 1.28 |  |  |  | 1.22 | 0.29 | 1.12 | 2 |
| 9_B8 | MAP2K2 | 3 | 3 | 2 | 3 |  |  |  | 0.75 | 0.75 | 0.64 | 0.96 |  |  |  | 0.78 | 0.13 | -0.24 | 6 |
| 9_C8 | KIAA1765 | 4 | 4 | 2 | 3 |  |  |  | 1.00 | 1.00 | 0.64 | 0.96 |  |  |  | 0.90 | 0.17 | 0.14 | 11 |
| 9_D8 | MGC45428 | 2 | 2 | 0 | 1 |  |  |  | 0.50 | 0.50 | 0.00 | 0.32 |  |  |  | 0.33 | 0.24 | -1.59 | 0 |
| 9_E8 | GALK2 | 3 | 3 | 2 | 2 |  |  |  | 0.75 | 0.75 | 0.64 | 0.64 |  |  |  | 0.70 | 0.06 | -0.48 | 23 |
| 9_F8 | MPP3 | 3 | 3 | 2 | 3 |  |  |  | 0.75 | 0.75 | 0.64 | 0.96 |  |  |  | 0.78 | 0.13 | -0.24 | 28 |
| 9_G8 | GUCY2F | 4 | 2 | 2 | 1 |  |  |  | 1.00 | 0.50 | 0.64 | 0.32 |  |  |  | 0.62 | 0.29 | -0.72 | 16 |
| 9_H8 | IPMK | 3 | 3 | 3 | 4 |  |  |  | 0.75 | 0.75 | 0.96 | 1.28 |  |  |  | 0.94 | 0.25 | 0.25 | 9 |
| 9_A9 | PSKH2 | 4 | 4 | 2 | 2 |  |  |  | 1.00 | 1.00 | 0.64 | 0.64 |  |  |  | 0.82 | 0.21 | -0.10 | 3 |
| 9_B9 | MAPK10 | 2 | 2 | 2 | 2 |  |  |  | 0.50 | 0.50 | 0.64 | 0.64 |  |  |  | 0.57 | 0.08 | -0.86 | 0 |
| 9_C9 | EXOSC10 | 1 | 0 | 1 | 0 |  |  |  | 0.25 | 0.00 | 0.32 | 0.00 |  |  |  | 0.14 | 0.17 | -2.16 | 10 |
| 9_D9 | CHKB | 4 | 3 | 2 | 2 |  |  |  | 1.00 | 0.75 | 0.64 | 0.64 |  |  |  | 0.76 | 0.17 | -0.29 | 0 |
| 9_E9 | NTRK1 | 3 | 3 | 1 | 1 |  |  |  | 0.75 | 0.75 | 0.32 | 0.32 |  |  |  | 0.54 | 0.25 | -0.96 | 6 |
| 9_F9 | CDK3 | 0 | 1 | 0 | 0 |  |  |  | 0.00 | 0.25 | 0.00 | 0.00 |  |  |  | 0.06 | 0.13 | -2.40 | 16 |
| 9_G9 | GSK3B | 4 | 4 | 3 | 3 |  |  |  | 1.00 | 1.00 | 0.96 | 0.96 |  |  |  | 0.98 | 0.02 | 0.39 | 14 |
| 9_H9 | PANK4 | 0 | 0 | 1 | 0 |  |  |  | 0.00 | 0.00 | 0.32 | 0.00 |  |  |  | 0.08 | 0.16 | -2.35 | 11 |
| 9_A10 | NRBP | 4 | 4 | 4 | 4 |  |  |  | 1.00 | 1.00 | 1.28 | 1.28 |  |  |  | 1.14 | 0.16 | 0.87 | 7 |
| 9_B10 | CALM2 | 0 | 0 | 0 | 0 |  |  |  | 0.00 | 0.00 | 0.00 | 0.00 |  |  |  | 0.00 | 0.00 | -2.59 | 1 |
| 9_C10 | PGK1 | 2 | 2 | 2 | 2 |  |  |  | 0.50 | 0.50 | 0.64 | 0.64 |  |  |  | 0.57 | 0.08 | -0.86 | 13 |
| 9_D10 | PRKAR2B | 3 | 3 | 2 | 1 |  |  |  | 0.75 | 0.75 | 0.64 | 0.32 |  |  |  | 0.62 | 0.20 | -0.72 | 6 |
| 9_E10 | NME4 | 2 | 2 | 1 | 2 |  |  |  | 0.50 | 0.50 | 0.32 | 0.64 |  |  |  | 0.49 | 0.13 | -1.10 | 14 |
| 9_F10 | PDPK1 | 4 | 4 | 2 | 3 |  |  |  | 1.00 | 1.00 | 0.64 | 0.96 |  |  |  | 0.90 | 0.17 | 0.14 | 17 |
| 9_G10 | MST1R | 3 | 4 | 3 | 3 |  |  |  | 0.75 | 1.00 | 0.96 | 0.96 |  |  |  | 0.92 | 0.11 | 0.20 | 9 |
| 9_H10 | PRKACG | 4 | 4 | 2 | 3 |  |  |  | 1.00 | 1.00 | 0.64 | 0.96 |  |  |  | 0.90 | 0.17 | 0.14 | 0 |
| 9_A11 | ULK1 | 4 | 2 | 1 | 2 |  |  |  | 1.00 | 0.50 | 0.32 | 0.64 |  |  |  | 0.62 | 0.29 | -0.72 | 10 |
| 9_B11 | KHK | 2 | 2 | 1 | 2 |  |  |  | 0.50 | 0.50 | 0.32 | 0.64 |  |  |  | 0.49 | 0.13 | -1.10 | 0 |
| 9_C11 | AK3L1 | 3 | 2 | 2 | 3 |  |  |  | 0.75 | 0.50 | 0.64 | 0.96 |  |  |  | 0.71 | 0.19 | -0.43 | 49 |
| 9_D11 | PCK2 | 2 | 2 | 0 | 0 |  |  |  | 0.50 | 0.50 | 0.00 | 0.00 |  |  |  | 0.25 | 0.29 | -1.83 | 20 |
| 9_E11 | BRAF | 4 | 4 | 1 | 0 |  |  |  | 1.00 | 1.00 | 0.32 | 0.00 |  |  |  | 0.58 | 0.50 | -0.83 | 18 |
| 9_F11 | C10ORF89 | 3 | 4 | 1 | 2 |  |  |  | 0.75 | 1.00 | 0.32 | 0.64 |  |  |  | 0.68 | 0.28 | -0.53 | 11 |
| 9_G11 | AK2 | 3 | 2 | 2 | 3 |  |  |  | 0.75 | 0.50 | 0.64 | 0.96 |  |  |  | 0.71 | 0.19 | -0.43 | 17 |
| 9_H11 | FLT1 | 4 | 4 | 4 | 4 |  |  |  | 1.00 | 1.00 | 1.28 | 1.28 |  |  |  | 1.14 | 0.16 | 0.87 | 3 |
