# Supplementary material for: Identification of Host Kinase Genes Required for Influenza Virus Replication and the Regulatory Role of MicroRNAs
Source: PLoS One. 2013 Jun 21;8(6):e66796. doi: 10.1371/journal.pone.0066796 (PMC3689682; doi:10.1371/journal.pone.0066796)
Supplement: Table S2 — Sequences of siRNAs used in the study. Table shows accession numbers, gene symbol, gene id and siRNA sequences used for validation of hits from primary screen. (DOCX) [file pone.0066796.s008.docx]

| **Supplemental Table S2. Dharmacon siRNA sequences.** | | | |
| --- | --- | --- | --- |
| Gene Accession | Gene Symbol | Gene ID | VALIDATION SCREEN |
|  |  |  | Novel siRNA target |
| NM_014911 | AAK1 | 22848 |  |
| NM_001080395 | AATK | 9625 |  |
| NM_005157 | ABL1 | 25 |  |
| NM_005158 | ABL2 | 25 |  |
| NM_001105 | ACVR1 | 27 |  |
| NM_004302 | ACVR1B | 90 |  |
| NM_001111033 | ACVR1C | 91 |  |
| NM_001616 | ACVR2 | 130399 |  |
| NM_001106 | ACVR2B | 92 |  |
| NM_001077401 | ACVRL1 | 93 |  |
| NM_020421 | ADCK1 | 94 |  |
| NM_052853 | ADCK2 | 57143 |  |
| NM_024876 | ADCK4 | 57143 |  |
| NM_174922 | ADCK5 | 90956 |  |
| NM_001123 | ADK | 79934 | GAAUCUAUCUGCACCGUU |
| NM_031284 | ADP-GK | 203054 |  |
| NM_001619 | ADRBK1 | 132 |  |
| NM_005160 | ADRBK2 | 156 |  |
| NM_012301 | AIP1 | 157 |  |
| NM_000476 | AK1 | 9863 |  |
| NM_001625 | AK2 | 9863 |  |
| NM_013410 | AK3 | 204 |  |
| NM_016282 | AK3L1 | 205 |  |
| NM_012093 | AK5 | 50808 |  |
| NM_152327 | AK7 | 26289 |  |
| NM_001014431 | AKT1 | 208 |  |
| NM_001626 | AKT2 | 208 |  |
| NM_005465 | AKT3 | 10000 |  |
| NM_004304 | ALK | 238 |  |
| NM_018571 | ALS2CR2 | 55437 |  |
| NM_139158 | ALS2CR7 | 269 |  |
| NM_020547 | AMHR2 | 54101 |  |
| NM_178510 | ANKK1 | 54101 |  |
| NM_020639 | ANKRD3 | 369 |  |
| NM_001654 | ARAF1 | 9891 |  |
| NM_014840 | ARK5 | 10926 |  |
| NM_006716 | ASK | 472 |  |
| NM_138292 | ATM | 545 |  |
| NM_001184 | ATR | 6790 |  |
| NM_198437 | AURKA | 9212 |  |
| NM_004217 | AURKB | 558 |  |
| NM_001015879 | AURKC | 558 |  |
| NM_001699 | AXL | 9223 |  |
| NM_004742 | BAIAP1 | 10295 |  |
| NM_005881 | BCKDK | 10295 |  |
| NM_021574 | BCR | 613 |  |
| NM_001715 | BLK | 640 |  |
| NM_017593 | BMP2K | 640 |  |
| NM_004329 | BMPR1A | 657 |  |
| NM_001203 | BMPR1B | 658 |  |
| NM_001204 | BMPR2 | 659 |  |
| NM_001721 | BMX | 660 |  |
| NM_004333 | BRAF | 673 |  |
| NM_001113182 | BRD2 | 6046 |  |
| NM_007371 | BRD3 | 8019 |  |
| NM_014299 | BRD4 | 23476 |  |
| NM_001726 | BRDT | 676 |  |
| NM_000061 | BTK | 695 |  |
| NM_004336 | BUB1 | 695 |  |
| NM_001211 | BUB1B | 699 |  |
| NM_153336 | C10ORF89 | 699 |  |
| NM_174944 | C14ORF20 | 701 |  |
| NM_022458 | C7ORF2 | 118672 |  |
| NM_022755 | C9ORF12 | 283629 |  |
| NM_153710 | C9ORF96 | 64327 | UAAAGGACGUGGUGCACAU |
| NM_006888 | CALM1 | 64768 |  |
| NM_001743 | CALM2 | 169436 | GUUAACAGAUGAAGAAGUU |
| NM_005184 | CALM3 | 805 |  |
| NM_003656 | CAMK1 | 808 |  |
| NM_020397 | CAMK1D | 8536 |  |
| NM_020439 | CAMK1G | 57118 |  |
| NM_015981 | CAMK2A | 57172 |  |
| NM_172084 | CAMK2B | 57172 |  |
| NM_001221 | CAMK2D | 815 |  |
| NM_001222 | CAMK2G | 816 |  |
| NM_001744 | CAMK4 | 817 |  |
| NM_018584 | CAMKIINALPHA | 814 |  |
| NM_172207 | CAMKK1 | 84254 |  |
| NM_172215 | CAMKK2 | 23729 |  |
| NM_013276 | CARKL | 8573 |  |
| NM_003688 | CASK | 23552 |  |
| NM_012119 | CCRK | 23552 |  |
| NM_030911 | CDADC1 | 81602 |  |
| NM_001786 | CDC2 | 81602 |  |
| NM_033487 | CDC2L1 | 983 |  |
| NM_033529 | CDC2L2 | 984 |  |
| NM_031267 | CDC2L5 | 728642 | GAAGAAAGUCGGCCGUAUA |
| NM_014826 | CDC42BPA | 8621 |  |
| NM_006035 | CDC42BPB | 8476 |  |
| NM_003503 | CDC7 | 9578 |  |
| NM_052987 | CDK10 | 9578 |  |
| NM_015076 | CDK11 | 8558 |  |
| NM_001798 | CDK2 | 23097 |  |
| NM_001258 | CDK3 | 1017 | GAAGAUCAGACUGGAUUUG |
| NM_000075 | CDK4 | 1018 |  |
| NM_004935 | CDK5 | 1019 |  |
| NM_003885 | CDK5R1 | 1019 |  |
| NM_003936 | CDK5R2 | 1020 |  |
| NM_001259 | CDK6 | 8851 |  |
| NM_001799 | CDK7 | 8941 |  |
| NM_001260 | CDK8 | 1021 |  |
| NM_001261 | CDK9 | 1022 |  |
| NM_004196 | CDKL1 | 1024 |  |
| NM_003948 | CDKL2 | 1024 |  |
| NM_016508 | CDKL3 | 1025 |  |
| NM_001009565 | CDKL4 | 8814 |  |
| NM_001037343 | CDKL5 | 8999 |  |
| NM_000389 | CDKN1A | 51265 |  |
| NM_004064 | CDKN1B | 344387 |  |
| NM_000076 | CDKN1C | 6792 |  |
| NM_004936 | CDKN2B | 1026 |  |
| NM_001262 | CDKN2C | 1027 |  |
| NM_079421 | CDKN2D | 1028 |  |
| NM_022766 | CERK | 1030 |  |
| NM_001274 | CHEK1 | 1031 |  |
| NM_145862 | CHEK2 | 1032 |  |
| NM_212469 | CHKA | 64781 |  |
| NM_005198 | CHKB | 1111 |  |
| NM_001278 | CHUK | 11200 |  |
| NM_006383 | CIB2 | 1119 |  |
| NM_007174 | CIT | 1120 |  |
| NM_001823 | CKB | 1147 |  |
| NM_001824 | CKM | 10518 |  |
| NM_020990 | CKMT1B | 11113 |  |
| NM_001099735 | CKMT2 | 1152 |  |
| NM_001826 | CKS1B | 1158 |  |
| NM_001827 | CKS2 | 1160 |  |
| NM_004071 | CLK1 | 1164 |  |
| NM_003993 | CLK2 | 1196 |  |
| NM_001292 | CLK3 | 1198 |  |
| NM_020666 | CLK4 | 57396 |  |
| NM_025233 | COASY | 80347 |  |
| NM_005713 | COL4A3BP | 10087 |  |
| NM_012071 | COMMD3 | 23412 |  |
| NM_003909 | CPNE3 | 8895 |  |
| NM_016441 | CRIM1 | 51232 |  |
| NM_015083 | CRK7 | 51755 |  |
| NM_005207 | CRKL | 1399 |  |
| NM_005211 | CSF1R | 1436 |  |
| NM_004383 | CSK | 1436 |  |
| NM_001892 | CSNK1A1 | 1445 |  |
| NM_145203 | CSNK1A1L | 1452 |  |
| NM_001893 | CSNK1D | 122011 |  |
| NM_001894 | CSNK1E | 1453 |  |
| NM_022048 | CSNK1G1 | 1454 |  |
| NM_001319 | CSNK1G2 | 1454 |  |
| NM_001044722 | CSNK1G3 | 1455 |  |
| NM_177560 | CSNK2A1 | 1456 |  |
| NM_001896 | CSNK2A2 | 1457 |  |
| NM_001320 | CSNK2B | 1459 |  |
| NM_004938 | DAPK1 | 1460 |  |
| NM_014326 | DAPK2 | 1612 |  |
| NM_001348 | DAPK3 | 1612 |  |
| NM_004734 | DCAMKL1 | 23604 |  |
| NM_000788 | DCK | 1613 |  |
| NM_013994 | DDR1 | 9201 |  |
| NM_006182 | DDR2 | 1633 |  |
| NM_201554 | DGKA | 780 |  |
| NM_004080 | DGKB | 4921 |  |
| NM_003648 | DGKD | 1606 |  |
| NM_001080745 | DGKG | 8527 |  |
| NM_152910 | DGKH | 1608 |  |
| NM_004717 | DGKI | 1608 |  |
| NM_001013742 | DGKK | 160851 |  |
| NM_001347 | DGKQ | 9162 |  |
| NM_080918 | DGUOK | 139189 |  |
| NM_001099436 | DKFZP434C131 | 139189 |  |
| NM_001080826 | DKFZP761P0423 | 1609 |  |
| NM_001098424 | DLG1 | 1609 |  |
| NM_001364 | DLG2 | 1716 |  |
| NM_020730 | DLG3 | 25989 |  |
| NM_001365 | DLG4 | 157285 |  |
| NM_001081562 | DMPK | 1739 |  |
| NM_012145 | DTYMK | 1740 |  |
| NM_022076 | DUSP21 | 1741 |  |
| NM_199462 | DUSTYPK | 1742 |  |
| NM_130438 | DYRK1A | 1742 |  |
| NM_004714 | DYRK1B | 1760 |  |
| NM_003583 | DYRK2 | 1841 |  |
| NM_003582 | DYRK3 | 63904 | GGAAUAGCCAAUAAGCUUA |
| NM_003845 | DYRK4 | 25778 |  |
| NM_013302 | EEF2K | 1859 |  |
| NM_004952 | EFNA3 | 9149 |  |
| NM_005227 | EFNA4 | 8445 |  |
| NM_001962 | EFNA5 | 8445 |  |
| NM_001406 | EFNB3 | 8444 |  |
| NM_201283 | EGFR | 1944 |  |
| NM_004836 | EIF2AK3 | 1944 |  |
| NM_001013703 | EIF2AK4 | 1945 |  |
| NM_005232 | EPHA1 | 1945 |  |
| NM_173641 | EPHA10 | 1946 |  |
| NM_004431 | EPHA2 | 1949 |  |
| NM_182644 | EPHA3 | 1956 |  |
| NM_004438 | EPHA4 | 9451 |  |
| NM_182472 | EPHA5 | 440275 |  |
| NM_173655 | EPHA6 | 2041 | CGGAAUAUACUGGUCAAUA |
| NM_004440 | EPHA7 | 1969 |  |
| NM_001006943 | EPHA8 | 2042 |  |
| NM_004441 | EPHB1 | 2043 |  |
| NM_017449 | EPHB2 | 285220 |  |
| NM_004443 | EPHB3 | 2045 |  |
| NM_004444 | EPHB4 | 2046 |  |
| NM_004445 | EPHB6 | 2047 |  |
| NM_004448 | ERBB2 | 2048 |  |
| NM_001005915 | ERBB3 | 2049 |  |
| NM_001042599 | ERBB4 | 2050 | GCAAGAAUUGACUCGAAUA |
| NM_139021 | ERK8 | 2051 |  |
| NM_001433 | ERN1 | 2064 |  |
| NM_033266 | ERN2 | 2064 |  |
| NM_001039481 | ETNK1 | 2065 |  |
| NM_002685 | EXOSC10 | 2066 | ACGAAAAGCUCUUGAAUUG |
| NM_006712 | FASTK | 2081 |  |
| NM_005246 | FER | 10595 |  |
| NM_002005 | FES | 5394 |  |
| NM_023108 | FGFR1 | 10922 |  |
| NM_000141 | FGFR2 | 10922 |  |
| NM_000142 | FGFR3 | 2241 |  |
| NM_002011 | FGFR4 | 2242 |  |
| NM_021923 | FGFRL1 | 2260 |  |
| NM_001042729 | FGR | 2260 |  |
| NM_018208 | FLJ10761 | 2263 |  |
| NM_023018 | FLJ13052 | 2264 |  |
| NM_001018046 | FLJ23074 | 53834 |  |
| NM_032237 | FLJ23356 | 55224 |  |
| NM_144610 | FLJ25006 | 55224 |  |
| NM_152534 | FLJ32685 | 65220 |  |
| NM_152649 | FLJ34389 | 80122 |  |
| NM_002019 | FLT1 | 84197 |  |
| NM_004119 | FLT3 | 84197 |  |
| NM_002020 | FLT4 | 84197 |  |
| NM_022158 | FN3K | 124923 |  |
| NM_024619 | FN3KRP | 197259 |  |
| NM_004958 | FRAP1 | 2321 |  |
| NM_000144 | FRDA | 2322 |  |
| NM_002031 | FRK | 2322 |  |
| NM_145059 | FUK | 2324 |  |
| NM_153048 | FYN | 64122 |  |
| NM_005255 | GAK | 79672 |  |
| NM_000154 | GALK1 | 79672 |  |
| NM_002044 | GALK2 | 2395 |  |
| NM_000162 | GCK | 2444 |  |
| NM_000167 | GK | 197258 |  |
| NM_033214 | GK2 | 2534 |  |
| NM_005476 | GNE | 2580 |  |
| NM_005113 | GOLGA5 | 2584 |  |
| NM_002929 | GRK1 | 2585 |  |
| NM_001004057 | GRK4 | 2645 |  |
| NM_005308 | GRK5 | 2710 |  |
| NM_001004105 | GRK6 | 2712 |  |
| NM_139209 | GRK7 | 10020 |  |
| NM_031965 | GSG2 | 6011 |  |
| NM_019884 | GSK3A | 2868 |  |
| NM_002093 | GSK3B | 2869 |  |
| NM_005316 | GTF2H1 | 2870 |  |
| NM_004963 | GUCY2C | 131890 |  |
| NM_000180 | GUCY2D | 83903 |  |
| NM_001522 | GUCY2F | 2931 |  |
| NM_000858 | GUK1 | 2931 |  |
| NM_052947 | HAK | 2932 |  |
| NM_002110 | HCK | 2965 |  |
| NM_152696 | HIPK1 | 2984 |  |
| NM_022740 | HIPK2 | 2986 |  |
| NM_001048200 | HIPK3 | 2987 |  |
| NM_144685 | HIPK4 | 115701 |  |
| NM_000188 | HK1 | 204851 |  |
| NM_000189 | HK2 | 28996 | GAGUGGAGAUGCACAACAA |
| NM_002115 | HK3 | 10114 |  |
| NM_014413 | HRI | 10114 |  |
| NM_017525 | HSMDPKIN | 147746 |  |
| NM_014365 | HSPB8 | 3098 |  |
| NM_014586 | HUNK | 3099 |  |
| NM_004507 | HUS1 | 3101 |  |
| NM_014920 | ICK | 27102 |  |
| NM_000875 | IGF1R | 55561 |  |
| NM_000876 | IGF2R | 55561 |  |
| NM_001006115 | IHPK1 | 26353 |  |
| NM_016291 | IHPK2 | 26353 |  |
| NM_054111 | IHPK3 | 30811 |  |
| NM_003640 | IKBKAP | 3364 |  |
| NM_001556 | IKBKB | 22858 |  |
| NM_014002 | IKBKE | 3480 |  |
| NM_001099856 | IKBKG | 3480 |  |
| NM_001014794 | ILK | 3482 |  |
| AJ277481 | ILK-2 | 9807 |  |
| NM_001079817 | INSR | 51447 |  |
| NM_014215 | INSRR | 117283 |  |
| NM_152230 | IPMK | 8518 |  |
| NM_001569 | IRAK1 | 3551 |  |
| NM_001570 | IRAK2 | 9641 |  |
| NM_007199 | IRAK3 | 8517 |  |
| NM_016123 | IRAK4 | 3611 |  |
| NM_005546 | ITK | 55522 |  |
| NM_014216 | ITPK1 | 3643 |  |
| NM_002220 | ITPKA | 3645 |  |
| NM_002221 | ITPKB | 253430 | GAUGAUCGACUUUGGGAAA |
| NM_025194 | ITPKC | 3656 |  |
| NM_002227 | JAK1 | 11213 |  |
| NM_004972 | JAK2 | 3702 |  |
| NM_000215 | JAK3 | 3705 |  |
| NM_016281 | JIK | 3706 |  |
| NM_003947 | KALRN | 3706 |  |
| NM_172056 | KCNH2 | 3707 |  |
| NM_144633 | KCNH8 | 3716 |  |
| NM_002253 | KDR | 3717 |  |
| NM_006488 | KHK | 3718 |  |
| NM_025164 | KIAA0999 | 3718 |  |
| NM_020791 | KIAA1361 | 51347 |  |
| XM_290923 | KIAA1639 | 8997 |  |
| NM_033403 | KIAA1765 | 3757 |  |
| NM_032435 | KIAA1804 | 131096 |  |
| NM_032430 | KIAA1811 | 3795 |  |
| NM_024776 | KIAA2002 | 23387 |  |
| NM_001093772 | KIT | 23387 |  |
| NM_014238 | KSR | 85443 |  |
| NM_173598 | KSR2 | 84446 |  |
| NM_033276 | KUB3 | 79834 |  |
| NM_001102406 | LAK | 8844 |  |
| NM_004690 | LATS1 | 283455 |  |
| NM_014572 | LATS2 | 91419 |  |
| NM_001042771 | LCK | 80216 |  |
| NM_002314 | LIMK1 | 26524 |  |
| NM_001031801 | LIMK2 | 26524 |  |
| NM_014916 | LMTK2 | 3932 |  |
| NM_001080434 | LMTK3 | 3984 |  |
| NM_001012418 | LOC340156 | 3985 |  |
| XM_001134406 | LOC390226 | 22853 |  |
| NM_138370 | LOC91461 | 114783 |  |
| NM_024652 | LRRK1 | 340156 |  |
| NM_198578 | LRRK2 | 390226 |  |
| NM_206961 | LTK | 91461 |  |
| NM_001003786 | LYK5 | 79705 |  |
| NM_001111097 | LYN | 120892 |  |
| NM_152900 | MAGI-3 | 4058 |  |
| NM_005906 | MAK | 92335 |  |
| NM_002755 | MAP2K1 | 4067 |  |
| NM_030662 | MAP2K2 | 260425 |  |
| NM_002756 | MAP2K3 | 4117 |  |
| NM_003010 | MAP2K4 | 5604 |  |
| NM_002757 | MAP2K5 | 5605 |  |
| NM_002758 | MAP2K6 | 5606 |  |
| NM_145185 | MAP2K7 | 6416 |  |
| NM_005921 | MAP3K1 | 5607 | GAUUAGAUGUCAAUACAGA |
| NM_002446 | MAP3K10 | 5608 |  |
| NM_002419 | MAP3K11 | 5609 |  |
| NM_006301 | MAP3K12 | 4214 |  |
| NM_004721 | MAP3K13 | 4294 |  |
| NM_003954 | MAP3K14 | 4296 |  |
| NM_001001671 | MAP3K15 | 7786 |  |
| NM_006609 | MAP3K2 | 7786 |  |
| NM_002401 | MAP3K3 | 9175 |  |
| NM_006724 | MAP3K4 | 9020 |  |
| NM_005923 | MAP3K5 | 389840 |  |
| NM_004672 | MAP3K6 | 389840 |  |
| NM_145333 | MAP3K7 | 10746 |  |
| NM_153497 | MAP3K7IP1 | 10746 |  |
| NM_005204 | MAP3K8 | 4215 |  |
| NM_033141 | MAP3K9 | 4216 |  |
| NM_007181 | MAP4K1 | 9064 |  |
| NM_004579 | MAP4K2 | 6885 |  |
| NM_003618 | MAP4K3 | 10454 |  |
| NM_004834 | MAP4K4 | 1326 |  |
| NM_006575 | MAP4K5 | 1326 |  |
| NM_138957 | MAPK1 | 11184 |  |
| NM_002753 | MAPK10 | 5871 |  |
| NM_002751 | MAPK11 | 8491 |  |
| NM_002969 | MAPK12 | 9448 |  |
| NM_002754 | MAPK13 | 11183 |  |
| NM_139013 | MAPK14 | 5594 |  |
| NM_001109891 | MAPK3 | 5602 |  |
| NM_002747 | MAPK4 | 5600 |  |
| NM_002748 | MAPK6 | 6300 |  |
| NM_002749 | MAPK7 | 5603 |  |
| NM_139047 | MAPK8 | 1432 |  |
| NM_002752 | MAPK9 | 5595 |  |
| NM_004759 | MAPKAPK2 | 5595 |  |
| NM_004635 | MAPKAPK3 | 5596 |  |
| NM_003668 | MAPKAPK5 | 5597 |  |
| NM_018650 | MARK1 | 5598 |  |
| NM_001039468 | MARK2 | 5599 |  |
| NM_002376 | MARK3 | 5601 |  |
| NM_031417 | MARK4 | 5601 |  |
| NM_015112 | MAST2 | 9261 |  |
| NM_015016 | MAST3 | 7867 |  |
| NM_198828 | MAST4 | 8550 |  |
| NM_032844 | MASTL | 4139 |  |
| NM_139354 | MATK | 2011 |  |
| NM_014791 | MELK | 4140 |  |
| NM_006343 | MERTK | 57787 |  |
| NM_000245 | MET | 23139 |  |
| NM_033115 | MGC16169 | 23031 |  |
| NM_153361 | MGC42105 | 23031 |  |
| NM_001040261 | MGC45428 | 375449 |  |
| NM_032017 | MGC4796 | 84930 |  |
| NM_024046 | MGC8407 | 9833 |  |
| NM_020778 | MIDORI | 10461 |  |
| NM_015716 | MINK | 4233 |  |
| NM_198973 | MKNK1 | 93627 |  |
| NM_017572 | MKNK2 | 167359 |  |
| NM_182493 | MLCK | 166614 |  |
| NM_005372 | MOS | 83931 |  |
| NM_002436 | MPP1 | 83931 |  |
| NM_005374 | MPP2 | 79012 |  |
| NM_001932 | MPP3 | 57538 |  |
| NM_002447 | MST1R | 50488 |  |
| NM_018238 | MULK | 8569 |  |
| NM_005592 | MUSK | 8569 |  |
| NM_000431 | MVK | 4342 |  |
| NM_053031 | MYLK | 4354 |  |
| NM_033118 | MYLK2 | 4354 |  |
| NM_017433 | MYO3A | 4355 |  |
| NM_001083615 | MYO3B | 4355 |  |
| NM_018177 | N4BP2 | 4356 |  |
| NM_017567 | NAGK | 4486 |  |
| NM_012224 | NEK1 | 55750 |  |
| NM_145910 | NEK11 | 4593 |  |
| NM_002497 | NEK2 | 4598 |  |
| NM_152720 | NEK3 | 4638 | CCUGAAGACAUGAUACUUA |
| NM_003157 | NEK4 | 53904 |  |
| NM_199289 | NEK5 | 140469 |  |
| NM_014397 | NEK6 | 55728 |  |
| NM_133494 | NEK7 | 4750 |  |
| NM_178170 | NEK8 | 4751 | AGACAAAGCCCUUAUGAUC |
| NM_033116 | NEK9 | 4752 |  |
| NM_016231 | NLK | 341676 |  |
| NM_000269 | NME1 | 10783 |  |
| NM_001018139 | NME2 | 10783 |  |
| NM_002513 | NME3 | 140609 |  |
| NM_005009 | NME4 | 284086 |  |
| NM_003551 | NME5 | 91754 |  |
| NM_005793 | NME6 | 91754 |  |
| NM_013330 | NME7 | 51701 |  |
| NM_003995 | NPR2 | 4830 | UGAGAGAUGUUCAGUUCAA |
| NM_013392 | NRBP | 4831 |  |
| NM_178564 | NRBP2 | 4832 |  |
| NM_198465 | NRK | 4833 |  |
| NM_001007792 | NTRK1 | 10201 |  |
| NM_001018064 | NTRK2 | 29922 |  |
| NM_002530 | NTRK3 | 29959 |  |
| NM_022731 | NUCKS | 340371 |  |
| NM_012346 | NUP62 | 203447 |  |
| NM_033516 | NYD-SP25 | 4914 |  |
| NM_005109 | OSR1 | 4915 |  |
| NM_014308 | P101-PI3K | 4916 |  |
| NM_020423 | PACE-1 | 64710 |  |
| NM_020804 | PACSIN1 | 23636 |  |
| NM_002576 | PAK1 | 89882 |  |
| NM_002577 | PAK2 | 89882 |  |
| NM_002578 | PAK3 | 9943 |  |
| NM_001014834 | PAK4 | 23533 |  |
| NM_020168 | PAK6 | 23533 |  |
| NM_020341 | PAK7 | 57147 |  |
| NM_138316 | PANK1 | 29993 |  |
| NM_024960 | PANK2 | 5058 |  |
| NM_024594 | PANK3 | 5062 |  |
| NM_018216 | PANK4 | 5063 | GCGCUUCGCCAUCGACAUA |
| NM_005443 | PAPSS1 | 10298 |  |
| NM_004670 | PAPSS2 | 56924 |  |
| NM_015148 | PASK | 56924 |  |
| NM_002591 | PCK1 | 57144 |  |
| NM_004563 | PCK2 | 53354 |  |
| NM_006201 | PCTK1 | 80025 |  |
| NM_002595 | PCTK2 | 79646 |  |
| NM_002596 | PCTK3 | 55229 |  |
| NM_006206 | PDGFRA | 9061 |  |
| NM_002609 | PDGFRB | 23178 |  |
| NM_006207 | PDGFRL | 5105 |  |
| NM_152835 | PDIK1L | 5106 |  |
| NM_002610 | PDK1 | 5127 |  |
| NM_002611 | PDK2 | 5128 | CCACGUACCGCGUCAGCUA |
| NM_005391 | PDK3 | 5156 |  |
| NM_002612 | PDK4 | 5159 |  |
| NM_031268 | PDPK1 | 149420 |  |
| NM_003681 | PDXK | 149420 |  |
| NM_002625 | PFKFB1 | 5163 |  |
| NM_001018053 | PFKFB2 | 5165 |  |
| NM_004566 | PFKFB3 | 5166 |  |
| NM_004567 | PFKFB4 | 5170 |  |
| NM_002626 | PFKL | 8566 |  |
| NM_000289 | PFKM | 5207 |  |
| NM_002627 | PFKP | 5208 |  |
| NM_012395 | PFTK1 | 5209 |  |
| NM_000291 | PGK1 | 5210 |  |
| NM_138733 | PGK2 | 5211 |  |
| NM_002637 | PHKA1 | 5213 |  |
| NM_000292 | PHKA2 | 5230 |  |
| NM_000293 | PHKB | 5232 |  |
| NM_006213 | PHKG1 | 5255 |  |
| NM_000294 | PHKG2 | 5256 |  |
| NM_018323 | PI4K2B | 5260 |  |
| NM_018425 | PI4KII | 5261 |  |
| NM_002645 | PIK3C2A | 55300 |  |
| NM_002646 | PIK3C2B | 55361 |  |
| NM_004570 | PIK3C2G | 55361 |  |
| NM_002647 | PIK3C3 | 5286 |  |
| NM_006218 | PIK3CA | 5287 |  |
| NM_006219 | PIK3CB | 5288 |  |
| NM_005026 | PIK3CD | 5288 |  |
| NM_002649 | PIK3CG | 5289 |  |
| NM_181504 | PIK3R1 | 5291 |  |
| NM_005027 | PIK3R2 | 5293 |  |
| NM_003629 | PIK3R3 | 5295 |  |
| NM_014602 | PIK3R4 | 5296 |  |
| NM_002650 | PIK4CA | 8503 |  |
| NM_002651 | PIK4CB | 30849 |  |
| NM_002648 | PIM1 | 5297 |  |
| NM_006875 | PIM2 | 5298 |  |
| NM_001001852 | PIM3 | 5298 |  |
| NM_032409 | PINK1 | 5292 |  |
| NM_003557 | PIP5K1A | 5292 |  |
| NM_003558 | PIP5K1B | 11040 |  |
| NM_012398 | PIP5K1C | 415116 |  |
| NM_005028 | PIP5K2A | 65018 |  |
| NM_003559 | PIP5K2B | 8394 |  |
| NM_024779 | PIP5K2C | 8395 |  |
| NM_152671 | PIP5K3 | 23396 |  |
| NM_173492 | PIP5KL1 | 5305 |  |
| NM_006823 | PKIA | 5305 |  |
| NM_032471 | PKIB | 8396 |  |
| NM_181871 | PKLR | 79837 |  |
| NM_002654 | PKM2 | 200576 |  |
| NM_004203 | PKMYT1 | 138429 |  |
| NM_013355 | PKN3 | 5569 | CUUCUGCGAUCCUGUCAUU |
| NM_005030 | PLK1 | 5570 |  |
| NM_006622 | PLK2 | 5313 |  |
| NM_004073 | PLK3 | 5315 |  |
| NM_014264 | PLK4 | 9088 | GAAGAUAGCAAUUAUGUGU |
| NM_006556 | PMVK | 29941 |  |
| NM_001039582 | PNCK | 5347 |  |
| NM_007254 | PNKP | 10769 |  |
| NM_006251 | PRKAA1 | 1263 |  |
| NM_006252 | PRKAA2 | 10733 |  |
| NM_006253 | PRKAB1 | 10733 |  |
| NM_005399 | PRKAB2 | 10654 |  |
| NM_207518 | PRKACA | 139728 |  |
| NM_002731 | PRKACB | 11284 |  |
| NM_002732 | PRKACG | 11284 |  |
| NM_002733 | PRKAG1 | 5563 |  |
| NM_024429 | PRKAG2 | 5564 |  |
| NM_017431 | PRKAG3 | 5565 | GACCAGCAGCUCAGAAAGA |
| NM_002734 | PRKAR1A | 5566 |  |
| NM_002735 | PRKAR1B | 5567 |  |
| NM_004157 | PRKAR2A | 5568 |  |
| NM_002736 | PRKAR2B | 5571 |  |
| NM_002737 | PRKCA | 5571 |  |
| NM_002738 | PRKCB1 | 51422 |  |
| NM_006254 | PRKCD | 53632 |  |
| NM_005400 | PRKCE | 5573 |  |
| NM_002739 | PRKCG | 5575 |  |
| NM_006255 | PRKCH | 5576 |  |
| NM_002740 | PRKCI | 5577 |  |
| NM_002741 | PRKCL1 | 5578 |  |
| NM_006256 | PRKCL2 | 5579 |  |
| NM_002742 | PRKCM | 5580 |  |
| NM_005813 | PRKCN | 5582 |  |
| NM_006257 | PRKCQ | 5583 |  |
| NM_002743 | PRKCSH | 5584 |  |
| NM_002744 | PRKCZ | 5586 |  |
| NM_001079882 | PRKD2 | 5586 |  |
| NM_006904 | PRKDC | 5587 |  |
| NM_006258 | PRKG1 | 5587 |  |
| NM_006259 | PRKG2 | 23683 |  |
| NM_002759 | PRKR | 5588 |  |
| NM_018979 | PRKWNK1 | 5589 |  |
| NM_006648 | PRKWNK2 | 5590 |  |
| NM_001002838 | PRKWNK3 | 25865 |  |
| NM_005044 | PRKX | 5591 |  |
| NM_002760 | PRKY | 5593 |  |
| NM_003913 | PRPF4B | 5593 |  |
| NM_002764 | PRPS1 | 5610 |  |
| NM_175886 | PRPS1L1 | 65125 |  |
| NM_002765 | PRPS2 | 65268 |  |
| NM_006742 | PSKH1 | 65267 |  |
| NM_033126 | PSKH2 | 5613 |  |
| NM_005607 | PTK2 | 5616 |  |
| NM_004103 | PTK2B | 8899 |  |
| NM_005975 | PTK6 | 5631 |  |
| NM_152881 | PTK7 | 221823 |  |
| NM_002822 | PTK9 | 5634 |  |
| NM_007284 | PTK9L | 85481 |  |
| NM_017771 | PXK | 5747 |  |
| NM_001017423 | PYCS | 2185 |  |
| NM_002880 | RAF1 | 5753 |  |
| NM_014226 | RAGE | 5754 |  |
| NM_022128 | RBKS | 11344 |  |
| NM_021975 | RELA | 54899 |  |
| NM_020630 | RET | 5832 |  |
| NM_018339 | RFK | 5894 |  |
| NM_006510 | RFP | 5891 |  |
| NM_031480 | RIOK1 | 64080 |  |
| NM_018343 | RIOK2 | 5970 |  |
| NM_003831 | RIOK3 | 5970 |  |
| NM_003804 | RIPK1 | 5979 |  |
| NM_003821 | RIPK2 | 55312 |  |
| NM_006871 | RIPK3 | 5987 |  |
| NM_021133 | RNASEL | 8780 |  |
| NM_005406 | ROCK1 | 8737 |  |
| NM_004850 | ROCK2 | 8737 |  |
| NM_001083592 | ROR1 | 11035 |  |
| NM_004560 | ROR2 | 6041 |  |
| NM_002944 | ROS1 | 6093 |  |
| NM_001042452 | RP6-213H19.1 | 9475 |  |
| NM_001006665 | RPS6KA1 | 4919 |  |
| NM_001006932 | RPS6KA2 | 4920 |  |
| NM_004586 | RPS6KA3 | 6098 |  |
| NM_001006944 | RPS6KA4 | 51765 |  |
| NM_182398 | RPS6KA5 | 6195 |  |
| NM_014496 | RPS6KA6 | 6196 |  |
| NM_003161 | RPS6KB1 | 6197 |  |
| NM_003952 | RPS6KB2 | 6197 |  |
| NM_012424 | RPS6KC1 | 9252 |  |
| NM_031464 | RPS6KL1 | 27330 |  |
| NM_002958 | RYK | 6198 |  |
| NM_014975 | SAST | 26750 |  |
| NM_001024401 | SBK1 | 83694 |  |
| NM_001075099 | SCAP1 | 6259 |  |
| NM_001048218 | SCYL1 | 22983 |  |
| NM_005627 | SGK | 388228 |  |
| NM_016276 | SGK2 | 8631 |  |
| NM_013257 | SGKL | 57410 | GAAAGCUGCCCAAGUGUAA |
| NM_015191 | SIK2 | 6446 |  |
| NM_014720 | SLK | 23678 |  |
| NM_015092 | SMG1 | 23235 |  |
| NM_030952 | SNARK | 9748 |  |
| NM_173354 | SNF1LK | 23049 |  |
| NM_001100594 | SNRK | 81788 |  |
| NM_005876 | SPEG | 10290 |  |
| NM_021972 | SPHK1 | 8877 |  |
| NM_020126 | SPHK2 | 56848 |  |
| NM_005417 | SRC | 6725 |  |
| NM_080823 | SRMS | 6731 |  |
| NM_006947 | SRP72 | 6732 |  |
| NM_003137 | SRPK1 | 6733 |  |
| NM_182691 | SRPK2 | 83983 |  |
| NM_032037 | SSTK | 6793 |  |
| NM_005990 | STK10 | 6793 |  |
| NM_000455 | STK11 | 6794 |  |
| NM_001008910 | STK16 | 8576 |  |
| NM_004760 | STK17A | 9263 |  |
| NM_004226 | STK17B | 8859 |  |
| NM_004197 | STK19 | 23617 |  |
| NM_053006 | STK22B | 81629 |  |
| NM_052841 | STK22C | 81629 |  |
| NM_032028 | STK22D | 83942 |  |
| NM_014370 | STK23 | 83942 |  |
| NM_003576 | STK24 | 26576 |  |
| NM_006374 | STK25 | 9024 |  |
| NM_003957 | STK29 | 6788 |  |
| NM_006281 | STK3 | 56164 |  |
| NM_031414 | STK31 | 202374 |  |
| NM_145001 | STK32A | 55351 |  |
| NM_018401 | STK32B | 282974 |  |
| NM_173575 | STK32C | 65975 |  |
| NM_030906 | STK33 | 140901 |  |
| NM_080836 | STK35 | 27148 |  |
| NM_015690 | STK36 | 11329 |  |
| NM_007271 | STK38 | 23012 |  |
| NM_015000 | STK38L | 27347 |  |
| NM_013233 | STK39 | 6789 |  |
| NM_006282 | STK4 | 55359 |  |
| NM_018423 | STYK1 | 6850 |  |
| NM_003177 | SYK | 6872 |  |
| NM_004606 | TAF1 | 138474 |  |
| NM_153809 | TAF1L | 138474 |  |
| NM_004783 | TAO1 | 9344 |  |
| NM_013254 | TBK1 | 29110 |  |
| NM_003215 | TEC | 7006 |  |
| NM_000459 | TEK | 7010 |  |
| NM_006285 | TESK1 | 7016 |  |
| NM_007170 | TESK2 | 10420 |  |
| NM_031272 | TEX14 | 56155 |  |
| NM_004612 | TGFBR1 | 7046 |  |
| NM_003242 | TGFBR2 | 7048 |  |
| NM_003243 | TGFBR3 | 7049 |  |
| NM_024838 | THNSL1 | 79896 |  |
| NM_201629 | TJP2 | 9414 |  |
| NM_004614 | TK2 | 7084 |  |
| NM_012290 | TLK1 | 9874 |  |
| NM_006852 | TLK2 | 11011 |  |
| NM_015028 | TNIK | 23043 |  |
| NM_003985 | TNK1 | 8711 |  |
| NM_005781 | TNK2 | 10188 |  |
| NM_015978 | TNNI3K | 10188 |  |
| NM_018492 | TOPK | 55872 |  |
| NM_033550 | TP53RK | 112858 |  |
| NM_001042482 | TPK1 | 27010 | GCCAACCGCUUAUAUGAUA |
| NM_025195 | TRIB1 | 10221 |  |
| NM_021643 | TRIB2 | 28951 |  |
| NM_021158 | TRIB3 | 57761 |  |
| NM_007118 | TRIO | 54822 |  |
| NM_017662 | TRPM6 | 60385 |  |
| NM_017672 | TRPM7 | 84630 |  |
| NM_021733 | TSKS | 146057 |  |
| NM_032538 | TTBK1 | 7272 |  |
| NM_173500 | TTBK2 | 7297 |  |
| NM_003318 | TTK | 7301 |  |
| NM_003331 | TYK2 | 7301 |  |
| NM_006293 | TYRO3 | 83549 |  |
| NM_031432 | UCK1 | 127933 |  |
| NM_175866 | UHMK1 | 127933 |  |
| NM_003565 | ULK1 | 8408 |  |
| NM_014683 | ULK2 | 9706 |  |
| NM_017886 | ULK4 | 54986 |  |
| NM_016308 | UMP-CMPK | 51727 |  |
| NM_012474 | UMPK | 7371 |  |
| NM_017859 | URKL1 | 7443 |  |
| NM_003384 | VRK1 | 7444 |  |
| NM_006296 | VRK2 | 51231 |  |
| NM_001025778 | VRK3 | 7465 |  |
| NM_003390 | WEE1 | 65266 |  |
| NM_032387 | WNK4 | 9942 |  |
| NM_005108 | XYLB | 7525 |  |
| NM_005433 | YES1 | 7525 |  |
| NM_016653 | ZAK | 51776 |  |
| NM_001079 | ZAP70 | 7535 |  |
