# Supplementary material for: Identification of Host Kinase Genes Required for Influenza Virus Replication and the Regulatory Role of MicroRNAs
Source: PLoS One. 2013 Jun 21;8(6):e66796. doi: 10.1371/journal.pone.0066796 (PMC3689682; doi:10.1371/journal.pone.0066796)
Supplement: Table S3 — Summary of hits overlapping with other influenza whole genome siRNA screens. Data from other screens to identify host genes crucial for influenza virus replication were compared with the hits in our screen and are highlighted in red. (DOCX) [file pone.0066796.s009.docx]

| **Supplemental Table S3. Overlap with other published influenza screens.** | | | | | | | |
| --- | --- | --- | --- | --- | --- | --- | --- |
| **Hit Gene*** | Brass, A.L. et al. Cell 139, 1243-1254 (2009). | Shapira, S.D. et al. Cell 139, 1255-1267 (2009). | Konig, R. et al. Nature 463, 813-817 (2010). | Karlas, A. et al. Nature 463, 818-822 (2010). | Hao, L. et al. Nature 454, 890-893 (2008).** | Andersen, L.E. et al. | **Total** |
| COPA | 1 | 1 | 1 | 1 |  |  | 4 |
| COPG | 1 |  | 1 | 1 | 1 |  | 4 |
| NXF1 | 1 | 1 |  | 1 | 1 |  | 4 |
| ARCN1 | 1 |  | 1 |  | 1 |  | 3 |
| ATP6AP1 | 1 |  | 1 | 1 |  |  | 3 |
| ATP6V0C |  |  | 1 | 1 | 1 |  | 3 |
| ATP6V0D1 |  |  | 1 | 1 | 1 |  | 3 |
| COPB2 | 1 |  | 1 | 1 |  |  | 3 |
| NUP98 | 1 |  |  | 1 | 1 |  | 3 |
| PGD | 1 | 1 |  |  | 1 |  | 3 |
| PRPF8 | 1 | 1 |  | 1 |  |  | 3 |
| RPS10 |  |  | 1 | 1 | 1 |  | 3 |
| RPS16 | 1 |  |  | 1 | 1 |  | 3 |
| **CLK1** |  |  | 1 | 1 |  |  | 2 |
| **PRKACA** |  | 1 | 1 |  |  |  | 2 |
| AQR | 1 |  |  |  | 1 |  | 2 |
| ARTN |  | 1 |  | 1 |  |  | 2 |
| ATP6AP2 |  |  | 1 | 1 |  |  | 2 |
| ATP6V0B | 1 |  | 1 |  |  |  | 2 |
| ATP6V1A |  |  | 1 | 1 |  |  | 2 |
| ATP6V1B2 |  |  | 1 | 1 |  |  | 2 |
| B2M |  | 1 |  | 1 |  |  | 2 |
| BUB3 | 1 | 1 |  |  |  |  | 2 |
| BZRAP1 |  |  | 1 | 1 |  |  | 2 |
| C14orf109 | 1 | 1 |  |  |  |  | 2 |
| C6orf62 | 1 | 1 |  |  |  |  | 2 |
| CALCOCO2 | 1 | 1 |  |  |  |  | 2 |
| **CALM2** |  | 1 |  |  |  | 1 | 2 |
| **CAMK2B** |  | 1 | 1 |  |  |  | 2 |
| CD81 |  |  | 1 | 1 |  |  | 2 |
| **CDK4** |  | 1 | 1 |  |  |  | 2 |
| CFLAR |  | 1 | 1 |  |  |  | 2 |
| CLIC4 |  | 1 |  | 1 |  |  | 2 |
| COPB1 | 1 |  |  | 1 |  |  | 2 |
| DCLK2 |  |  | 1 | 1 |  |  | 2 |
| DLG5 |  | 1 | 1 |  |  |  | 2 |
| DMAP1 |  |  |  | 1 | 1 |  | 2 |
| EIF2AK2 |  | 1 | 1 |  |  |  | 2 |
| EIF4A2 | 1 |  |  |  | 1 |  | 2 |
| **EPHB2** |  | 1 | 1 |  |  |  | 2 |
| FAM38A | 1 | 1 |  |  |  |  | 2 |
| FAU |  |  |  | 1 | 1 |  | 2 |
| **FGFR2** |  | 1 | 1 |  |  |  | 2 |
| FLNC | 1 | 1 |  |  |  |  | 2 |
| HAND2 |  |  | 1 |  | 1 |  | 2 |
| **HK2** |  | 1 |  |  |  | 1 | 2 |
| IFIT5 | 1 | 1 |  |  |  |  | 2 |
| IFITM3 | 1 | 1 |  |  |  |  | 2 |
| **IKBKE** |  | 1 | 1 |  |  |  | 2 |
| IL17RA |  |  | 1 | 1 |  |  | 2 |
| IRF2 |  | 1 |  | 1 |  |  | 2 |
| ISG15 |  | 1 |  | 1 |  |  | 2 |
| **ITPKB** |  | 1 |  |  |  | 1 | 2 |
| JUN |  |  | 1 | 1 |  |  | 2 |
| KPNB1 |  |  | 1 | 1 |  |  | 2 |
| KRTCAP2 | 1 |  |  |  | 1 |  | 2 |
| **MAP2K3** |  | 1 | 1 |  |  |  | 2 |
| **MAP3K12** |  | 1 | 1 |  |  |  | 2 |
| **MAPK1** |  | 1 | 1 |  |  |  | 2 |
| MFAP1 | 1 | 1 |  |  |  |  | 2 |
| MYC |  | 1 |  | 1 |  |  | 2 |
| **NEK8** |  |  |  |  | 1 | 1 | 2 |
| NHP2L1 | 1 |  | 1 |  |  |  | 2 |
| NUP153 |  |  | 1 |  | 1 |  | 2 |
| OSMR |  | 1 | 1 |  |  |  | 2 |
| **PANK4** |  |  | 1 |  |  | 1 | 2 |
| PHF2 |  |  | 1 | 1 |  |  | 2 |
| **PIK3R4** |  | 1 | 1 |  |  |  | 2 |
| **PLK3** |  |  | 1 | 1 |  |  | 2 |
| **PLK4** |  |  | 1 |  |  | 1 | 2 |
| PLXNA2 |  | 1 |  |  | 1 |  | 2 |
| PPAN | 1 | 1 |  |  |  |  | 2 |
| PPP1R14D |  |  | 1 | 1 |  |  | 2 |
| PSENEN |  |  | 1 | 1 |  |  | 2 |
| PSMD11 |  | 1 |  |  | 1 |  | 2 |
| PTPRN |  |  | 1 | 1 |  |  | 2 |
| PTS | 1 |  | 1 |  |  |  | 2 |
| RAB5A | 1 |  |  |  | 1 |  | 2 |
| RACGAP1 |  |  | 1 | 1 |  |  | 2 |
| RBCK1 |  | 1 | 1 |  |  |  | 2 |
| RPL13A | 1 | 1 |  |  |  |  | 2 |
| RPS14 |  |  |  | 1 | 1 |  | 2 |
| RPS20 |  |  | 1 |  | 1 |  | 2 |
| RPS4X | 1 |  |  |  | 1 |  | 2 |
| RPS5 |  |  |  | 1 | 1 |  | 2 |
| RUNX1 | 1 |  |  | 1 |  |  | 2 |
| SF3A1 |  |  | 1 | 1 |  |  | 2 |
| SF3B1 | 1 |  |  | 1 |  |  | 2 |
| SLC1A3 | 1 | 1 |  |  |  |  | 2 |
| STARD5 | 1 | 1 |  |  |  |  | 2 |
| SUPT6H |  | 1 |  | 1 |  |  | 2 |
| TNFRSF18 |  | 1 |  | 1 |  |  | 2 |
| **TNK2** |  |  | 1 | 1 |  |  | 2 |
| TRIM21 |  | 1 |  | 1 |  |  | 2 |
| TRIM28 | 1 |  | 1 |  |  |  | 2 |
| VCP |  | 1 |  |  | 1 |  | 2 |
| WDR18 | 1 |  | 1 |  |  |  | 2 |
| **AMHR2** |  | 1 |  |  |  |  | 1 |
| **CAMK2G** |  | 1 |  |  |  |  | 1 |
| **CCRK** |  |  | 1 |  |  |  | 1 |
| **CDC42BPB** |  |  | 1 |  |  |  | 1 |
| **CLK4** |  | 1 |  |  |  |  | 1 |
| **CSNK2A2** |  | 1 |  |  |  |  | 1 |
| **DAPK2** |  |  | 1 |  |  |  | 1 |
| **DAPK3** |  |  | 1 |  |  |  | 1 |
| **DGKD** |  | 1 |  |  |  |  | 1 |
| **DGKQ** |  | 1 |  |  |  |  | 1 |
| **GAK** | 1 |  |  |  |  |  | 1 |
| **HIPK3** |  |  | 1 |  |  |  | 1 |
| **IKBKG** |  | 1 |  |  |  |  | 1 |
| **IRAK3** |  |  | 1 |  |  |  | 1 |
| **LTK** |  |  | 1 |  |  |  | 1 |
| **MAP2K1** |  | 1 |  |  |  |  | 1 |
| **MAP2K7** |  | 1 |  |  |  |  | 1 |
| **MAP3K11** |  |  | 1 |  |  |  | 1 |
| **MAP3K9** |  | 1 |  |  |  |  | 1 |
| **MAPK14** |  | 1 |  |  |  |  | 1 |
| **MKNK1** |  | 1 |  |  |  |  | 1 |
| **NEK6** |  |  |  | 1 |  |  | 1 |
| **PCK2** |  | 1 |  |  |  |  | 1 |
| **PRKCA** |  | 1 |  |  |  |  | 1 |
| **PRKCD** |  |  | 1 |  |  |  | 1 |
| **RIPK2** |  | 1 |  |  |  |  | 1 |
| **RPS6KA2** |  |  | 1 |  |  |  | 1 |
| **RPS6KL1** | 1 |  |  |  |  |  | 1 |
| **STK38** |  | 1 |  |  |  |  | 1 |
| *Kinases shown in red.  **Only showing those with human orthologues. | | | | | | | |
