# Supplementary material for: Identification of Host Kinase Genes Required for Influenza Virus Replication and the Regulatory Role of MicroRNAs
Source: PLoS One. 2013 Jun 21;8(6):e66796. doi: 10.1371/journal.pone.0066796 (PMC3689682; doi:10.1371/journal.pone.0066796)
Supplement: Table S4 — Oligonucleotide sequences of all primers used in the manuscript. Table lists the genes and the primers used for qPCR in this manuscript. Forward primers are appended with F and reverse primers are appended with R. (DOCX) [file pone.0066796.s010.docx]

| **Supplemental Table S4. Primer sequences** | |
| --- | --- |
| **PRIMER** | **SEQUENCE** |
| NPR2-F | AAC TTG GAG AAG CTG GTG GAG GAA |
| NPR2-R | CTG CTG ACA ATG CTG TGA AGC CAA |
| NEK3-F | TTG GTT CAG CAT GAA AGC AG |
| NEK3-R | TGC ATT AGA TCC CCT CCA TC |
| EPHA6-F | GGC AGG TGA ATT TGG AGA AG |
| EPHA6-R | ACC CCT TCT AGG CGA ATG AT |
| EXOSC10-F | AGA GAG AGC GAG CAA CAA GC |
| EXOSC10-R | TCC AGC AAA AGC CTT GAA GT |
| ITPKB-F | GAT CGA CTT TGG GAA AAC CA |
| ITPKB-R | ACG AGA AAG GAA GCA CAG GA |
| PLK4-F | GGT CAG CCA CTC CCA AAT AA |
| PLK4-R | GGC CTT TCT TCT GCA TCT TG |
| CALM2-F | TGG CTG ACC AAC TGA CTG AA |
| CALM2-R | TGT GCC ATT ACC ATC AGC AT |
| ITPK1-F | CTG GAG AAG AAC GGC TTG AC |
| ITPK1-R | CTG GAC CAC GGT GTA GGA CT |
| PDK2-F | ATG GCA GTC CTC CTC TCT GA |
| PDK2-R | CAC CCA CCC TCT TCC TAA CA |
| CDK3-F | ACG GTG CCG TTT CAA AAT AG |
| CDK3-R | CTC AAC TCT TCC CCC AAA CA |
| CDC2L5-F | CTC GAA TTC TGC CTC CTG AC |
| CDC2L5-R | TCT CTT TTG GGG TCA TCC TG |
| SGKL-F | AGC TGC CCA AGT GTA AGC AT |
| SGKL-R | CAG GAA TCT TCA GGG CCA TA |
| MAP3K1-F | TGA ACA GCT ATG AAC GAG GCC AGT |
| MAP3K1-R | TTT CCT GTT CAC CTA GGG CCA GTT |
| DYRK3-F | TTG GTG GTC CCA ATA ATG GAG GGT |
| DYRK3-R | TAG GGC CAC GTA CTG TCG AAG TTT |
| PANK4-F | TGC GCA CCA TCA CCT ATA GCA TCA |
| PANK4-R | AGG ATT GTC CTG CTC AGC TCC TTT |
| TPK1-F | TCC TAC CAG CCA TTG TAG GCC AAT |
| TPK1-R | TCA TAT AAG CGG TTG GCA CCT CCA |
| NEK8-F | ATG ACT TGC AGC TGT CTC CTG GAT |
| NEK8-R | AAG GGC AAA GAG GTG GTA GGA GAA |
| C9ORF96-F | AAT GCG CAC CTC ACA CTC CTC TTA |
| C9ORF96-R | AAT GGT CTG GAA GGA CTG CTC TGT |
| PRKAG3-F | ACA CAC AAA CGC CTG CTC AAG TTC |
| PRKAG3-R | GGA ATG TGC CGA TGC CCA AAT CTT |
| ERBB4-F | TCT GCA TGG GTT GGT CAG GAG AAT |
| ERBB4-R | GCA TGG GTG TTT CAA CCA TCT GCT |
| ADK-F | AGA GGC AGC GAA TCG TGA TCT TCA |
| ADK-R | ACC TCC AAC AAA TGC ATC TCC AGC |
| PKN3-F | ACG CCA TCA AAG CAC TGA AGA AGC |
| PKN3-R | ATC TGC ATC ATG AGG TCA CCA CCA |
| HK2-F | TGC AGC GCA TCA AGG AGA ACA AAG |
| HK2-R | ACG GTC TTA TGT AGA CGC TTG GCA |
| GAPDH-F | GGT ATC GTG GAA GGA CTC ATG AC |
| GAPDH-R | ATG CCA GTG AGC TTC CCG TTC AG |
| MAP2K1-F | GCC CTC CAA CAT CCT AGT CA |
| MAP2K1-R | GAC AGT CCC ATG CTC CAG AT |
