# Supplementary material for: Identification of Host Kinase Genes Required for Influenza Virus Replication and the Regulatory Role of MicroRNAs
Source: PLoS One. 2013 Jun 21;8(6):e66796. doi: 10.1371/journal.pone.0066796 (PMC3689682; doi:10.1371/journal.pone.0066796)
Supplement: Text S1 — Alignment of miR-548 family miRNA sequences. Sequences of miR-548 family members were aligned using Clustal W application locally in BioEdit ver. 7.0 (Tom Hall). Alignment shows high degree of seed identity between multiple miR-548 members. (RTF) [file pone.0066796.s013.rtf]

                                  10         20            
                         ....|....| ....|....| ....|
hsa-miR-548a-3p MIMA  1  CAAAACUGGC AAUUACUUUU GC    22 
hsa-miR-548a-5p MIMA  1  AAAAGUAAUU GCGAGUUUUA CC    22 
hsa-miR-548aa MIMAT0  1  AAAAACCACA AUUACUUUUG CACCA 25 
hsa-miR-548ab MIMAT0  1  AAAAGUAAUU GUGGAUUUUG CU    22 
hsa-miR-548ac MIMAT0  1  CAAAAACCGG CAAUUACUUU UG    22 
hsa-miR-548ad MIMAT0  1  GAAAACGACA AUGACUUUUG CA    22 
hsa-miR-548ae MIMAT0  1  CAAAAACUGC AAUUACUUUC A     21 
hsa-miR-548ag MIMAT0  1  AAAGGUAAUU GUGGUUUCUG C     21 
hsa-miR-548ah-3p MIM  1  CAAAAACUGC AGUUACUUUU GC    22 
hsa-miR-548ah-5p MIM  1  AAAAGUGAUU GCAGUGUUUG       20 
hsa-miR-548ai MIMAT0  1  AAAGGUAAUU GCAGUUUUUC CC    22 
hsa-miR-548aj-3p MIM  1  UAAAAACUGC AAUUACUUUU A     21 
hsa-miR-548aj-5p MIM  1  UGCAAAAGUA AUUGCAGUUU UUG   23 
hsa-miR-548ak MIMAT0  1  AAAAGUAACU GCGGUUUUUG A     21 
hsa-miR-548al MIMAT0  1  AACGGCAAUG ACUUUUGUAC CA    22 
hsa-miR-548am-3p MIM  1  CAAAAACUGC AGUUACUUUU GU    22 
hsa-miR-548am-5p MIM  1  AAAAGUAAUU GCGGUUUUUG CC    22 
hsa-miR-548an MIMAT0  1  AAAAGGCAUU GUGGUUUUUG       20 
hsa-miR-548ao-3p MIM  1  AAAGACCGUG ACUACUUUUG CA    22 
hsa-miR-548ao-5p MIM  1  AGAAGUAACU ACGGUUUUUG CA    22 
hsa-miR-548ap-3p MIM  1  AAAAACCACA AUUACUUUU        19 
hsa-miR-548ap-5p MIM  1  AAAAGUAAUU GCGGUCUUU        19 
hsa-miR-548aq-3p MIM  1  CAAAAACUGC AAUUACUUUU GC    22 
hsa-miR-548aq-5p MIM  1  GAAAGUAAUU GCUGUUUUUG CC    22 
hsa-miR-548ar-3p MIM  1  UAAAACUGCA GUUAUUUUUG C     21 
hsa-miR-548ar-5p MIM  1  AAAAGUAAUU GCAGUUUUUG C     21 
hsa-miR-548as-3p MIM  1  UAAAACCCAC AAUUAUGUUU GU    22 
hsa-miR-548as-5p MIM  1  AAAAGUAAUU GCGGGUUUUG CC    22 
hsa-miR-548at-3p MIM  1  CAAAACCGCA GUAACUUUUG U     21 
hsa-miR-548at-5p MIM  1  AAAAGUUAUU GCGGUUUUGG CU    22 
hsa-miR-548au-3p MIM  1  UGGCAGUUAC UUUUGCACCA G     21 
hsa-miR-548au-5p MIM  1  AAAAGUAAUU GCGGUUUUUG C     21 
hsa-miR-548av-3p MIM  1  AAAACUGCAG UUACUUUUGC       20 
hsa-miR-548av-5p MIM  1  AAAAGUACUU GCGGAUUU         18 
hsa-miR-548aw MIMAT0  1  GUGCAAAAGU CAUCACGGUU       20 
hsa-miR-548ax MIMAT0  1  AGAAGUAAUU GCGGUUUUGC CA    22 
hsa-miR-548b-3p MIMA  1  CAAGAACCUC AGUUGCUUUU GU    22 
hsa-miR-548b-5p MIMA  1  AAAAGUAAUU GUGGUUUUGG CC    22 
hsa-miR-548c-3p MIMA  1  CAAAAAUCUC AAUUACUUUU GC    22 
hsa-miR-548c-5p MIMA  1  AAAAGUAAUU GCGGUUUUUG CC    22 
hsa-miR-548d-3p MIMA  1  CAAAAACCAC AGUUUCUUUU GC    22 
hsa-miR-548d-5p MIMA  1  AAAAGUAAUU GUGGUUUUUG CC    22 
hsa-miR-548e MIMAT00  1  AAAAACUGAG ACUACUUUUG CA    22 
hsa-miR-548f MIMAT00  1  AAAAACUGUA AUUACUUUU        19 
hsa-miR-548g-3p MIMA  1  AAAACUGUAA UUACUUUUGU AC    22 
hsa-miR-548g-5p MIMA  1  UGCAAAAGUA AUUGCAGUUU UUG   23 
hsa-miR-548h-3p MIMA  1  CAAAAACCGC AAUUACUUUU GCA   23 
hsa-miR-548h-5p MIMA  1  AAAAGUAAUC GCGGUUUUUG UC    22 
hsa-miR-548i MIMAT00  1  AAAAGUAAUU GCGGAUUUUG CC    22 
hsa-miR-548j MIMAT00  1  AAAAGUAAUU GCGGUCUUUG GU    22 
hsa-miR-548k MIMAT00  1  AAAAGUACUU GCGGAUUUUG CU    22 
hsa-miR-548l MIMAT00  1  AAAAGUAUUU GCGGGUUUUG UC    22 
hsa-miR-548m MIMAT00  1  CAAAGGUAUU UGUGGUUUUU G     21 
hsa-miR-548n MIMAT00  1  CAAAAGUAAU UGUGGAUUUU GU    22 
hsa-miR-548o-3p MIMA  1  CCAAAACUGC AGUUACUUUU GC    22 
hsa-miR-548o-5p MIMA  1  AAAAGUAAUU GCGGUUUUUG CC    22 
hsa-miR-548p MIMAT00  1  UAGCAAAAAC UGCAGUUACU UU    22 
hsa-miR-548q MIMAT00  1  GCUGGUGCAA AAGUAAUGGC GG    22 
hsa-miR-548s MIMAT00  1  AUGGCCAAAA CUGCAGUUAU UUU   23 
hsa-miR-548t-3p MIMA  1  AAAAACCACA AUUACUUUUG CACCA 25 
hsa-miR-548t-5p MIMA  1  CAAAAGUGAU CGUGGUUUUU G     21 
hsa-miR-548u MIMAT00  1  CAAAGACUGC AAUUACUUUU GCG   23 
hsa-miR-548v MIMAT00  1  AGCUACAGUU ACUUUUGCAC CA    22 
hsa-miR-548w MIMAT00  1  AAAAGUAACU GCGGUUUUUG CCU   23 
hsa-miR-548x-3p MIMA  1  UAAAAACUGC AAUUACUUUC       20 
hsa-miR-548x-5p MIMA  1  UGCAAAAGUA AUUGCAGUUU UUG   23 
hsa-miR-548y MIMAT00  1  AAAAGUAAUC ACUGUUUUUG CC    22 
hsa-miR-548z MIMAT00  1  CAAAAACCGC AAUUACUUUU GCA   23 
